# Universal coverage for oral health care in 27 low-income countries: a scoping review

**DOI:** 10.1186/s41256-024-00376-9

**Published:** 2024-09-10

**Authors:** Yiqun Luan, Divesh Sardana, Ashiana Jivraj, David Liu, Nishmi Abeyweera, Yajin Zhao, Jacqueline Cellini, Michelle Bass, Jing Wang, Xinran Lu, Zheyi Cao, Chunling Lu

**Affiliations:** 1 Heller School for Social Policy and Management, Brandeis University, Waltham, USA; 2 Department of Developmental Sciences, The University of Oklahoma Health Sciences Center College of Dentistry, Oklahoma City, OK USA; 3 Harvard School of Dental Medicine, Cambridge, USA; 4 International Business School, Brandeis University, Waltham, USA; 5 Department of Economics, Harvard University, Cambridge, USA; 6 Department of Epidemiology, Harvard T.H. Chan School of Public Health, Boston, USA; 7 Countway Library, Harvard Medical School, Boston, USA; 8 Department of Global Health and Population, Harvard T.H. Chan School of Public Health, Boston, USA; 9 Division of Global Health Equity, Brigham and Women’s Hospital, 641 Huntington Ave, Boston, MA 02115 USA; 10 Department of Global Health and Social Medicine, Harvard Medical School, 641 Huntington Ave, Boston, MA 02115 USA; 11 School of Dentistry and Riley Hospital for Children, Indiana University, Indianapolis, USA

**Keywords:** Oral health, Universal health coverage, Low-income countries

## Abstract

**Background:**

Low-income countries bear a growing and disproportionate burden of oral diseases. With the World Health Organization targeting universal oral health coverage by 2030, assessing the state of oral health coverage in these resource-limited nations becomes crucial. This research seeks to examine the political and resource commitments to oral health, along with the utilization rate of oral health services, across 27 low-income countries.

**Methods:**

We investigated five aspects of oral health coverage in low-income countries, including the integration of oral health in national health policies, covered oral health services, utilization rates, expenditures, and the number of oral health professionals. A comprehensive search was conducted across seven bibliographic databases, three grey literature databases, and national governments’ and international organizations’ websites up to May 2023, with no linguistic restrictions. Countries were categorized into “full integration”, “partial integration”, or “no integration” based on the presence of dedicated oral health policies and the frequency of oral health mentions. Covered oral health services, utilization rates, expenditure trends, and the density of oral health professionals were analyzed using evidence from reviews and data from World Health Organization databases.

**Results:**

A total of 4242 peer-reviewed and 3345 grey literature texts were screened, yielding 12 and 84 files respectively to be included in the final review. Nine countries belong to “full integration” and thirteen countries belong to “partial integration”, while five countries belong to “no integration”. Twelve countries collectively covered 26 types of oral health care services, with tooth extraction being the most prevalent service. Preventive and public health-based oral health interventions were scarce. Utilization rates remained low, with the primary motivation for seeking care being dental pain relief. Expenditures on oral health were minimal, predominantly relying on domestic private sources. On average, the 27 low-income countries had 0.51 dentists per 10,000 population, contrasting with 2.83 and 7.62 in middle-income and high-income countries.

**Conclusions:**

Oral health care received little political and resource commitment toward achieving universal health coverage in low-income countries. Urgent action is needed to mobilize financial and human resources, and integrate preventive and public health-based interventions.

**Supplementary Information:**

The online version contains supplementary material available at 10.1186/s41256-024-00376-9.

## Introduction

Oral diseases, such as dental caries, periodontal disease, and oral cancer, stand out as the most prevalent health conditions, posing a major global public health challenge [1]. It was estimated that approximately 3.5 billion people suffered from oral diseases worldwide [2]. Oral diseases could lead to pain, swelling, mastication difficulties, and susceptibility to other medical conditions such as atherosclerosis, cardiovascular disease, diabetes, and malnutrition [3–6]. Oral diseases can also affect self-esteem, reduce social interaction, and even result in stigmatization [7]. Moreover, they are closely linked to substantial productivity loss and lower educational attainments. Evidence indicates that the global productivity loss due to oral diseases reached an estimated US$ 323 billion in 2019 [7]; additionally, children with poor oral health are 52% more likely to exhibit poor academic performance and 43% more likely to experience school absenteeism [8].

Resources for oral health care and the burden of oral diseases are unevenly distributed across regions, countries, and socioeconomic groups [7]. In 2019, 88% of the direct costs of oral health care were spent for only 22.5% of the world’s population, mainly residing in high-income countries [7]. Meanwhile, more than three-quarters of those with oral conditions lived in low- and middle-income countries (LMICs) [7]. In Rwanda, for example, 54.3% of its population had untreated dental caries according to its first National Oral Health Survey [9]. Due to high out-of-pocket spending, disadvantaged households paying for necessary oral health care are more likely to experience catastrophic health expenditures and face impoverishment than their better-off counterparts [10, 11]. In addition, countries with public dental care coverage have lower socioeconomic inequalities in dental care utilization than those without such programs [12].

To tackle these challenges, the global community has launched a series of initiatives for oral health. In 2021, the World Health Assembly (WHA) adopted a resolution urging the integration of oral health into national policies and universal health coverage (UHC) [13]. This resolution was followed by the release of the Global Strategy on Oral Health during the 2022 WHA, which envisions achieving UHC for oral health for all individuals and communities by 2030 [14]. As a critical step towards implementing this strategy, the World Health Organization (WHO) released the Global Oral Health Action Plan (2023–2030), providing guidance for member states to operationalize objectives at the national and sub-national levels [15]. On December 15, 2023, the WHO added Noma (Cancrum Oris) to its list of neglected tropical diseases, marking a significant step toward prioritizing political and resource commitments for this severe and potentially fatal oral disease [16].

In addition to these initiatives, a notable effort is the WHO Global Oral Health Status Report [7]. This report reviews the status of oral disease burden, oral health policies, and resource investments over countries. While groundbreaking, the report and its country profiles only provide a summary of the existence of national oral health policies using a binary “yes-or-no” question without providing further details, such as the covered oral health services in their national policies. In addition, the report does not examine oral health service utilization rates or analyze oral health expenditures over the years. To address these gaps, especially for low-income countries, we conducted a scoping review to examine universal oral health coverage in low-income countries through five key questions: (1) the integration of oral health in national health policies; (2) the inclusion of clinical and public oral health care services in government-defined essential health service lists; (3) oral health care utilization rates; (4) oral health care expenditures; and (5) the number of oral health care professionals.

## Methods

### Search strategy

We conducted a scoping review to examine the universal coverage for oral health care in low-income countries. We followed the World Bank 2020 income classification [17] in identifying the 27 low-income countries (countries with gross national income [GNI] per capita of 1,045 US dollars or less) as listed in Table 1. We looked for evidence from peer-reviewed publications, grey literature, websites of national governments and international organizations, and WHO databases. We reported this scoping review in accordance with the guidance of the Preferred Reporting Items for Systematic Reviews and Meta-Analyses extension for Scoping Reviews (PRISMA-ScR) [18]. We registered the protocol of this scoping review a priori in Open Science Framework (https://osf.io/5pbhm/).

**Table 1:** 27 low-income countries included in this study

Afghanistan, Burkina Faso, Burundi, Central African Republic, Chad, Democratic Republic of the Congo (Congo DR), Eritrea, Ethiopia, Gambia, Guinea, Guinea-Bissau, Liberia, Madagascar, Malawi, Mali, Mozambique, Niger, North Korea, Rwanda, Sierra Leone, Somalia, South Sudan, Sudan, Syrian Arab Republic, Togo, Uganda, Yemen	

For peer-reviewed publications, we systematically searched seven bibliographic electronic databases: PubMed, Embase (Elsevier), Web of Science (Clarivate), EconLit (EBSCO), Global Health (EBSCO), WHO Global Index Medicus, and Dentistry and Oral Sciences Source. Two librarian coauthors (JC and MB) developed keyword search strings and adapted them for different databases. Table 2 shows the search string for PubMed. Search strings for other bibliographic databases are presented in Web Appendix 1. As shown in Table 2, the string consists of three groups of keywords: keywords and MeSH terms representing “oral”, “dental”, and “teeth”; keywords representing health care or the three UHC dimensions, such as “care”, “health”, “service”, “finance”, “coverage”, “spending”, “utilization”; and keywords and MeSH terms referring to the 27 low-income countries. We searched for the presence of keywords in titles and abstracts of publications. The search covered the period from January 2010 to May 2023 to capture the recent data.

**Table 2:** Search strings for PubMed

(("Dental Health Services"[Mesh] OR "Oral Health"[Mesh] OR "Dental Facilities"[Mesh]) OR (((dental[Title/Abstract] OR oral[Title/Abstract] OR teeth[Title/Abstract] OR tooth[Title/Abstract]) AND (care[Title/Abstract] OR health[Title/Abstract] OR service[Title/Abstract] OR access*[Title/Abstract] OR disparit*[Title/Abstract] OR coverage[Title/Abstract] OR insurance[Title/Abstract] OR utilization[Title/Abstract] OR spending[Title/Abstract] OR expenditure[Title/Abstract] OR financing[Title/Abstract] OR package[Title/Abstract] OR scheme[Title/Abstract] OR plan[Title/Abstract] OR strateg*[Title/Abstract] OR utilization[Title/Abstract])) OR dental facility[Title/Abstract] OR dental facilities[Title/Abstract] OR dental office[Title/Abstract] OR dental offices[Title/Abstract])) AND (((((((((((((((((((((((((((("Afghanistan"[Mesh]) OR "Burundi"[Mesh]) OR "Burkina Faso"[Mesh]) OR "Central African Republic"[Mesh]) OR "Chad"[Mesh]) OR "Democratic Republic of the Congo"[Mesh]) OR "Eritrea"[Mesh]) OR "Ethiopia"[Mesh]) OR "Gambia"[Mesh]) OR "Guinea"[Mesh]) OR "Guinea-Bissau"[Mesh]) OR "Liberia"[Mesh]) OR "Madagascar"[Mesh]) OR "Mali"[Mesh]) OR "Malawi"[Mesh]) OR "Mozambique"[Mesh]) OR "Niger"[Mesh]) OR "Democratic People's Republic of Korea"[Mesh]) OR "Rwanda"[Mesh]) OR "Sierra Leone"[Mesh]) OR "Somalia"[Mesh]) OR "Sudan"[Mesh]) OR "South Sudan"[Mesh]) OR "Syria"[Mesh]) OR "Togo"[Mesh]) OR "Uganda"[Mesh]) OR "Yemen"[Mesh]) OR (Afghanistan[Title/Abstract] OR Burundi[Title/Abstract] OR Burkina Faso[Title/Abstract] OR Central African Republic[Title/Abstract] OR Centrafrican Republic[Title/Abstract] OR Centrafique[Title/Abstract] OR Central African Empire[Title/Abstract] OR Chad[Title/Abstract] OR Congo[Title/Abstract] OR Democratic Republic of Congo[Title/Abstract] OR Eritrea[Title/Abstract] OR Ethiopia[Title/Abstract] OR Guinea[Title/Abstract] OR Gambia[Title/Abstract] OR Guinea Bissau[Title/Abstract] OR Guinea-Bissau[Title/Abstract] OR GuineaBissau[Title/Abstract] OR Liberia[Title/Abstract] OR Madagascar[Title/Abstract] OR Malagasy Republic[Title/Abstract] OR Mali[Title/Abstract] OR Malawi[Title/Abstract] OR Mozambique[Title/Abstract] OR Niger[Title/Abstract] OR North Korea[Title/Abstract] OR Democratic People's Republic of Korea[Title/Abstract] OR Korean People's Republic[Title/Abstract] OR Rwanda[Title/Abstract] OR Sierra Leone[Title/Abstract] OR Somalia[Title/Abstract] OR Sudan[Title/Abstract] OR South Sudan[Title/Abstract] OR Syria[Title/Abstract] OR Syrian Arab Republic[Title/Abstract] OR Togo[Title/Abstract] OR Uganda[Title/Abstract] OR Yemen[Title/Abstract] OR Republic of Yemen[Title/Abstract] OR Yemen Arab Republic[Title/Abstract]))	

We conducted a comprehensive search for grey literature from four sources. First, we searched the WHO Country Planning Cycle Database [19], WHO Non-communicable Diseases Document Repository [20], and WHO MiNDbank database [21] to identify countries’ most recent National Health Policies, Strategies, and Plans (NHPSPs), National Health Service Packages, National Non-communicable Disease Strategic Plans, and National Oral Health Strategic Plans. A NHPSP outlines a country’s political and resource commitment to ensuring the health of its population [22], while a National Health Service Package specifies government-defined essential public and clinical health services to be delivered at different health care levels [23]. By examining National Non-communicable Disease Strategic Plans, we assessed the extent to which countries align with WHO’s recommendations on recognizing and addressing oral health issues through the lens of common risk factors shared with other non-communicable diseases. Additionally, through searching National Oral Health Strategic Plans, we provided baseline information on the progress toward the international commitment of having operational national oral health policies by 2030 [15].

Second, we conducted searches to identify oral health content in national health policies beyond the four policy types mentioned above. This was done by using the WHO publication database [24] and the Google search engine with predefined strings. The Google search was applied to the websites of national health departments and WHO country profiles [25] (see details for the Google search in Web Appendix 2). Third, we searched three grey literature databases, including the PAIS Index, OECD iLibrary, and World Bank eLibrary (see details in Web Appendix 3). Both Google and grey literature database searches covered the period from January 2010 to May 2023. Fourth, we examined the websites and reports of major non-governmental organizations and foundations that provide international oral health care services, such as Global Dental Relief [26], Smiles for Everyone Foundation [27], and Global Child Dental Fund [28]. We utilized a snowball strategy to scrutinize the reference lists of searched peer-reviewed and grey literature.

### Inclusion and exclusion criteria

The inclusion criteria were as follows: (1) the setting in one or multiple of the 27 low-income countries; (2) containing any information related to the integration of oral health in national health policies, the inclusion of clinical and public oral health care services in government-defined essential health service lists, oral health care utilization rates, oral health care expenditures, and the number of oral health care professionals, and (3) full-text availability. We did not impose any language restrictions, using Google Translate for publications or literature not originally written in English. To provide policy makers with the most recent information, we replaced outdated policies of countries with their most recent versions, if available. We excluded clinical trial studies.

### Literature review and evidence extraction

For peer-reviewed publications, titles and abstracts of searched studies were imported into reference management tools (EndNote and Zotero). Duplicates were removed. All remaining titles and abstracts were uploaded to the Covidence platform (https://www.covidence.org/), where three coauthors independently screened and selected studies for full-text review. The selected full-text studies were subsequently retrieved and assigned to two groups of coauthors for data extraction, with each group consisting of two members. To ensure accuracy, the two coauthors of each group independently reviewed the same studies and cross-checked each other’s work. For grey literature, as most of it does not have a structured abstract, we were not able to remove duplicates with reference management tools. Therefore, we assigned the full text of all the searched grey literature to four groups of coauthors, with each group consisting of two members, to conduct a literature review and data extraction in duplicate.

We used a pilot-tested template in the form of Excel spreadsheets to record the extracted information. This template included fields for “title”, “author(s)”, “publication year”, “study location”, “study design”, “study aim”, “data source”, “data collected year”, and “main findings”. Discrepancies between group members on review and data extraction were discussed and addressed at team meetings. Additional details on data extraction can be found in Web Appendix 4.

### Evidence synthesis and analysis

We started with assessing a country’s integration of oral health into national health policies. We categorized the 27 low-income countries into three groups: first, “full integration”, defined as countries that have developed national oral health policies, thereby fulfilling target 1.1 of the WHO Global Oral Health Action Plan (2023–2030) related to establishing operational national oral health policies [15]; second, “partial integration”, defined as countries that lack such specific policies but aggregately mention oral health keywords at least five times across other national health policies; and third, “no integration”, defined as countries that lack national oral health policies and aggregately mention oral health keywords less than five times across other national health policies. In order to identify countries as “partial integration” and “no integration”, we followed previous studies [29, 30] and created a list of oral health keywords through a review of significant literature on oral health. We conducted searches for each keyword and counted the frequency of their appearance in each included national health policy document. We only counted when a keyword appeared in the context of oral health. For example, the keyword “abscess” will be counted if it pertains to dental abscesses but will not be counted if it refers to abscesses within other tissues of the body (see keywords in Web Appendix 5). The threshold of at least five mentions was established as a significant number in previous studies that assessed the policy inclusion of surgery [29, 30]. Since countries’ NHPSPs and National Non-communicable Disease Strategic Plans are the main policies demonstrating countries’ political commitments to health, we also offered a more detailed summary of oral health content within these two policy types, which is presented in the Appendix.

In terms of assessing oral health services included in countries’ essential service packages, we focused on the national health policies that include a list of government-defined essential clinical and public health services, such as National Health Service Packages, and summarized the covered oral health care services. This analysis aimed to provide insights into the extent to which oral health has received practical priority within essential healthcare coverages outlined in national policies.

To assess oral health care utilization rates, we narratively summarized utilization rates found in both peer-reviewed publications and grey literature. Additionally, we summarized the primary reasons for seeking oral health care services and the most frequently administered interventions. We also summarized the determinants and inequalities of oral health care utilizations.

We used two international data sources to assess oral health expenditures: (1) the WHO Global Oral Health Status country profiles [31], which documented per capita expenditures on outpatient dental care in 2019, aggregated from both public and private sources, and (2) the WHO Global Health Expenditure Database [32], which recorded dental outpatient expenditures per capita financed by domestic public, private, and external sources between 2016 and 2020. We downloaded data from these two sources in May 2023. We identified thirteen low-income countries with available data from both data sources and compared their recorded values.

To assess the workforce, we obtained data on the number of dentists, dental prosthetic technicians, dental assistants and dental therapists per 10,000 population from the WHO National Health Workforce Account [33]. We downloaded data for the years spanning from 2010 and 2021. Countries varied in terms of years with available data. Using countries’ most recent data, we calculated the average densities of dentists, dental prosthetic technicians, dental assistants and dental therapists in low-income countries and compared them with those in middle-income and high-income countries. We downloaded the workforce data in July 2023.

Web Appendix 6 presents a summary of the information sources used to gather evidence for analyzing each of the five research questions.

## Results

**Fig. 1:**
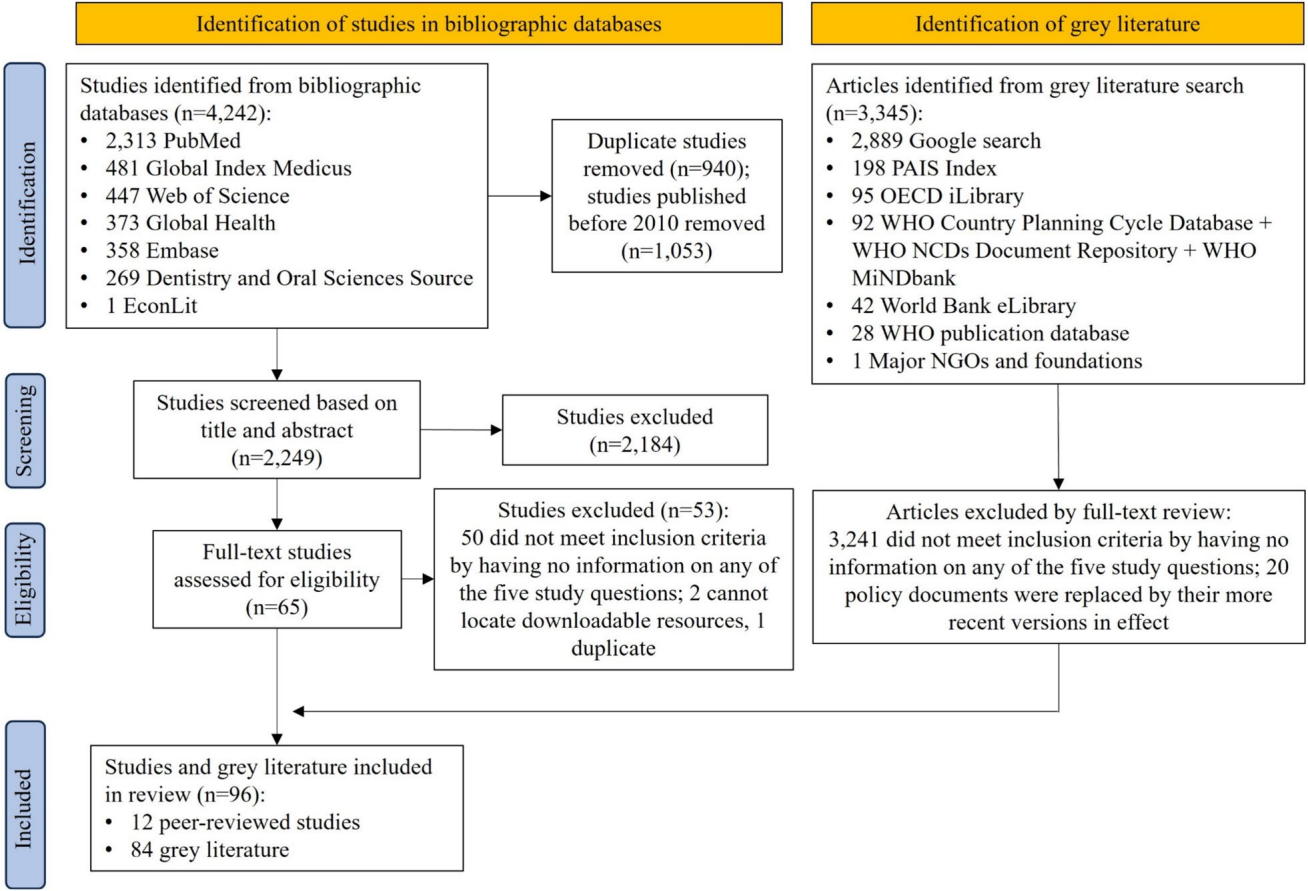
PRISMA flowchart

As shown in the PRISMA flowchart (Fig. 1), our systematic search of the seven electronic bibliographic databases yielded a total of 4242 studies. After removing duplicates, studies published before 2010, and those deemed irrelevant based on title and abstract screening, a full-text review for eligibility was given to 65 studies. From this full-text review, we identified twelve peer-reviewed papers that met our inclusion criteria. For grey literature, we obtained 3345 records from multiple sources. After a full-text review of each literature, we excluded 3241 records that did not meet the inclusion criteria, as well as 20 outdated policies that had been replaced by their more recent versions. Consequently, we included 84 pieces of grey literature. Together with the peer-reviewed studies, our scoping review ultimately included 96 articles.

### Characteristics of the 96 included articles

**Fig. 2:**
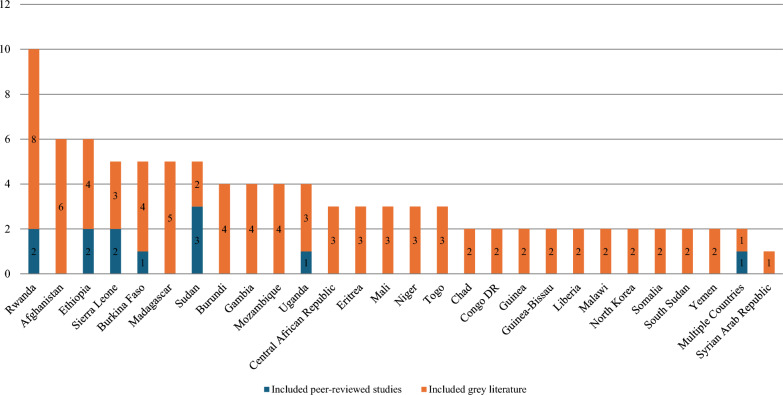
Distribution of the 96 included articles across countries

Figure 2 illustrates the distribution of the 96 included articles across countries. Notably, Rwanda stands out with the highest number of included articles at ten, followed closely by Afghanistan and Ethiopia at six. The remaining countries generally have articles ranging from one to five. We included one peer-reviewed article and one grey literature (the WHO Global Oral Health Status Report) that examined oral health resources in multiple low-income countries.

**Fig. 3:**
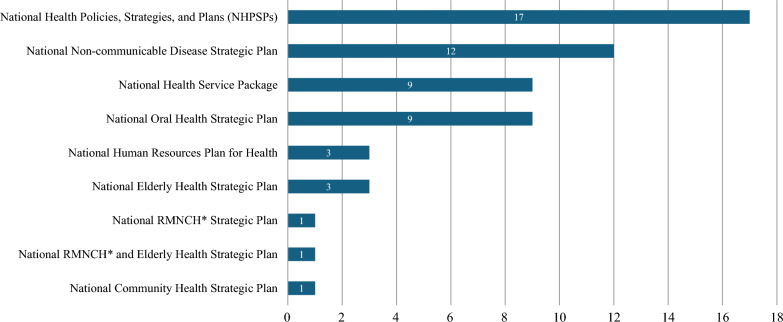
Distribution of the 56 included policy documents over policy types. *RMNCH: Reproductive, Maternal, Newborn and Child Health

For the twelve peer-reviewed papers, Table 3 demonstrates that nine out of them are questionnaire- and screening-based research that provided information on oral health care utilization, while three discussed the current stock or the development of oral health professionals. Out of the 84 pieces of grey literature, 28 consist of the WHO Global Oral Health Status Report and its associated country profiles, while the remaining 56 comprise the most recent national health policies with oral health care information. Figure 3 illustrates that the 56 national health policies belong to nine policy types, with NHPSPs being the most frequent type, followed by the National Non-communicable Disease Strategic Plan, National Health Service Package, National Oral Health Strategic Plan, and other national health policies. Appendix 7 summarizes oral health information for each of the 56 national health policies.

**Table 3:** Summary of the 12 included peer-reviewed studies

Author, year	Study location	Study period	Study aim	Study design	Data collection/participants	Main theme	Key findings
Diendéré et al. [91]	Burkina Faso	2013	To describe oral hygiene practices and associated sociodemographic factors in the Burkinabè population	Descriptive, cross-sectional	Face-to-face interview and direct measure of a nationally representative sample of 4,677 adults	Utilization	•Only 2.1% (95% CI 1.7–2.6) of the survey participants visited a dentist in the past 6 months.
Pengpid et al. [92]	Sudan	2016	To investigate the prevalence and associated factors of dental service utilization	Population-based cross-sectional	Self-reported questionnaire, anthropometric and biochemical measures were given to a nationally representative sample of 7,722 adults	Utilization	•64.6% of the participants never used dental care; 22.0% had more than 12-month use, and 13.4% had past 12- month use. •Among those who had ever used, the main reason for the last use was pain or trouble with teeth, gums, or mouth (66.9%), treatment or follow-up treatment (22.3%), and routine check-up treatment (5.0%).
Morgan et al. [9]	Rwanda	2016	To implement the first National Oral Health Survey of Rwanda to assess the oral disease burden and inform oral health promotion strategies	Descriptive, cross-sectional	Interviewer-administered questionnaire and oral health epidemiologic screening to a nationally representative sample of 2,097 participants	Utilization	•70.6% of the survey participants never visited an oral health provider for treatment. •98.7% of those who visited a dental practitioner sought care for pain relief instead of preventive, restorative or non-emergent conditions. •Of those who responded to the question why they were unable to access care, 60.3% reported that cost was the major reason for not receiving care despite nearly 70% of the population reportedly having insurance coverage that includes dental services. •The challenging factors to accessing oral care include geographical terrain, climate, the scarcity and distribution of oral health workforce, and the lack of oral care infrastructure and promotion programs.
Ghotane et al. [93]	Sierra Leone	2017	To investigate the oral health needs of school children at key ages	National survey	Self- and parent-completed questionnaire and clinic examination to a nationally representative sample of 1,174 school children at 6-, 12-, and 15-years	Utilization	•Most 12- (66%) and 15-year-olds (73%) reported ‘never having been to a dentist’, with only 8% having attended a check-up in both age-groups. •Attendance amongst 6-year-olds was lower, with only 3% of parents reporting their child had ever visited a dentist, and visited only when they had a problem.
Bassa et al. [94]	Areka town, Ethiopia	2020	To determine the relation of dental caries with nutritional status among school-age children at resource limited setting of southern Ethiopia	Community-based cross-sectional	Face-to-face interviewer-administered questionnaire and clinical assessment to 761 randomly selected school-age children (6–12 years old)	Utilization	•92.6% of survey participants have never visited a dental clinic during the past year; among those who have visited, the majority went for emergency dental pain treatment (41.4%) and extraction (8.6%).
Mohammed et al. [95]	Mekelle city, Ethiopia	2016	To determine the dental service utilization and associated factors among school-aged children (6–15) years	School-based cross-sectional	Interviews and self-administered questionnaire to the parents of a multi-stage sampling of 398 school children (6–15 years old)	Utilization	•The overall dental service utilization among the surveyed children was 10.6% (95% CI 7.5–13.6) in the past year, and 89.4% of the children had never seen a dentist. •The major reason (80.9%) for a dental visit is dental pain. •There is no dental insurance covering the associated costs for dental care targeting children and other segments of the population. •There are no school and community-based oral health programs in Ethiopia.
Salih et al. [96]	Khartoum State, Sudan	N/A	To assess the oral health status, prosthetic needs and the associated factors among older adults living in Khartoum State, Sudan	Health-Care-Center-based cross-sectional	Interview and clinical examination to 249 patients, co-patients and employees who were ≥ 60 years in the 14 selected primary healthcare centers	Utilization	•7.6% participants never received dental treatment, 37% had a dental visit within one year or less, and 55.4% had a dental visit more than one year before. •The major reason (72.6%) for last dental visit is pain or trouble with teeth, gums, or mouth.
Khalifa et al. [97]	Seven provinces of the Khartoum State, Sudan	2009–2010	To assess the oral health status and risk factors for dental caries and periodontal disease among Sudanese adult residents in Khartoum State	Descriptive, cross-sectional	Interviews and clinical examinations were conducted on a stratified sampling of 1,888 Sudanese aged ≥ 16 years from public dental hospitals and dental health centers	Utilization	•Over sixty percent of subjects went to the dentist less frequently than every 2 years, 16.7% went more frequently than every 2 years, and 22.7% never went, indicating poor attendance. •Only 9% went for regular checkups whereas > 91% of patients only went to the dentist when they were in pain. •For the most recent dental visit, more than 55% of people had a single tooth extraction as their only treatment, and nearly 80% of them stated that this was the advice given by the dentist. •Apparent lack of restorative or preventive dental care, and treatment is limited to pain relief or emergency care by tooth extraction. •The lack of public funding for oral healthcare and dental insurance schemes influences dental attendance in Sudan. •There is a lack of restorative treatment due to prohibitively high cost.
Ocwia et al. [98]	Nebbi District, Uganda	2020	To assess awareness, utilization and barriers to seeking oral health care among adults in Nebbi District, Uganda	Community-based cross-sectional	Interviewer-administered semi-structured questionnaire was used to collect data from a random sampling of 400 adults aged ≥ 18	Utilization	•Of the 51.5% who had experienced a toothache or discomfort 12 months prior to the study, only about half (52%) had sought healthcare from a dental clinic or facility. •The major reason (86.7%) for seeking dental care was toothache. •Dental caries (76.6%) and gum bleeding (14.9%) were the most frequent treated conditions, and the majority were treated by tooth extraction (73.7%).
Binagwaho et al. [99]	Rwanda	N/A	To introduce a new partnership between the U.S. and Rwandan faculties in forging human resources in health	Report	N/A	Workforce	•In 2011 there were 122 oral health professionals in Rwanda, including dental assistants, dental therapists, and surgeons, while the target of the collaborated training program was to reach 424 in 2018.
Ghotane et al. [100]	Sierra Leone	2015, 2017	To estimate needs‐led human resources for oral health	Quantitative modeling	National epidemiological survey data, Census demographic data	Workforce	•To meet the dental care needs of the overall population, an estimated 6,147 dentists would be required to deliver conventional care; 1,413 dentists required to deliver basic surgical and preventive care; 1,028 dentists required to deliver prevention care only.
Gallagher et al. [101]	Multi-country in the African Region	2002–2019	To review the oral health workforce comprising dentists, dental assistants and dental therapists, and dental prosthetic technicians in the African Region	Descriptive	Data from WHO Global Oral Health Workforce Survey and National Health Workforce Accounts	Workforce	•The workforce density of dentists (per 10,000 population) in the African region remains very low at 0.44.s

### Integration of oral health in national health policies

We identified nine countries as “full integration” as they have established their national oral health policies, including Burkina Faso [34–36], Congo DR [37], Guinea-Bissau [38], Madagascar [39–42], Mali [43, 44], Mozambique [45–47], Niger [48, 49], Rwanda [50–56], and Uganda [57, 58]; thirteen countries as “partial integration” as they do not have such specific policies but mention oral health keywords more than five times across their other national health policies, including Afghanistan [59–63], Burundi [64–66], the Central African Republic [67, 68], Chad [69], Eritrea [70, 71], Ethiopia [72–74], Gambia [75–77], Guinea [78], Liberia [79], Malawi [80], Sierra Leone [81, 82], Somalia [83], and Togo [84, 85]; five countries as “no integration” as they do not have national oral health policies and mention oral health keywords less than five times across their other national health policies, including North Korea [86], South Sudan [87], Sudan [88], Yemen [89], and the Syrian Arab Republic. For the Syrian Arab Republic, we identified its WHO oral health country profile but did not locate any national health policies with oral health content. Our detailed analyses of the integration of oral health in NHPSPs (Appendix 8) and National Non-communicable Disease Strategic Plans (Appendix 9) show that oral health is largely overlooked in these two overarching policy types in low-income countries.

### Oral health care services included in government-defined essential health service lists

We identified thirteen countries with lists of government-defined essential clinical and public health care services. Except Burundi, all the other twelve countries mentioned the coverage of oral health care services, resulting in a collective offering of 26 different types of oral health care services (Table 4). By country, Rwanda distinguished itself by offering the most extensive array of oral services to its population (12 types) [52–54]. It is followed by Ethiopia (11 types) [73], Sierra Leone (8 types) [81], Liberia (5 types) [79], Madagascar and Mozambique (4 types, respectively) [40, 45], Afghanistan, Malawi, and Somalia (3 types, respectively) [59, 80, 83], Eritrea (2 types) [71], as well as Gambia and Sudan (1 type, respectively) [76, 88].

**Table 4:** Oral health care services included in government-defined essential health service lists

Service type	Number of countries with service offered	RWA	ETH	SLE	LBR	MDG	MOZ	AFG	MWI	SOM	ERI	GMB	SDN
Tooth extraction	7	Yes	Yes	Yes	Yes	Yes			Yes	Yes			
Dental abscess drainage	4	Yes	Yes		Yes					Yes			
Dental caries treatment	3		Yes		Yes	Yes							
Cleft lip and palate repair	3	Yes	Yes		Yes								
Dental filling	3	Yes		Yes					Yes				
Dentures and prosthesis	3	Yes		Yes		Yes							
Maxillofacial trauma treatment	3	Yes	Yes					Yes					
Oral health knowledge awareness^†^	3		Yes	Yes			Yes						
Oral tumor treatment	3		Yes	Yes		Yes							
School oral health service	3				Yes		Yes				Yes		
Dental trauma treatment	2		Yes	Yes									
Gum disease and treatment	2		Yes					Yes					
Oral emergency service	2		Yes							Yes			
Oral disease preventive service^†^*	2	Yes					Yes						
Oral surgery	2	Yes		Yes									
Salivary disease treatment	2	Yes						Yes					
Atraumatic restorative treatment	1			Yes									
Basic dental care*	1												Yes
Dental scaling^†^	1	Yes											
Fluoride use expansion^†^	1						Yes						
Oral disease screening^†^	1	Yes											
Oral health service for the elderly*	1										Yes		
Orofacial infection treatment	1		Yes										
Orthodontics for dental condition	1	Yes											
Specialized dental care services*	1											Yes	
Tooth pain management	1								Yes				
Number of provided services in each country		12	11	8	5	4	4	3	3	3	2	1	1

1. Country name abbreviations: RWA (Rwanda), ETH (Ethiopia), SLE (Sierra Leone), LBR (Liberia), MDG (Madagascar), MOZ (Mozambique), AFG (Afghanistan), MWI (Malawi),

SOM (Somalia), ERI (Eritrea), GMB (Gambia), SDN (Sudan)

*No further information on specific intervention provided

^†^Preventive oral health care service

Tooth extraction emerged as the predominant intervention, appearing in the health service lists of seven countries [40, 52, 73, 79–81, 83]. Four countries covered dental abscess drainage [52, 73, 79, 83], making this procedure the second most frequently provided intervention. In contrast, as preventive measures, services on oral health knowledge awareness [45, 73, 81], dental scaling [54], fluoride use expansion [45], and oral disease screening [53] were only covered by a small number of countries. Of particular concern, only Sierra Leone explicitly covered Atraumatic Restorative Treatment (also called Alternative Restorative Treatment) (ART) [81]. As ART has been recommended by the WHO as a cost-effective approach to address dental caries in resource-limited settings [23, 90], its rare coverage indicates a consistently insufficient supply in low-income countries. Additionally, information was limited regarding what segment of the population was covered by essential oral health care services. We identified only four countries with explicit information on the age characteristics of the covered population, including Liberia [79], Mozambique [45], and Eritrea [71], covering oral health care services for school-age children; only Eritrea and Ethiopia, however, explicitly covered oral health care services for the elderly and the detection of cleft lip and palate for newborns, respectively [71, 73].

Information was scarce regarding the cost-sharing structure of oral health care services. Among the countries that included coverage of oral health care services, only Ethiopia explicitly outlined a cost-sharing arrangement. In Ethiopia, the detection of cleft lip and palate in newborns, along with the promotion of awareness regarding proper oral hygiene practices, is provided at no cost. However, other oral health care services, such as tooth extraction, involve a cost-sharing mechanism [73]. Unfortunately, detailed information on the specifics of the cost-sharing mechanism is lacking.

### Utilization rates of oral health care service

We identified seven countries with available information on the utilization rates of oral health care services. Among countries with nationally sampled populations, the individual rate was 2.0% in Mozambique among its general population (past 12 months usage) [46], 2.1% in Burkina Faso among adults (past 6 months usage) [91], 13.4% in Sudan among adults (past 12 months usage) [92], and 29.4% in Rwanda among its general population (ever usage) [9]; Sierra Leone witnessed various utilization rates of ever usage for school children at different ages, ranging from 3.0% for the 6-years-old, 34.0% for the 12-years-old, to 27.0% for the 15-years-old [93]. On the other hand, for populations sampled at the subnational or institutional level, the estimated rates ranged from 7.4% in Ethiopia’s Areka town among school children (past 12 months usage) [94], 10.6% in Ethiopia’s Mekelle city among school children (past 12 months usage) [95], 9.0% in Sudan’s Khartoum State among aged 16 and above for regular checkups and 37% among the elderly attending primary healthcare centers (past 12 months usage) [96, 97], to 52.0% in Uganda’s Nebbi District among adults (past 12 months usage) [98].

In terms of reasons for seeking oral health care services, a significant proportion of the surveyed population reported dental pain, such as 66.9% in Sudan and 98.7% in Rwanda [9, 92], or reported that they would only seek care when facing oral health conditions (e.g., school children in Sierra Leone) [93]. In the realm of treatments, tooth extraction was the most frequently administered intervention for alleviating dental pain, rather than other restorative dental care procedures. Concretely, among the individuals who sought oral health care services, 73.7% of those in Uganda’s Nebbi District had tooth extraction as their sole treatment [98], while the percentage was 55.0% in Sudan’s Khartoum State [97], and 52.0% in Mozambique [46]. In Ethiopia’s Areka town, 41.4% of those who have visited dental clinics went for emergency dental pain treatment and 8.6% for tooth extraction [94]. See details in Table 3 and Web Appendix 7.

We identified three peer-reviewed papers with information on the determinants and inequalities of dental service utilization. Among the socioeconomic determinants, adults residing in urban areas, those from wealthier households, or school children born to mothers with higher educational attainment are consistently more likely to use dental care services compared to those living in rural areas, from less wealthy households, or children born to mothers with lower education levels [91, 92, 95].

### Oral health care expenditures

**Fig. 4:**
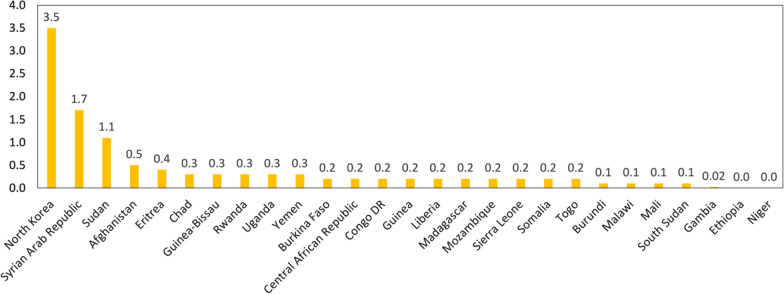
Per capita expenditures on dental outpatient care in 2019 based on Global Oral Health Status Report. Note: Currency in US dollar

**Fig. 5:**
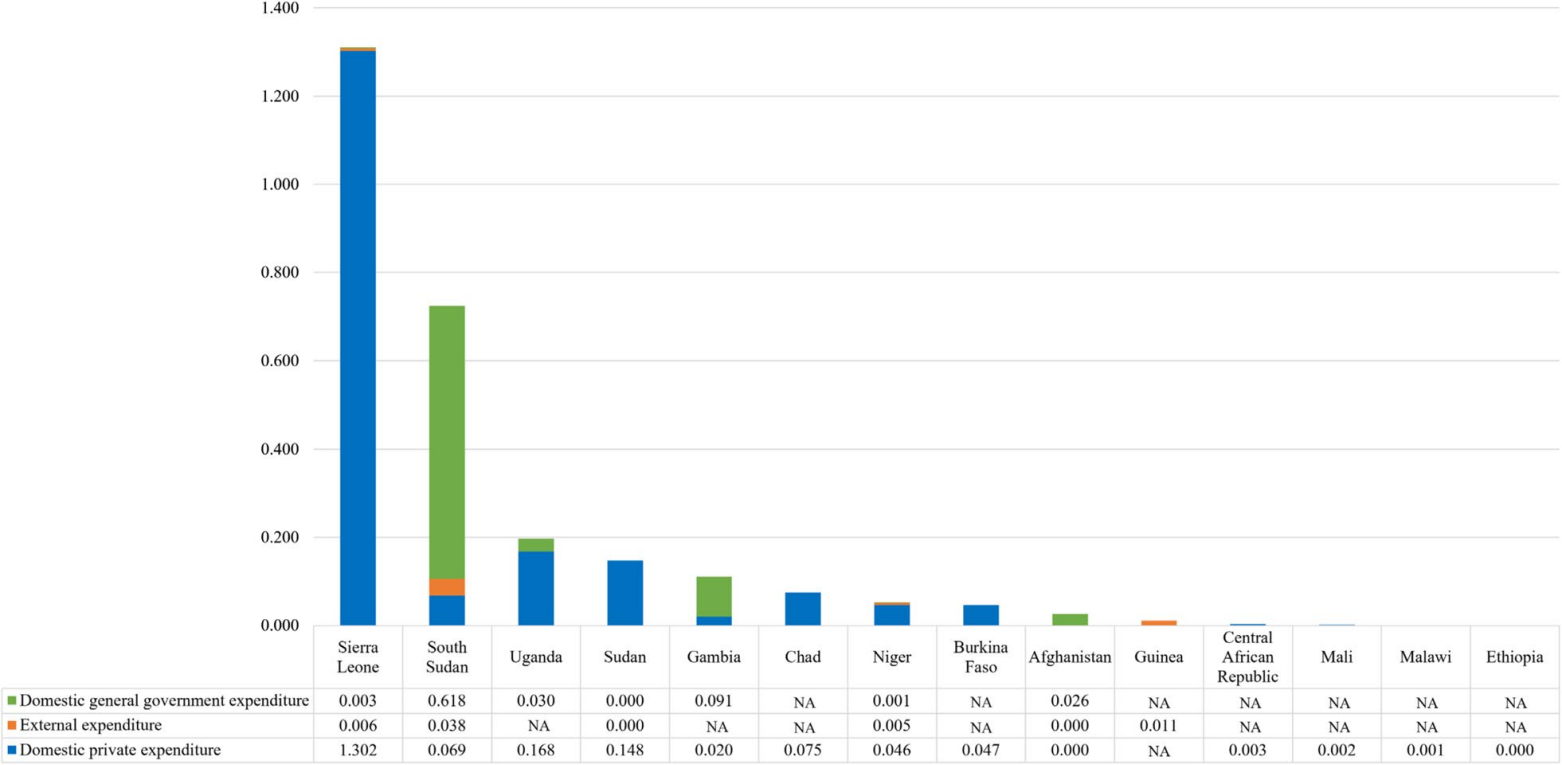
Most recent per capita expenditures on dental outpatient care based on Global Health Expenditure Database. Note: Currency in 2020 US dollar

Oral healthcare expenditures in low-income countries are low. Based on WHO’s Global Oral Health Status country profiles [31], only North Korea, the Syrian Arab Republic, and Sudan allocated more than one US dollar per capita in 2019 on dental outpatient care, while all the other countries spent an amount ranging from zero to 0.5 US dollars per capita (Fig. 4). On the other hand, according to the WHO Global Health Expenditure Database [32], fourteen countries spent an average of 0.19 US dollars per capita for dental outpatient care for the most recent year of available data. Among these fourteen countries, nine predominantly relied on the domestic private sector to bear expenditures, with only Afghanistan, Gambia, and South Sudan financing more than half of their expenditures from domestic government sources (Fig. 5).

When considering time trends, only five countries—Afghanistan, Burkina Faso, Gambia, Niger, and South Sudan—exhibited an upward trend in government expenditures from 2016 to 2020, while Sierra Leone, Sudan, and Uganda reduced their government funds during the same period. Out of the eight countries that received external funds, four experienced a decline in annual receipts. These countries are Afghanistan, Guinea, Sierra Leone, and South Sudan. Interestingly, these reductions did not lead to significant decreases in the overall expenditures on dental outpatient care in Afghanistan and South Sudan, attributed to the increased financial support from their respective domestic governments (Web Appendix 10).

Furthermore, we observed significant discrepancies in the recorded per capita expenditures on dental outpatient care in 2019 between the two data sources. These disparities are particularly notable for countries such as Afghanistan, Chad, Sierra Leone, South Sudan, and Sudan. Although the WHO Global Health Expenditure Database includes expenditures financed by external sources as an additional component, the magnitude of such expenditures is much smaller compared to the disparities. For instance, in 2019, the disparity for Serra Leone was 1.11 US dollars per capita, whereas the external expenditure was only 0.01 US dollars per capita (Web Appendix 11).

### Number of oral health professionals

Our literature review identified three papers highlighting a significant shortage of oral health professionals in low-income countries [99–101]. For the WHO National Health Workforce Account [33], from 2010 to 2021, twenty-six low-income countries reported workforce data on dentists, while only twelve countries reported data on dental prosthetic technicians, and sixteen countries reported data on dental assistants and dental therapists. Somalia is the only country that did not report any oral health workforce data.

**Fig. 6:**
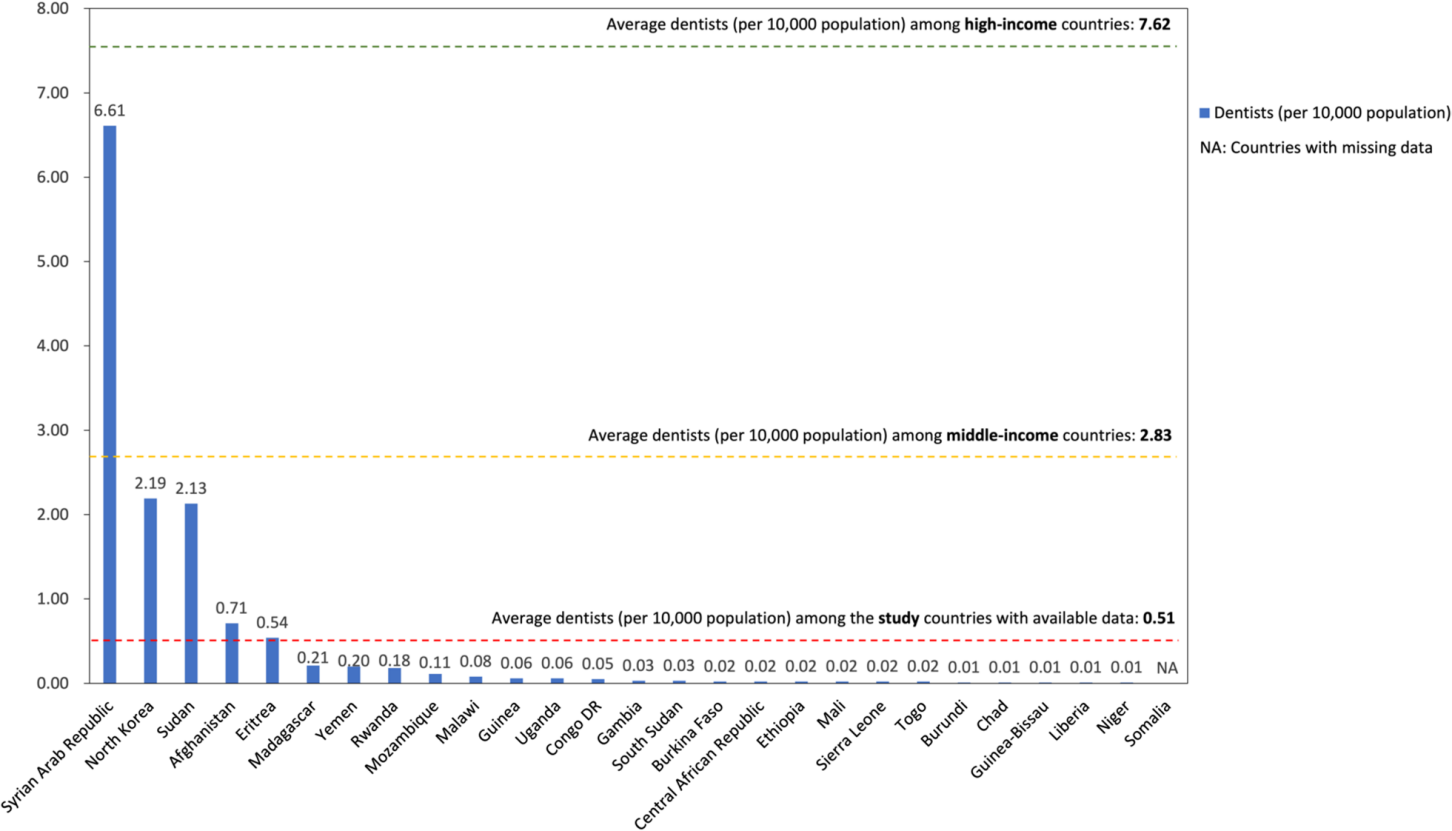
Most recent number of dentists per 10,000 population in 27 low-income countries. (Data source: WHO National Health Workforce Accounts Data Portal)

On average, the most recent dentist density is remarkably low, standing at 0.51 per 10,000 population across low-income countries (Fig. 6). This number is less than one-fifth of the density in middle-income countries (2.83) and represents a mere one-fifteenth of the density in high-income countries (7.62) [33]. By individual country, only five low-income countries exceeded the average line of 0.51. These countries are the Syrian Arab Republic (6.61), North Korea (2.19), Sudan (2.13), Afghanistan (0.71), and Eritrea (0.54). The remaining countries exhibited considerably low dentist densities, ranging from 0.01 to 0.21 dentists per 10,000 population.

**Fig. 7:**
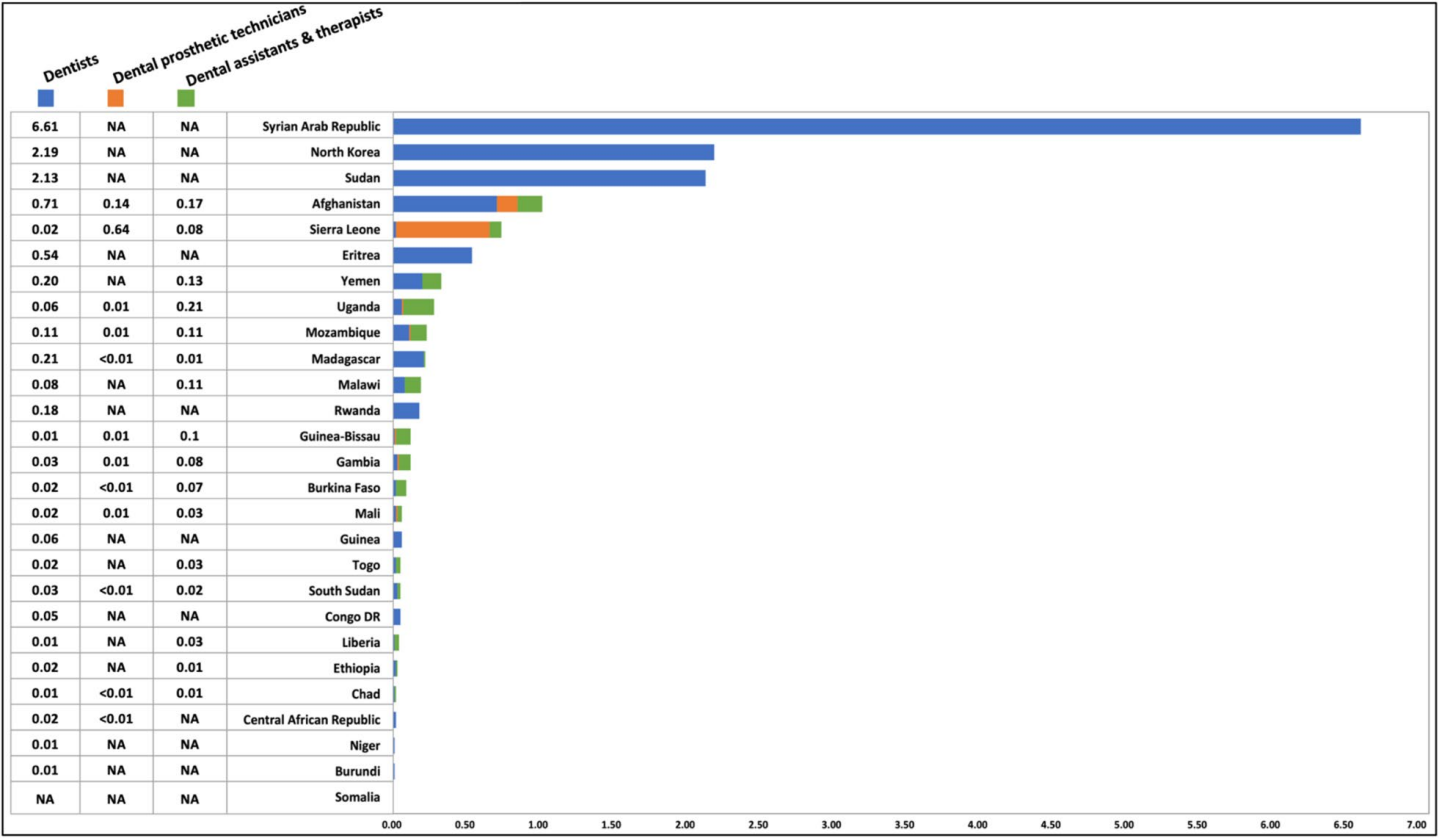
Most recent number of dentists, dental prosthetic technicians, and dental assistants and therapists per 10,000 population in 27 low-income countries. (Data source: WHO National Health Workforce Accounts Data Portal)

We also observed that some countries relied on other types of professionals, encompassing dental prosthetic technicians, dental assistants, and dental therapists, rather than predominantly on dentists to deliver oral health care services. For example, Sierra Leone’s workforce is primarily composed of dental prosthetic technicians. In countries such as Uganda, Malawi, Guinea-Bissau, Gambia, Burkina Faso, Mali, Togo, and Liberia, there is a higher prevalence of dental assistants and dental therapists compared to dentists (Fig. 7). However, the average density of other dental professionals in low-income countries remained remarkably low, with only 0.07 dental prosthetic technicians and 0.08 dental assistants and dental therapists per 10,000 population, compared to 0.46 and 0.83 in middle-income countries and 2.11 and 5.88 in high-income countries.

We grouped findings of the five study aspects by country in Web Appendix 12. For oral health expenditures, we summarized countries’ most recent data on per capita expenditures on oral outpatient care in Web Appendix 12.

## Discussion

Given the rising prevalence of oral diseases worldwide and the recent international commitment to achieving universal coverage for oral health by 2030, our study offers timely information on the universal coverage states for oral health in 27 low-income countries. Overall, we identified that there are limited sources of information on oral health in low-income countries, highlighting an urgent need to intensify efforts in progress monitoring and strengthening oral health care systems.

The extent of integrating oral health in national health policies varies across the 27 low-income countries. Notably, only nine countries have developed national oral health policies; thirteen countries lack such specific policies but have significantly incorporated oral health within their other national health policies. However, as a particular concern, five countries lack both national oral health policies and an adequate mention of oral health within their other national health policies, indicating neglect of political commitment to oral health in these nations. Furthermore, our study revealed that only five low-income countries (Afghanistan, Burkina Faso, Eritrea, Gambia, and Madagascar) have explicitly included oral health in national health policies designed for maternal, children, and elderly health [35, 42, 62, 71, 75]. This small number reflects that the concept of treating oral disease as a progressive condition throughout the life course received little attention from policy makers in low-income countries.

In addition, we identified a large deficiency in oral health service provision in low-income countries. According to WHO, the African region faces a substantial disease burden from seven oral diseases, including dental caries, periodontal diseases, oral cancers, noma, oral manifestation of HIV and AIDS, orofacial trauma, and cleft lip and palate [23]. Accordingly, the WHO recommended the inclusion of three key interventions—oral urgent treatment, affordable fluoride toothpaste, and atraumatic restorative treatment—in the basic package of oral care for resource-limited settings [90]. However, we found that only four low-income countries explicitly included some of these three key interventions in their government-defined essential health care service lists, and no low-income countries listed noma and the management of the oral manifestation of HIV and AIDS. In contrast, we observed a skewed prioritization toward invasive clinic procedures, such as tooth extraction and dental abscess drainage, rather than preventive interventions delivered in a public health approach, for example, the usage of affordable fluoride toothpaste. This finding reconfirmed the deep-seated Westernized model of dentistry in low-income countries, which relied on a clinical interventionist philosophy but oversight addressing oral health issues in an upstream and community-based approach [1].

An example of an upstream public health approach that we identified is the integration of fluoride usage and school interventions in Mozambique. In its Health Sector Strategic Plan 2014–2019 [45], Mozambique prioritized access to fluorides through the widespread use of fluoride toothpaste, water fluoridation, and fluoride-based mouthwash for children aged 6–12. Additionally, in its National Oral Health Strategy 2019–2024 [46], the country advanced oral health education and daily interventions such as tooth brushing in schools. These public health strategies hold the potential to enhance the accessibility and affordability of effective upstream interventions, empower individuals with self-care skills for oral hygiene, and cultivate health behaviors that favor oral health [15]. We advocate for low-income countries to share experiences on advancing oral health prevention, promotion, and management services, and to implement the WHO Global Oral Health Action Plan 2023–2030 according to their national priorities and context. This approach mirrors the efforts of South-East Asian countries, as highlighted in the Report for the Regional Meeting for Implementing the Action Plan for Oral Health 2022–2023, where countries emphasized interactions and experience sharing on oral health systems across the region [102].

In addition, our study indicates that data on oral health care utilization, oral health care expenditure, and workforce are very limited in low-income countries, reflecting countries’ low level of investment in progress monitoring and system strengthening for oral health. For example, we only observed three countries (Afghanistan, Burundi, and Rwanda) that included the development of oral health professionals in their national health human resource plans [56, 61, 64]. Furthermore, despite the evidence that out-of-pocket expenditures for oral health care are among the main drivers of catastrophic health expenditures [10, 11], most low-income countries with available expenditure data still require a dominant proportion of payments to be borne by the domestic private sector. Considering that most low-income countries simultaneously lack a provision for WHO-recommended interventions, our study unveiled a worrisome situation of spending valuable out-of-pocket resources on the less-effective and sometimes harmful measures, like the removal of the uvula and dental buds for children [103]. Relying on traditional oral health interventions has been demonstrated as not financially sustainable in low-income countries [104], and we call for policy makers to reconfigure the oral health system and better reorientate public and private financial resources toward recommended evidence-based interventions. Furthermore, the discrepancies in expenditure data between international data sources underscore the need for increased efforts within the international community to coordinate endeavors in data collection. Such coordination is essential in providing policy makers with aligned and reliable measurement results, thereby supporting evidence-based policymaking when allocating financial resources to oral health.

Our study has several limitations. First, when searching for national policies containing oral health information, we used the websites of countries’ health departments and WHO’s existing databases. This approach may overlook oral health information contained in policies released by other departments, such as the education departments. Second, we employed public-facing websites and databases to download national health policy documents. This approach does not eliminate the possibility that oral health policies might be outlined in government internal documents that are not publicly accessible. Additionally, there remains the chance that updated versions of the accessible documents exist, but their existence is unknown to us. For future analyses, we recommend using personal contact to identify dedicated government staff responsible for oral health to locate relevant policy documents.

## Conclusions

Our study provides a comprehensive examination of five aspects of universal health coverage for oral health care in low-income countries. Policy integration for oral health care significantly varied across the 27 low-income countries while most countries do not have dedicated national oral health policies and lack services that tackle oral health within the framework of common risk factors with non-communicable diseases. Tooth extraction and other invasive interventions are dominant in low-income countries, with only a few countries adhering to WHO recommendations on the provision of preventive and public health measures for oral health, which is concerning. Financial and human resources are scarce in low-income countries, and more political and resource commitment is needed to reverse the low utilization rates in low-income countries.

## Supplementary Information

Supplementary Material 1

## Data Availability

All data relevant to the study are included in the article or uploaded as supplementary information.

## Abbreviations

LMICs: Low- and Middle-Income Countries
NHPSPs: National Health Policies, Strategies, and Plans
UHC: Universal Health Coverage
WHO: World Health Organization
WHA: World Health Assembly
