# Supplementary material for: Universal coverage for oral health care in 27 low-income countries: a scoping review
Source: Glob Health Res Policy. 2024 Sep 10;9:34. doi: 10.1186/s41256-024-00376-9 (PMC11384684; doi:10.1186/s41256-024-00376-9)
Supplement: Supplementary file 1 — Supplementary Material 1 [file 41256_2024_376_MOESM1_ESM.docx]

**Universal coverage for oral health care in 27 low-income countries: a scoping review**

**Web appendix**

**Web Appendix 1. Search string for seven bibliographic databases**

| **1. PubMed search string:**  (("Dental Health Services"[Mesh] OR "Oral Health"[Mesh] OR "Dental Facilities"[Mesh]) OR (((dental[Title/Abstract] OR oral[Title/Abstract] OR teeth[Title/Abstract] OR tooth[Title/Abstract]) AND (care[Title/Abstract] OR health[Title/Abstract] OR service[Title/Abstract] OR access*[Title/Abstract] OR disparit*[Title/Abstract] OR coverage[Title/Abstract] OR insurance[Title/Abstract] OR utilization[Title/Abstract] OR spending[Title/Abstract] OR expenditure[Title/Abstract] OR financing[Title/Abstract] OR package[Title/Abstract] OR scheme[Title/Abstract] OR plan[Title/Abstract] OR strateg*[Title/Abstract] OR utilization[Title/Abstract])) OR dental facility[Title/Abstract] OR dental facilities[Title/Abstract] OR dental office[Title/Abstract] OR dental offices[Title/Abstract])) AND (((((((((((((((((((((((((((("Afghanistan"[Mesh]) OR "Burundi"[Mesh]) OR "Burkina Faso"[Mesh]) OR "Central African Republic"[Mesh]) OR "Chad"[Mesh]) OR "Democratic Republic of the Congo"[Mesh]) OR "Eritrea"[Mesh]) OR "Ethiopia"[Mesh]) OR "Gambia"[Mesh]) OR "Guinea"[Mesh]) OR "Guinea-Bissau"[Mesh]) OR "Liberia"[Mesh]) OR "Madagascar"[Mesh]) OR "Mali"[Mesh]) OR "Malawi"[Mesh]) OR "Mozambique"[Mesh]) OR "Niger"[Mesh]) OR "Democratic People's Republic of Korea"[Mesh]) OR "Rwanda"[Mesh]) OR "Sierra Leone"[Mesh]) OR "Somalia"[Mesh]) OR "Sudan"[Mesh]) OR "South Sudan"[Mesh]) OR "Syria"[Mesh]) OR "Togo"[Mesh]) OR "Uganda"[Mesh]) OR "Yemen"[Mesh]) OR (Afghanistan[Title/Abstract] OR Burundi[Title/Abstract] OR Burkina Faso[Title/Abstract] OR Central African Republic[Title/Abstract] OR Centrafrican Republic[Title/Abstract] OR Centrafique[Title/Abstract] OR Central African Empire[Title/Abstract] OR Chad[Title/Abstract] OR Congo[Title/Abstract] OR Democratic Republic of Congo[Title/Abstract] OR Eritrea[Title/Abstract] OR Ethiopia[Title/Abstract] OR Guinea[Title/Abstract] OR Gambia[Title/Abstract] OR Guinea Bissau[Title/Abstract] OR Guinea-Bissau[Title/Abstract] OR GuineaBissau[Title/Abstract] OR Liberia[Title/Abstract] OR Madagascar[Title/Abstract] OR Malagasy Republic[Title/Abstract] OR Mali[Title/Abstract] OR Malawi[Title/Abstract] OR Mozambique[Title/Abstract] OR Niger[Title/Abstract] OR North Korea[Title/Abstract] OR Democratic People's Republic of Korea[Title/Abstract] OR Korean People's Republic[Title/Abstract] OR Rwanda[Title/Abstract] OR Sierra Leone[Title/Abstract] OR Somalia[Title/Abstract] OR Sudan[Title/Abstract] OR South Sudan[Title/Abstract] OR Syria[Title/Abstract] OR Syrian Arab Republic[Title/Abstract] OR Togo[Title/Abstract] OR Uganda[Title/Abstract] OR Yemen[Title/Abstract] OR Republic of Yemen[Title/Abstract] OR Yemen Arab Republic[Title/Abstract]))  **2. Embase (Elsevier) search string:**  #8 #3 AND #6 AND [embase]/lim  #7 #3 AND #6  #6 #4 OR #5  #5 afghanistan:ti,ab,kw OR burundi:ti,ab,kw OR 'burkina faso':ti,ab,kw OR 'central african republic':ti,ab,kw OR 'centrafrican republic':ti,ab,kw OR centrafrique:ti,ab,kw OR 'central african empire':ti,ab,kw OR chad:ti,ab,kw OR congo:ti,ab,kw OR 'democratic republic of congo':ti,ab,kw OR eritrea:ti,ab,kw OR ethiopia:ti,ab,kw OR guinea:ti,ab,kw OR gambia:ti,ab,kw OR 'guinea bissau':ti,ab,kw OR guineabissau:ti,ab,kw OR liberia:ti,ab,kw OR madagascar:ti,ab,kw OR 'malagasy republic':ti,ab,kw OR mali:ti,ab,kw OR malawi:ti,ab,kw OR mozambique:ti,ab,kw OR niger:ti,ab,kw OR 'north korea':ti,ab,kw OR 'democratic people s republic of korea':ti,ab,kw OR 'korean people s republic':ti,ab,kw OR rwanda:ti,ab,kw OR 'sierra leone':ti,ab,kw OR somalia:ti,ab,kw OR sudan:ti,ab,kw OR 'south sudan':ti,ab,kw OR syria:ti,ab,kw OR 'syrian arab republic':ti,ab,kw OR togo:ti,ab,kw OR uganda:ti,ab,kw OR yemen:ti,ab,kw OR 'republic of yemen':ti,ab,kw OR 'yemen arab republic':ti,ab,kw  #4 'afghanistan'/exp OR 'burundi'/exp OR 'burkina faso'/exp OR 'central african republic'/exp OR 'Democratic Republic Congo'/exp OR 'chad'/exp OR 'eritrea'/exp OR 'ethiopia'/exp OR 'gambia'/exp OR 'guinea'/exp OR 'guinea-bissau'/exp OR 'liberia'/exp OR 'madagascar'/exp OR 'mali'/exp OR 'malawi'/exp OR 'mozambique'/exp OR 'niger'/exp OR 'north korea'/exp OR 'rwanda'/exp OR 'sierra leone'/exp OR 'somalia'/exp OR 'sudan'/exp OR 'south sudan'/exp OR 'syrian arab republic'/exp OR 'togo'/exp OR 'uganda'/exp OR 'yemen'/exp  #3 #1 OR #2  #2 'dental facility':ti,ab,kw OR 'dental facilities':ti,ab,kw OR 'dental office*':ti,ab,kw OR (((dental OR oral OR teeth OR tooth) NEAR/3 (care OR health OR service OR access* OR disparit* OR coverage OR insurance OR utilization OR spending OR expenditure OR financing OR package OR scheme OR plan OR strateg* OR utilization)):ti,ab,kw)  #1 'dental procedure'/exp OR 'dental health'/exp OR 'dental facility'/exp OR 'dental facility'/exp OR 'dental insurance'/exp  **3. Web of Science search string:**  #3 #1 AND #2  #2 TS=(Afghanistan OR Burundi OR “Burkina Faso” OR “Central African Republic” OR “Centrafrican Republic” OR centrafrique OR “Central African Empire” OR Chad OR Congo OR “Democratic Republic of Congo” OR Eritrea OR Ethiopia OR Guinea OR Gambia OR “Guinea Bissau” OR GuineaBissau OR Liberia OR Madagascar OR “Malagasy Republic” OR Mali OR Malawi OR Mozambique OR Niger OR North Korea OR “Democratic People's Republic of Korea” OR “Korean People's Republic” OR Rwanda OR “Sierra Leone” OR Somalia OR Sudan OR “South Sudan” OR Syria OR “Syrian Arab Republic” OR Togo OR Uganda OR Yemen OR “Republic of Yemen” OR “Yemen Arab Republic”)  #1 TS=(“dental facility” OR “dental facilities” OR “dental office” OR “dental offices” OR ((dental OR oral OR teeth OR tooth) NEAR/3 (care OR health OR service OR access* OR disparit* OR coverage OR insurance OR utilization OR spending OR expenditure OR financing OR package OR scheme OR plan OR strateg* OR utilization)))  **4. EconLit search string:**  #3 #1 AND #2  #2 ab(Afghanistan OR Burundi OR “Burkina Faso” OR “Central African Republic” OR “Centrafrican Republic” OR Centrafique OR “Central African Empire” OR Chad OR “Democratic Republic of Congo” OR “Congo Democratic Republic” OR Eritrea OR Ethiopia OR Guinea OR Gambia OR “Guinea Bissau” OR Guinea-Bissau OR GuineaBissau OR Liberia OR Madagascar OR “Malagasy Republic” OR Mali OR Malawi OR Mozambique OR Niger OR “North Korea” OR “Democratic People's Republic of Korea” OR “Korean People's Republic” OR Rwanda OR “Sierra Leone” OR Somalia OR Sudan OR “South Sudan” OR Syria OR “Syrian Arab Republic” OR Togo OR Uganda OR Yemen OR “Republic of Yemen” OR “Yemen Arab Republic”) OR ti(Afghanistan OR Burundi OR “Burkina Faso” OR “Central African Republic” OR “Centrafrican Republic” OR Centrafique OR “Central African Empire” OR Chad OR “Democratic Republic of Congo” OR “Congo Democratic Republic” OR Eritrea OR Ethiopia OR Guinea OR Gambia OR “Guinea Bissau” OR Guinea-Bissau OR GuineaBissau OR Liberia OR Madagascar OR “Malagasy Republic” OR Mali OR Malawi OR Mozambique OR Niger OR “North Korea” OR “Democratic People's Republic of Korea” OR “Korean People's Republic” OR Rwanda OR “Sierra Leone” OR Somalia OR Sudan OR “South Sudan” OR Syria OR “Syrian Arab Republic” OR Togo OR Uganda OR Yemen OR “Republic of Yemen” OR “Yemen Arab Republic”)  #1 ab(“dental facility” OR “dental facilities” OR “dental office*” OR ((dental OR oral OR teeth OR tooth) NEAR/3 (care OR health OR service OR access* OR disparit* OR coverage OR insurance OR utilization OR spending OR expenditure OR financing OR package OR scheme OR plan OR strateg* OR utilization))) OR ti(“dental facility” OR “dental facilities” OR “dental office*” OR ((dental OR oral OR teeth OR tooth) NEAR/3 (care OR health OR service OR access* OR disparit* OR coverage OR insurance OR utilization OR spending OR expenditure OR financing OR package OR scheme OR plan OR strateg* OR utilization)))  **5. Global Health (EBSCO) search string:**  #7 #3 AND #6  #6 #4 OR #5  #5 TI ( Afghanistan OR Burundi OR “Burkina Faso” OR “Central African Republic” OR “Centrafrican Republic” OR Centrafique OR “Central African Empire” OR Chad OR “Democratic Republic of Congo” OR “Congo Democratic Republic” OR Eritrea OR Ethiopia OR Guinea OR Gambia OR “Guinea Bissau” OR Guinea-Bissau OR GuineaBissau OR Liberia OR Madagascar OR “Malagasy Republic” OR Mali OR Malawi OR Mozambique OR Niger OR “North Korea” OR “Democratic People's Republic of Korea” OR “Korean People's Republic” OR Rwanda OR “Sierra Leone” OR Somalia OR Sudan OR “South Sudan” OR Syria OR “Syrian Arab Republic” OR Togo OR Uganda OR Yemen OR “Republic of Yemen” OR “Yemen Arab Republic” ) OR AB ( Afghanistan OR Burundi OR “Burkina Faso” OR “Central African Republic” OR “Centrafrican Republic” OR Centrafique OR “Central African Empire” OR Chad OR “Democratic Republic of Congo” OR “Congo Democratic Republic” OR Eritrea OR Ethiopia OR Guinea OR Gambia OR “Guinea Bissau” OR Guinea-Bissau OR GuineaBissau OR Liberia OR Madagascar OR “Malagasy Republic” OR Mali OR Malawi OR Mozambique OR Niger OR “North Korea” OR “Democratic People's Republic of Korea” OR “Korean People's Republic” OR Rwanda OR “Sierra Leone” OR Somalia OR Sudan OR “South Sudan” OR Syria OR “Syrian Arab Republic” OR Togo OR Uganda OR Yemen OR “Republic of Yemen” OR “Yemen Arab Republic” )  #4 (((((((((((((((((((((((((DE "Afghanistan") OR (DE "Burundi")) OR (DE "Burkina Faso")) OR (DE "Central African Republic")) OR (DE "Chad")) OR (DE "Congo Democratic Republic")) OR (DE "Eritrea")) OR (DE "Ethiopia")) OR (DE "Gambia")) OR (DE "Guinea")) OR (DE "Guinea-Bissau")) OR (DE "Liberia")) OR (DE "Madagascar")) OR (DE "Mali")) OR (DE "Malawi")) OR (DE "Mozambique")) OR (DE "Niger")) OR (DE "Korea Democratic People's Republic")) OR (DE "Rwanda")) OR (DE "Sierra Leone")) OR (DE "Somalia")) OR (DE "Sudan")) OR (DE "Syria")) OR (DE "Togo")) OR (DE "Uganda")) OR (DE "Yemen")  #3 #1 OR #2  #2 TI ( “dental facility” OR “dental facilities” OR “dental office” OR “dental offices” OR ((dental OR oral OR teeth OR tooth) N3 (care OR health OR service OR access* OR disparit* OR coverage OR insurance OR utilization OR spending OR expenditure OR financing OR package OR scheme OR plan OR strateg* OR utilization)) ) OR AB ( “dental facility” OR “dental facilities” OR “dental office” OR “dental offices” OR ((dental OR oral OR teeth OR tooth) N3 (care OR health OR service OR access* OR disparit* OR coverage OR insurance OR utilization OR spending OR expenditure OR financing OR package OR scheme OR plan OR strateg* OR utilization)) )  #1 DE “dental health” OR DE “dentistry” OR DE “teeth” OR DE “tooth diseases”  **6. Global Index Medicus search string:**  (tw:(((dental OR oral OR teeth OR tooth) AND (care OR health OR service OR access* OR disparit* OR coverage OR insurance OR utilization OR spending OR expenditure OR financing OR package OR scheme OR plan OR strateg* OR utilization)) OR "dental facilities" OR "dental facility" OR "dental office")) AND (tw:(Afghanistan OR Burundi OR "Burkina Faso" OR "Central African Republic" OR "Centrafrican Republic" OR Centrafique OR "Central African Empire" OR Chad OR Congo OR "Democratic Republic of Congo" OR Eritrea OR Ethiopia OR Guinea OR Gambia OR "Guinea Bissau" OR GuineaBissau OR Liberia OR Madagascar OR "Malagasy Republic" OR Mali OR Malawi OR Mozambique OR Niger OR "North Korea" OR "Democratic People s Republic of Korea" OR "Korean People s Republic" OR Rwanda OR "Sierra Leone" OR Somalia OR Sudan OR Syria OR "Syrian Arab Republic" OR Togo OR Uganda OR Yemen))  **7. Dentistry and Oral Sciences Source (EBSCO):**  #5 #3 AND #4  #4 TI ( Afghanistan OR Burundi OR “Burkina Faso” OR “Central African Republic” OR “Centrafrican Republic” OR Centrafique OR “Central African Empire” OR Chad OR “Democratic Republic of Congo” OR “Congo Democratic Republic” OR Eritrea OR Ethiopia OR Guinea OR Gambia OR “Guinea Bissau” OR Guinea-Bissau OR GuineaBissau OR Liberia OR Madagascar OR “Malagasy Republic” OR Mali OR Malawi OR Mozambique OR Niger OR “North Korea” OR “Democratic People's Republic of Korea” OR “Korean People's Republic” OR Rwanda OR “Sierra Leone” OR Somalia OR Sudan OR “South Sudan” OR Syria OR “Syrian Arab Republic” OR Togo OR Uganda OR Yemen OR “Republic of Yemen” OR “Yemen Arab Republic” ) OR AB ( Afghanistan OR Burundi OR “Burkina Faso” OR “Central African Republic” OR “Centrafrican Republic” OR Centrafique OR “Central African Empire” OR Chad OR “Democratic Republic of Congo” OR “Congo Democratic Republic” OR Eritrea OR Ethiopia OR Guinea OR Gambia OR “Guinea Bissau” OR Guinea-Bissau OR GuineaBissau OR Liberia OR Madagascar OR “Malagasy Republic” OR Mali OR Malawi OR Mozambique OR Niger OR “North Korea” OR “Democratic People's Republic of Korea” OR “Korean People's Republic” OR Rwanda OR “Sierra Leone” OR Somalia OR Sudan OR “South Sudan” OR Syria OR “Syrian Arab Republic” OR Togo OR Uganda OR Yemen OR “Republic of Yemen” OR “Yemen Arab Republic” ) OR KW ( Afghanistan OR Burundi OR “Burkina Faso” OR “Central African Republic” OR “Centrafrican Republic” OR Centrafique OR “Central African Empire” OR Chad OR “Democratic Republic of Congo” OR “Congo Democratic Republic” OR Eritrea OR Ethiopia OR Guinea OR Gambia OR “Guinea Bissau” OR Guinea-Bissau OR GuineaBissau OR Liberia OR Madagascar OR “Malagasy Republic” OR Mali OR Malawi OR Mozambique OR Niger OR “North Korea” OR “Democratic People's Republic of Korea” OR “Korean People's Republic” OR Rwanda OR “Sierra Leone” OR Somalia OR Sudan OR “South Sudan” OR Syria OR “Syrian Arab Republic” OR Togo OR Uganda OR Yemen OR “Republic of Yemen” OR “Yemen Arab Republic” )  #3 #1 OR #2  #2 TI ( "dental facility” OR “dental facilities” OR “dental office” OR “dental offices” OR ((dental OR oral OR teeth OR tooth) N3 (care OR health OR service OR access* OR disparit* OR coverage OR insurance OR utilization OR spending OR expenditure OR financing OR package OR scheme OR plan OR strateg* OR utilization)) ) OR AB ( "dental facility” OR “dental facilities” OR “dental office” OR “dental offices” OR ((dental OR oral OR teeth OR tooth) N3 (care OR health OR service OR access* OR disparit* OR coverage OR insurance OR utilization OR spending OR expenditure OR financing OR package OR scheme OR plan OR strateg* OR utilization)) ) OR KW ( "dental facility” OR “dental facilities” OR “dental office” OR “dental offices” OR ((dental OR oral OR teeth OR tooth) N3 (care OR health OR service OR access* OR disparit* OR coverage OR insurance OR utilization OR spending OR expenditure OR financing OR package OR scheme OR plan OR strateg* OR utilization)) )  #1 (((((((((((((((((((((((DE "DENTAL insurance" OR DE "DENTAL health maintenance organizations" OR DE "GOVERNMENT employees' dental insurance" OR DE "MANAGED dental care") OR (DE "ORAL health")) OR (DE "NUTRITION & oral health")) OR (DE "DENTAL facilities" OR DE "DENTAL clinics" OR DE "DENTAL laboratories" OR DE "DENTAL offices")) OR (DE "DENTAL care" OR DE "COMMUNITY dental services" OR DE "DENTAL hygiene" OR DE "DENTAL tourism" OR DE "HOSPITAL dental service" OR DE "MANAGED dental care" OR DE "TOOTH care & hygiene")) OR (DE "COST of dental care" OR DE "DENTAL fees")) AND (DE "DENTISTRY" OR DE "DENTAL anesthesia" OR DE "DENTAL occlusion" OR DE "DENTAL specialties" OR DE "DENTAL technology" OR DE "DENTAL therapeutics" OR DE "ENDODONTICS" OR DE "FOLK dentistry" OR DE "HOSPITAL dental service" OR DE "OPERATIVE dentistry" OR DE "ORTHODONTICS" OR DE "PEDIATRIC dentistry" OR DE "PERIODONTICS" OR DE "PREVENTIVE dentistry" OR DE "PROSTHODONTICS")) OR (DE "ORAL diseases")) OR (DE "PUBLIC spending")) OR (DE "RURAL-urban differences")) OR (DE "STRATEGIC planning")) AND (DE "TOOTH care & hygiene" OR DE "CAVITY prevention" OR DE "DENTAL floss" OR DE "DENTAL hygiene" OR DE "REMINERALIZATION (Teeth)" OR DE "TEETH polishing" OR DE "ORAL hygiene")) OR (DE "DENTISTS")) OR (DE "DENTAL teams")) OR (DE "DENTAL economics" OR DE "COST of dental care" OR DE "GROUP dental practice")) OR (DE "ECONOMICS")) OR (DE "FINANCE")) OR (DE "HEALTH planning")) OR (DE "MEDICAL care financing")) OR (DE "HEALTH facilities utilization")) OR (DE "ORAL medicine" OR DE "EXPERIMENTAL oral medicine" OR DE "PEDIATRIC oral medicine" OR DE "VETERINARY oral medicine")) OR (DE "ORAL habits")) OR (DE "HELP-seeking behavior")) OR (DE "INSURANCE") |
| --- |

**Web Appendix 2. Details for searching national health policies with Google search engine**

We developed Google search strings to search on the websites of countries’ Department of Health and WHO country profile for national health policy documents containing oral health information. We conducted keyword searches in English, as well as in each country’s official languages, other than English, where applicable. We did not conduct a Google search for the question of the number oral health care workforce as we directly retrieved workforce data from the WHO National Health Workforce Account Database. This section presents the Google search for the other four research questions and uses Rwanda as an example for the illustrative purpose.

**1. Research question: The integration of oral health in national health policies**

- **Purpose**
  - Search and download national health policies, strategies, plans
- **Steps**
  - Identify the website addresses for each country’s Department of Health and its WHO profile webpage.
  - Modify search string based on the website addresses and each country’s official languages.
  - Execute the search string on Google and download captured documents.
- **Example: Google search string for Rwanda in English**
  - (site:moh.gov.rw OR site:who.int/countries/rwa/) after:2010-01-01 (plan OR strategy OR strategic) (filetype:pdf OR filetype:ppt OR filetype:pptx OR filetype:doc OR filetype:docx).

**2. Research question: The inclusion of clinical and public oral health care services in government-defined essential health service lists**

- **Purpose**
  - Search and download government documents containing information on clinical and public oral health care services.
- **Steps**
  - Identify the website addresses for each country’s Department of Health and its WHO profile webpage.
  - Modify search string based on the website addresses and each country’s official languages.
  - Execute the search string on Google and download captured documents.
- **Example: Google search string for Rwanda in English**
  - (site:who.int OR site:moh.gov.rw) “Rwanda” after:2010-01-01 (Mutuelles OR intitle:package OR intitle:insurance OR intitle:scheme OR intitle:coverage) (dental OR dentistry OR dentist OR “oral health” OR “oral care”) (filetype:pdf OR filetype:ppt OR filetype:pptx OR filetype:doc OR filetype:docx)

**3. Research question: Oral health care utilization rates**

- **Purpose**
  - Search and download government documents containing information on oral health care utilization rates.
- **Steps**
  - Identify the website addresses for each country’s Department of Health and its WHO profile webpage.
  - Modify search string based on the website addresses and each country’s official languages.
  - Execute the search string on Google and download captured documents.
- **Example: Google search string for Rwanda in English**
  - (site:who.int OR site:moh.gov.rw) “Rwanda” “utilization rate” after:2010-01-01 (dental OR dentistry OR dentist OR “oral health” OR “oral care”) (filetype:pdf OR filetype:ppt OR filetype:pptx OR filetype:doc OR filetype:docx)

**4. Research question: Oral health care expenditures**

- **Purpose**
  - Search and download government documents containing information on oral health care expenditures
- **Steps**
  - Identify the website addresses for each country’s Department of Health and Finance.
  - Modify search string based on the website addresses and each country’s official languages.
  - Execute the search string on Google and download captured documents.
- **Example: Google search string for Rwanda in English**
  - (site:minecofin.gov.rw OR site:moh.gov.rw) after:2010-01-01 (dental OR dentistry OR dentist OR “oral health” OR “oral care”) (finance OR financial OR financing OR expenditure OR spend OR spending OR budget) (filetype:pdf OR filetype:ppt OR filetype:pptx OR filetype:doc OR filetype:docx)

**Web Appendix 3. Search string for PAIS Index, World Bank eLibrary, and OECD iLibrary**

| **1. PAIS Index:**  Searched for: (('dental facility' OR 'dental facilities' OR 'dental office*' OR ((dental OR oral OR teeth OR tooth) NEAR/2 (care OR health OR service OR access* OR disparit* OR coverage OR insurance OR utilization OR spending OR expenditure OR financing OR package OR scheme OR plan OR strateg* OR utilisation))) OR ti('dental facility' OR 'dental facilities' OR 'dental office*' OR ((dental OR oral OR teeth OR tooth) NEAR/2 (care OR health OR service OR access* OR disparit* OR coverage OR insurance OR utilization OR spending OR expenditure OR financing OR package OR scheme OR plan OR strateg* OR utilisation)))) AND ((Afghanistan OR Burundi OR 'Burkina Faso' OR 'Central African Republic' OR 'Centrafrican Republic' OR Centrafique OR 'Central African Empire' OR Chad OR Congo OR 'Democratic Republic of Congo' OR Eritrea OR Ethiopia OR Guinea OR Gambia OR 'Guinea Bissau' OR Guinea-Bissau OR GuineaBissau OR Liberia OR Madagascar OR 'Malagasy Republic' OR Mali OR Malawi OR Mozambique OR Niger OR 'North Korea' OR 'Democratic People's Republic of Korea' OR 'Korean People's Republic' OR Rwanda OR Sierra Leone OR Somalia OR Sudan OR 'South Sudan' OR Syria OR 'Syrian Arab Republic' OR Togo OR Uganda OR Yemen OR 'Republic of Yemen' OR 'Yemen Arab Republic') OR ti(Afghanistan OR Burundi OR 'Burkina Faso' OR 'Central African Republic' OR 'Centrafrican Republic' OR Centrafique OR 'Central African Empire' OR Chad OR Congo OR 'Democratic Republic of Congo' OR Eritrea OR Ethiopia OR Guinea OR Gambia OR 'Guinea Bissau' OR Guinea-Bissau OR GuineaBissau OR Liberia OR Madagascar OR 'Malagasy Republic' OR Mali OR Malawi OR Mozambique OR Niger OR 'North Korea' OR 'Democratic People's Republic of Korea' OR 'Korean People's Republic' OR Rwanda OR Sierra Leone OR Somalia OR Sudan OR 'South Sudan' OR Syria OR 'Syrian Arab Republic' OR Togo OR Uganda OR Yemen OR 'Republic of Yemen' OR 'Yemen Arab Republic')) AND stype.exact("Conference Papers & Proceedings" OR "Scholarly Journals") AND at.exact("Article") AND pd(>20100101)  **2. World Bank eLibrary:**  #1 Abstract search ((dental OR oral OR teeth OR tooth) AND (care OR health OR service OR access* OR disparit* OR coverage OR insurance OR utilization OR spending OR expenditure OR financing OR package OR scheme OR plan OR strateg* OR utilization)) OR "dental facility" OR "dental facilities" OR "dental office" OR "dental offices"  #2 Title search ((dental OR oral OR teeth OR tooth) AND (care OR health OR service OR access* OR disparit* OR coverage OR insurance OR utilization OR spending OR expenditure OR financing OR package OR scheme OR plan OR strateg* OR utilization)) OR "dental facility" OR "dental facilities" OR "dental office" OR "dental offices"  #3 Keyword search ((dental OR oral OR teeth OR tooth) AND (care OR health OR service OR access* OR disparit* OR coverage OR insurance OR utilization OR spending OR expenditure OR financing OR package OR scheme OR plan OR strateg* OR utilization)) OR "dental facility" OR "dental facilities" OR "dental office" OR "dental offices"  **3. OECD iLibrary:**  #1 (All Fields ‘"dental facility" OR "dental facilities" OR "dental office*" OR ( ( dental OR oral OR teeth OR tooth ) N/2 ( care OR health OR service OR access* OR disparit* OR coverage OR insurance OR utilization OR spending OR expenditure OR financing OR package OR scheme OR plan OR strateg* OR utilisation ) )’) **AND** (All Fields ‘Afghanistan OR Burundi OR "Burkina Faso" OR "Central African Republic" OR "Centrafrican Republic" OR Centrafique OR "Central African Empire" OR Chad OR Congo OR "Democratic Republic of Congo" OR Eritrea OR Ethiopia OR Guinea OR Gambia OR "Guinea Bissau" OR Guinea-Bissau OR GuineaBissau OR Liberia OR Madagascar OR "Malagasy Republic" OR Mali OR Malawi OR Mozambique OR Niger OR "North Korea" OR "Democratic People Republic of Korea" OR "Korean People Republic" OR Rwanda OR "Sierra Leone" OR Somalia OR Sudan OR "South Sudan" OR Syria OR "Syrian Arab Republic" OR Togo OR Uganda OR Yemen OR "Republic of Yemen" OR "Yemen Arab Republic"’) **AND** ( ‘’) with type(s) subtype/journal OR subtype/article OR subtype/workingpaper published between 2010 and 2023  #2 (All Fields ‘Afghanistan OR Burundi OR "Burkina Faso" OR "Central African Republic" OR "Centrafrican Republic" OR Centrafique OR "Central African Empire" OR Chad OR Congo OR "Democratic Republic of Congo" OR Eritrea OR Ethiopia OR Guinea OR Gambia OR "Guinea Bissau" OR Guinea-Bissau OR GuineaBissau OR Liberia OR Madagascar OR "Malagasy Republic" OR Mali OR Malawi OR Mozambique OR Niger OR "North Korea" OR "Democratic People Republic of Korea" OR "Korean People Republic" OR Rwanda OR "Sierra Leone" OR Somalia OR Sudan OR "South Sudan" OR Syria OR "Syrian Arab Republic" OR Togo OR Uganda OR Yemen OR "Republic of Yemen" OR "Yemen Arab Republic"’) AND (All Fields ‘( ( dental OR oral OR teeth OR tooth ) AND ( care OR health OR service OR access* OR disparit* OR coverage OR insurance OR utilization OR spending OR expenditure OR financing OR package OR scheme OR plan OR strateg* OR utilization ) ) OR "dental facility" OR "dental facilities" OR "dental office" OR "dental offices"’) AND ( ‘’) with type(s) subtype/journal OR subtype/article OR subtype/workingpaper published between 2010 and 2023 |
| --- |

**Web Appendix 4. Information extraction from literature review**

We used a pilot-tested Excel template to extract the information below.

- Country name
- Article title
- In what language the article was written in
- Name of the author(s) or department(s) who developed the article
- Publication year
- Study aim
- Study design
- Data source upon which the article draws conclusions
- Period the data covered
- At what geographic level that the data were collected
- Integration of oral health care in national health policies
  - Time period the policy covers
  - Background, goal, objective, target, and strategy of the policy regarding oral health care
- Clinical and public oral health services included in government-defined essential health service list
  - Name of the health service package
  - Time period and population covered
  - Oral health care services included
  - Cost-sharing mechanism of oral health care services
- Oral health care utilization rate
  - Utilization rate
  - Disease or service the utilization rate refers to
  - Time period the utilization rate refers to
  - Geographic or institutional level the utilization rate refers to
  - Socioeconomic or demographic characteristics of the care receivers
- Oral health care expenditure
  - Time period the expenditure relates to
  - Bearer of the expenditure and the amount
  - In what unit and currency that the expenditure is recorded
  - On what items the expenditure is spent on
- Oral health workforce
  - Workforce data
  - What professional type the workforce data refer to
  - Time period the workforce data refer to
  - Geographic or institutional level the workforce data refer to

**Web Appendix 5. Oral health care keywords**

| abscess, alveolar, analgesic, anesthesia, antibiotic, ART^(1)^, assistant, atraumatic, bleed, brush, cancer, caries, cavity, cement, cleft, decay, dental, dentist, dento, denture, detect, diamine, discolor, drainage, drill, early, edentulism, emergency, enamel, erosion, examine, extract, filling, first aid, fluoride, follow, function, GIC^(2)^, gingivitis, glass, gum, hygienist, inclusion, intraoral, ionomer, IOPA^(3)^, jawbone, lip, maxilla, maxillofacial, mouth, mucosa, noma, nurse, odontogenic, odontology, oral, pain, palate, palliative, periapical, periodontal, periodontitis, pigment, prosthesis, prosthetic, reconstruct, relief, restoration, restorative, saline, saliva, school, screen, SDF^(4)^, sealant, silver, socket, stomatology, surface, swell, technician, teeth, therapist, tissue, tooth, toothpaste, trauma, trismus, ulcer, urgent, vanish, visit, x-ray |
| --- |

1. ART = Atraumatic Restorative Treatment; (2) GIC = Glass Ionomer Cement; (3) IOPA = Intra oral periapical radiography; (4) SDF = Silver Diamine Fluoride

**Reference**

1. World Health Organization. Oral Health. World Health Assembly Resolution WHA74/A74.R5. Geneva: World Health Organization, 2021. Available from https://apps.who.int/gb/ebwha/pdf_files/WHA74/A74_R5-en.pdf.
2. World Health Organization. Landmark global strategy on oral health adopted at World Health Assembly 75. Available from <https://www.who.int/news-room/feature-stories/detail/landmark-global-strategy-on-oral-health-adopted-at-world-health-assembly-75>
3. World Health Organization. Draft global oral health action plan (2023-2030). Available from <https://www.who.int/publications/m/item/draft-global-oral-health-action-plan-(2023-2030)>
4. Frencken JE, Holmgren C and Helderman VP (2002). WHO Basic Package of Oral Care (BPOC). Nijmegen, Netherlands: WHO Collaborating Centre for Oral Health Care Planning and Future Scenarios, University of Nijmegen. Available from <http://www.chdentalinstitute.org/images/bpoc.pdf>
5. World Health Organization. (2016). Promoting Oral Health in Africa: Prevention and control of oral diseases and noma as part of essential noncommunicable disease interventions.
6. Global oral health status report: towards universal health coverage for oral health by 2030. Geneva: World Health Organization; 2022. Licence: CC BY-NC-SA 3.0 IGO.
7. Peres, M. A., Macpherson, L. M., Weyant, R. J., Daly, B., Venturelli, R., Mathur, M. R., ... & Watt, R. G. (2019). Oral diseases: a global public health challenge. The Lancet, 394(10194), 249-260.
8. World Health Organization. UHC Compendium. Health interventions for Universal Health Coverage. Available from <https://www.who.int/universal-health-coverage/compendium#:~:text=The%20UHC%20Compendium%20is%20a,Universal%20Health%20Coverage%20(UHC)>. Access date: May 2023

**Web Appendix 6. Information sources utilized to gather evidence for analyzing each of the five research questions**

| **Research question** | **Seven bibliographic databases^1^** | **PAIS Index^2^** | **World Bank eLibrary^3^** | **OECD iLibrary^4^** | **WHO Country Planning Cycle Database^5^** | **WHO NCDs* Document Repository^6^** | **WHO MiNDbank^7^** | **Google search** | **WHO National Health Workforce Account^8^** | **WHO Global Health Expenditure Database^9^** | **WHO Oral Health Country Profile^10^** |
| --- | --- | --- | --- | --- | --- | --- | --- | --- | --- | --- | --- |
| Inclusion of oral health care in national health policies | X | X | X | X | X | X | X | X |  |  |  |
| Clinic and public oral health care services | X | X | X | X | X | X | X | X |  |  |  |
| Utilization rate of oral health care service | X | X | X | X | X | X | X | X |  |  |  |
| Oral health care expenditure | X | X | X | X | X | X | X | X |  | X | X |
| Number of oral health care professionals | X | X | X | X | X | X | X |  | X |  |  |

*. “NCDs” refers to Non-communicable Diseases

**Reference:**

1. Seven bibliographic databases include:

(1) PubMed. <https://pubmed.ncbi.nlm.nih.gov/>. Access date: May 2023

(2) Embase. <https://www.embase.com/landing?status=grey>. Access date: May 2023

(3) Web of Science. <https://clarivate.com/products/scientific-and-academic-research/research-discovery-and-workflow-solutions/webofscience-platform/>. Access date: May 2023

(4) EconLit. <https://www.ebsco.com/products/research-databases/econlit>. Access date: May 2023

(5) Global Health (EBSCO). <https://www.ebsco.com/products/research-databases/global-health>. Access date: May 2023

(6) WHO Global Index Medicus. <https://www.globalindexmedicus.net/>. Access date: May 2023

(7) Dentistry and Oral Sciences Source. <https://www.ebsco.com/products/research-databases/dentistry-oral-sciences-source>. Access date: May 2023

2. PAIS Index. <https://proquest.libguides.com/pais>. Access date: May 2023

3. World Bank eLibrary. <https://elibrary.worldbank.org/>. Access date: May 2023

4. OECD iLibrary. <https://www.oecd-ilibrary.org/>. Access date: May 2023

5. WHO Country Planning Cycle Database. <https://extranet.who.int/countryplanningcycles/>. Access date: May 2023

6. WHO NCDs Document Repository. <https://extranet.who.int/ncdccs/documents/Db>. Access date: May 2023

7. WHO MiNDbank. <https://extranet.who.int/mindbank/collection/country>. Access date: May 2023

8. WHO National Health Workforce Account. <https://apps.who.int/nhwaportal/>. Access date: May 2023

9. WHO Global Health Expenditure Database. <https://apps.who.int/nha/database/Select/Indicators/en>. Access date: May 2023

10. WHO Oral Health Country Profile. <https://www.who.int/team/noncommunicable-diseases/global-status-report-on-oral-health-2022>. Access date: January 2023

**Web Appendix 7. Summary of oral health information for each of the 55 included national health policy documents**

**1. Afghanistan**

| **No.** | **File title (sorted by the start year of each file’s targeted timeframe)** | **Inclusion of health service list (Y/N), inclusion of oral health service in the list (Y/N)** | **Related theme** | **Key findings** |
| --- | --- | --- | --- | --- |
| 1 | The Essential Package of Hospital Services for Afghanistan 2005/1384 | Y, Y | Service provision | - **[page 11, 22]:** Hospitals are classified into District Hospitals (DH), Provincial Hospitals (PH), and Regional Hospitals (RH). Dental and oral health diagnosis and treatment provided by types of hospitals: - **DH, PH, and RH:** abscess, periapical / acute necrotizing ulcerative gingivitis / cellulitis (oral) / gingivitis / pericoronitis / periodontitis / pulpitis. - **PH and RH:** alveolitis (dry socket) / jaw trauma (refer to Regional or Kabul tertiary hospital level if necessary). - **RH:** Temporomandibular joint disorders (refer to Kabul Hospital if necessary) - **Refer to Kabul Hospital:** salivary gland disease - **[page 14, 16, 17]:** Dental services are primarily provided as outpatient services at DH and PH and as inpatient services at RH. |
|  |  |  | Human resource | - **[page 32]:** Each DH is advised to have 1 dental technician for every 50 beds; each PH is advised to have 2 dental technicians and 1 dentist for every 150 beds; each RH is advised to have 4 dental technicians and 3 dentists for every 350 beds. |
| 2 | Basic Package of Health Service 2010 | Y, N | Policy inclusion | - **[page 9]:** Identifies dental health as a priority concern of the government. |
| 3 | National Health Workforce Plan 2012-2016 | N, N | Human resource | - **[page 23]:** Targets to have 4,019 dentists and dental technicians by the end of 2016, with the aim of 0.13 dentists and dental technicians per 1,000 population. - **[page 27]:** Targets to have 4,584 dentists and dental technicians by the end of 2020, with the aim of 0.15 dentists and dental technicians per 1,000 population. |
| 4 | National Reproductive, Maternal, Newborn, Child, and Adolescent Health (RMNCAH) Strategy 2017-2021 | N, N | School health | - **[page 50]:** Plans to train two teachers in each school to screen for dental problems as a means to improve school health services for adolescents. |
| 5 | Normative Costing of Basic Package of Health Services (BPHS) 2020 | N, N | Service use | - **[page 15]:** Among the 10 health programs covered in the Basic Package of Health Services, the dental health program has the lowest utilization. |
|  |  |  | Service cost | - **[page 20]:** For each individual service, dental health costs 1.8 USD, compared to 5.2 USD for mental health and 3.7 USD for maternal and newborn health. - **[page 29]:** For each health program, dental health accounted for 1.4% of the total cost across the 10 health programs in the Basic Package of Health Service, compared to 37.1% for child health and immunization, 17.6% for nutrition, and 16.8% for maternal and newborn health. |

**Reference**

1. Islamic Republic of Afghanistan. Ministry of Public Health. The Essential Package of Hospital Services for Afghanistan 2005/1384. Available from <https://platform.who.int/docs/default-source/mca-documents/policy-documents/guideline/afg-cc-46-01-guideline-2005-eng-essential-hospital-services.pdf>. Access date: May 2023.

2. World Health Organization. WHO MiNDbank. Available from <https://extranet.who.int/mindbank/item/5613>. Access date: May 2023.

3. Islamic Republic of Afghanistan 2011. Ministry of Public Health. Afghanistan National Health Workforce Plan 2012-2016. Available from <https://extranet.who.int/countryplanningcycles/sites/default/files/planning_cycle_repository/afghanistan/afghanistan_hrhplan_2012-2016_draft.pdf>. Access date: May 2023.

4. Islamic Republic of Afghanistan. Ministry of Public Health. National Reproductive, Maternal, Newborn, Child, and Adolescent Health (RMNCAH) Strategy 2017-2021. Available from <https://rmncah-moph.gov.af/wp-content/uploads/2017/11/National-RMNCAH-Strategy-2017-2021-English-Final.pdf>. Access date: May 2023.

5. Islamic Republic of Afghanistan. Ministry of Public Health. Normative Costing of Basic Package of Health Services (BPHS) 2020. Available from <https://moph.gov.af/sites/default/files/2020-11/BPHS%20Costing%20final%20Report%207-Nov-%202020_.pdf>. Access date: May 2023.

**2. Burkina Faso**

| **No.** | **File title (sorted by the start year of each file’s targeted timeframe)** | **Inclusion of health service list (Y/N), inclusion of oral health service in the list (Y/N)** | **Related theme** | **Key findings** |
| --- | --- | --- | --- | --- |
| 1 | Three-year Action Plan for the Fight Against Oral Diseases and Noma 2015-2017^(1)^ | N, N | Vision and objective | - **[page 25]:** Aims to improve the state of health and reduce the burden of noma on populations. - **[page 25]:** Identifies six objectives for addressing oral diseases and noma, including (1) capacity building and development of socio-health personnel; (2) capacity building and development at the community level; (3) sensitization and social mobilization; (4) development of training and education materials; (5) strengthening coordination and monitoring evaluation; (6) reinforcing coordination and leadership. |
|  |  |  | School health | - **[page 22]:** Plans to implement awareness-raising activities on oral diseases and noma in national schools of primary teachers and in national schools of public health. |
| 2 | Strategic Plan for the Health of the Elderly 2016-2020^(2)^ | N, N | Disease burden | - **[page 21-23]:** Recognizes that dental loss is among several other factors that contribute to the undernutrition and digestive disorders observed in the elderly. |
| 3 | Integrated Strategic Plan for the Fight Against Non-Communicable Diseases 2016-2020^(3)^ | N, N | Disease burden | - **[page 27]:** Identifies that, in Burkina Faso, the prevalence and severity of oral diseases are increasing due to lifestyle changes. The main oral diseases are dental caries, periodontal diseases, maxillofacial trauma, tumors of the oral cavity, dentofacial anomalies, oral manifestations of general diseases and HIV infection, and noma. - **[page 27]:** Estimates that only 31.5% of the population clean their teeth at least twice a day and a quarter (25.8%) use fluoridated toothpaste. |
|  |  |  | Policy inclusion | - **[page 33]:** Several NCDs, including oral diseases and noma, have been the subject of specific programs led by the Ministry of Health. |
|  |  |  | Human resource | - **[page 78]:** Targets to train 150 health professionals each year during the timeframe of the plan, including professionals in oral health. |
|  |  |  | School health | - **[page 85]:** By the end of 2020, increase the proportion of primary and secondary schools where students have been sensitized about oral health. |

Note (File titles in original language):

(1) Plan d’Action Triennal de Lutte contre les Maladies Bucco-Dentaires et le Noma 2015-2017

(2) Plan stratégique de santé des personnes âgées 2016-2020

(3) Plan Stratégique Intégré de Lutte contre les Maladies Non Transmissibles 2016-2020

**Reference**

1. Burkina Faso. Ministere De La Sante. Programme National Des Maladies Non Transmissibles Unite De Sante Orale. Plan D’action Triennal De Lutte Contre Les Maladies Bucco-Dentaires Et Le Noma 2015 – 2017 [Burkina Faso. Ministry of Health. National Non-Communicable Diseases Program Oral Health Unit. Three-Year Action Plan for the Fight Against Oral Diseases and Noma 2015-2017]. Available from <https://extranet.who.int/ncdccs/Data/BFA_B8_Plan%20%20d'Action%20National%20%20Triennal%20Noma%20BURKINA%20FASO%20VF.pdf>. Access date: May 2023.

2. Burkina Faso. Ministere De La Sante. Plan stratégique de santé des personnes âgées 2016 – 2020 [Burkina Faso. Ministry of Health. Strategic Plan for the Health of the Elderly 2016-2020]. Available from <https://extranet.who.int/countryplanningcycles/sites/default/files/planning_cycle_repository/burkina_faso/plan_strategique_de_sante_des_personnes_agees_2016_-_2020.pdf>. Access date: May 2023.

3. Burkina Faso. Ministere De La Sante. Plan Strategique Integre De Lutte Contre Les Maladies Non Transmissibles 2016-2020 [Burkina Faso. Ministry of Health. Integrated Strategic Plan for the Fight Against Non-Communicable Diseases 2016-2020]. Available from <https://extranet.who.int/ncdccs/Data/BFA_B3_Plan%20SIMNT_FINAL_27-09-2016_F.pdf>. Access date: May 2023.

**3. Burundi**

| **No.** | **File title (sorted by the start year of each file’s targeted timeframe)** | **Inclusion of health service list (Y/N), inclusion of oral health service in the list (Y/N)** | **Related theme** | **Key findings** |
| --- | --- | --- | --- | --- |
| 1 | Profile of Human Resources in Health of Burundi 2011^(1)^ | N, N | Human resource | - **[page 18]:** In 2010, there were only two dentists in the country. |
| 2 | National Health Policy 2016-2025^(2)^ | N, N | Policy inclusion | - **[page 20]:** Acknowledges that NCDs, including oral health, constitute the concerns of the population and deserve special attention in health planning. - **[page 48]:** Stresses that the government commits to NCDs including oral health. |
|  |  |  | School health | - **[page 52]:** Within the framework of the promotion of school health, plans to prioritize oral health for children and protect children from harmful traditional practices (e.g., removal of the uvula and dental buds). |
| 3 | Multisectoral Action Plan for the Prevention and Control of Non-Communicable Diseases 2019-2023^(3)^ | Y, N | Disease burden | - **[page 10-11]:** Acknowledges that NCDs, including oral health, have gradually become the main cause of mortality and morbidity in the country. |

Note (File titles in original language):

(1) Profil de Ressources Humaines en Sante du Burundi 2011.

(2) Politique Nationale de Sante 2016-2025.

(3) Plan d’Action Multisectoriel de Prévention et de Contrôle des Maladies Non Transmissibles 2019-2023.

**Reference**

1. Republique Du Burundi. Ministere De La Sante Publique Et De La Lutte Contre Le Sida. Profil De Ressources Humaines En Sante Du Burundi 2011. [Republic of Burundi. Ministry of Public Health and the Fight Against AIDS. Profile of Human Resources in Health of Burundi 2011]. Available from <https://extranet.who.int/countryplanningcycles/sites/default/files/planning_cycle_repository/burundi/profil_rh_vf_22_janvier_20121.pdf>. Access date: May 2023.

2. Republique Du Burundi. Politique Nationale de Sante 2016-2025. [Republic of Burundi. National Health Policy 2016-2025] Available from <https://extranet.who.int/countryplanningcycles/sites/default/files/planning_cycle_repository/burundi/pns_2016_2025_burundi.pdf>. Access date: May 2023.

3. Republique Du Burundi. Ministere De La Sante Publique Et De La Lutte Contre Le Sida. Plan D’Action Multisectoriel De Prevention Et De Controle Des Maladies Non Transmissibles 2019-2023. [Republic of Burundi. Ministry of Public Health and the Fight Against AIDS. Multisectoral Action Plan for the Prevention and Control of Non-Communicable Diseases 2019-2023]. Available from <https://extranet.who.int/ncdccs/Data/BDI_B3_PAM%20FINAL%2023%2007%202019.pdf>. Access date: May 2023.

**4. Central African Republic**

| **No.** | **File title (sorted by the start year of each file’s targeted timeframe)** | **Inclusion of health service list (Y/N), inclusion of oral health service in the list (Y/N)** | **Related theme** | **Key findings** |
| --- | --- | --- | --- | --- |
| 1 | National Policy Document for the Prevention and Fight Against Non-Communicable Diseases 2014^(1)^ | N, N | Policy inclusion | - **[page 7]:** Plans to develop monitoring indicators for oral disease as part of the strategies to supervise and evaluate population health status. - **[page 7]:** Plans to establish a Technical Steering Working Group to implement programs to combat NCDs, including oral diseases. |
| 2 | Health Sector Transition Plan 2015-2017^(2)^ | N, N | Human resource | - **[page 28]:** In 2014, the country had a combined total of 267 doctors, pharmacists, and dentists, while the required workforce is 485 for these three categories. |

Note (File titles in original language):

(1) Document de Politique Nationale de Prévention et de Lutte contre les Maladies Non Transmissibles 2014

(2) Plan de Transition du Secteur Santé en République Centrafricaine 2015-2017

**Reference**

1. République Centr Africaine. Ministere De La Sante Publique. Document De Politique Nationale De Prevention Et De Lutte Contre Les Maladies Non Transmissibles 2014. [Central African Republic. Minister of Public Health. National Policy Document for the Prevention and Fight Against Non-Communicable Diseases 2014]. Available from <https://extranet.who.int/ncdccs/Data/CAF_B3_POLITIQUE%20MNT%20REPUBLIQUE%20CENTRAFRICAINE.pdf>. Access date: May 2023.

2. République Centr Africaine. Ministere De La Sante Publique. Plan de Transition du Secteur Santé en République Centrafricaine 2015-2017. [Central African Republic. Minister of Public Health. Health Sector Transition Plan 2015-2017]. Available from <https://extranet.who.int/countryplanningcycles/sites/default/files/planning_cycle_repository/central_african_republic/rca_-ptss_revise_final_26092016.pdf>. Access date: May 2023.

**5. Chad**

| **No.** | **File title (sorted by the start year of each file’s targeted timeframe)** | **Inclusion of health service list (Y/N), inclusion of oral health service in the list (Y/N)** | **Related**  **theme** | **Key findings** |
| --- | --- | --- | --- | --- |
| 1 | Multisectoral Plan for the Fight and Control of Non-Communicable Diseases 2017-2021^(1)^ | N, N | Disease  burden | - **[page 34]:** Identifies an increasing trend of oral diseases in Chad. |
|  |  |  | Policy inclusion | - **[page 41]:** Identifies that there are very few health programs/facilities that have protocols for the prevention and treatment of NCDs. The National Reference General Hospital is the only hospital facility that provides the vast majority of specialized treatment of NCDs, including oral diseases. |
|  |  |  | Policy inclusion | - **[page 44]:** Acknowledges the absence of an action plan for oral health. - **[page 60]:** Includes four activities to fight against oral disease and noma within the framework of primary health care, including (1) reproduce and disseminate communication tools on oral diseases and noma; (2) organize training workshops in the use of communication and monitoring tools on oral diseases and noma; (3) organize quarterly awareness sessions; (4) organize an annual screening campaign on oral diseases and noma. |

Note (File titles in original language):

(1) Plan Multisectoriel de Lutte et de Contrôle des Maladies Non Transmissibles 2017-2021

**Reference**

1. République du Tchad. Ministère de la Santé Publique. Plan Multisectoriel de Lutte et de Contrôle des Maladies Non Transmissibles 2017-2021. [Republic of Chad. Minister of Public Health. Multisectoral Plan for the Fight and Control of Non-Communicable Diseases 2017-2021] Available from <https://extranet.who.int/ncdccs/Data/TCD_B3_PLAN%20MULTISEC%20MNT.pdf>. Access date: May 2023.

**6. Congo DR**

| **No.** | **File title (sorted by the start year of each file’s targeted timeframe)** | **Inclusion of health service list (Y/N), inclusion of oral health service in the list (Y/N)** | **Related theme** | **Key findings** |
| --- | --- | --- | --- | --- |
| 1 | Oral Health Strategic Plan 2021-2022^(1)^ | N, N | Issues in oral care provision | - **[page 22]:** Service delivery issues, including low coverage of oral care, low quality of oral care and services offered, low utilization of oral care. - **[page 23]:** Human resources for the provision of oral care are insufficient and unevenly distributed; lack of infrastructure and equipment for oral care; the national essential drug supply system does not take into account the needs for drugs and specific inputs for oral care. - **[page 26]:** Households are the main source of health financing, the state budget allocated to oral health services and care is very low. Oral health care does not yet benefit from external funding and other partners. - **[page 26]:** Oral health care does not have an information management tool specific or aligned to the national system. |
|  |  |  | Vision, objective,  strategy | - **[page 29]:** Aims to advance the National Oral Health Program towards universal access to quality oral and dental services by 2022. - **[page 30]:** Aims to reduce the prevalence of dental caries and other oral diseases among children, adolescents, and pregnant women by 30% by 2022; ensure that 20% of children, adolescents, and pregnant women in need have access to oral health care services. - **[page 30]:** Aims to have 10% of the primary health care facilities to provide essential and safe oral health care by 2022. - **[page 37]:** Identifies seven strategies, including (1) integrate oral care interventions into the Minimum Package of Activities and the Complementary Package of Activities; (2) improve the production and equitable distribution of oral health care human resources; (3) improve the availability of oral care infrastructure and medical equipment; (4) align oral care drug to the national drug supply system; (5) mobilize financial resources and develop risk-sharing mechanisms for oral care; (6) integrate oral health information into the national health information system; (7) strengthen governance and management of oral health. |
|  |  |  | School health | - **[page 32]:** Targets to organize active screening for oral disease in schools in collaboration with the national school and university health program. |

Note (File titles in original language):

(1) Plan Stratégique de la Santé Bucco-Dentaire 2021-2022

**Reference**

1. République Démocratique du Congo. Ministere De La Sante. Plan Stratégique de la Santé Bucco-Dentaire 2021-2022. [Democratic Republic of Congo. Health Ministry. Oral Health Strategic Plan 2021-2022]. Available from <https://extranet.who.int/ncdccs/Data/COD_B8_s21_DRC_Plan%20Strategique%20SBD%202021%202022.docx>. Access date: May 2023.

**7. Eritrea**

| **No.** | **File title (sorted by the start year of each file’s targeted timeframe)** | **Inclusion of health service list (Y/N), inclusion of oral health service in the list (Y/N)** | **Related theme** | **Key findings** |
| --- | --- | --- | --- | --- |
| 1 | The Second Health Sector Strategic Development Plan II 2017-2021 | N, N | Policy inclusion | - **[page 58]:** Identifies oral and dental condition as a focus to prevent NCDs. |
|  |  |  | Objective | - **[page 59]:** Identifies priority interventions for oral and dental health, including maintaining 550 trained health workers on dental health by 2021, developing oral and dental care policy and strategy by 2019, having 35,000 people sensitized on oral and dental health by 2021, having 50% of health facilities providing oral and dental services by 2021, and conducting regular monitoring and supervision on oral health. |
| 2 | Strategic Plan for the Implementation of Reproductive, Maternal, Newborn, Child and Adolescent Health and Healthy Ageing Programmes in Eritrea 2022-2026 | Y, Y | School and elderly health | - **[page 69]:** Includes the screening and treatment of dental health conditions for school children and the elderly, as an evidence-based high-impact intervention. |

**Reference**

1. Eritrea. Ministry of Health. The Second Health Sector Strategic Development Plan II 2017-2021. Available from <https://extranet.who.int/countryplanningcycles/sites/default/files/planning_cycle_repository/eritrea/eritrea_hssdp_ii_21022017.pdf>. Access date: May 2023.

2. Eritrea. Ministry of Health. Strategic Plan for the Implementation of Reproductive, Maternal, Newborn, Child and Adolescent Health and Healthy Ageing Programmes in Eritrea 2022-2026. Available from <https://www.afro.who.int/sites/default/files/2022-09/2022_RMNCAH_Eritrea.pdf>. Access date: May 2023.

**8. Ethiopia**

| **No.** | **File title (sorted by the start year of each file’s targeted timeframe)** | **Inclusion of health service list (Y/N), inclusion of oral health service in the list (Y/N)** | **Related theme** | **Key findings** |
| --- | --- | --- | --- | --- |
| 1 | Health Sector Transformation Plan 2015-2020 | N, N | Policy inclusion | - **[page 87]:** Identifies the improvement of oral health services as a key component to enhance equitable access to quality health services. |
| 2 | Essential Health Services Package 2019 | Y, Y | Service provision | - Identifies the following oral health services to be included in the Essential Health Services Package (with priority and cost-sharing mechanism): - **[page 42]:** New-born care: Detection of cleft lip, palate (high priority), cost free. - **[page 55]:** Lip and oral cancer: diagnosis of lip and oral cancer (high priority), treatment with chemo/hormonal therapy for lip and oral cancer (medium priority), lip and oral cancer treatment radiotherapy and chemotherapy (low priority), all with shared cost. - **[page 63]:** Non-trauma surgical conditions: repair of cleft lip and palate (high priority), with shared cost. - **[page 66]:** Oral and dental procedure: extraction of primary and permanent tooth (high priority), periodontal and dental abscess incision and drainage (high priority), dental caries treatment and scaling (high priority), management facial bone fractures and/or dislocation and injury to dentition (inter-dental wiring, arch bar, IMF and open reduction) (high priority), management of oro-facial infection (high priority), management of common benign tumors and cysts of oral and maxillofacial regions (low priority), management of common malignant tumors and cysts of oral and maxillofacial regions (medium priority), dental trauma care (high priority), all with shared cost. - **[page 68]:** Advanced emergency care services: Acute management of dental emergencies (high priority), with shared cost. - **[page 70]:** Personal hygiene: create awareness on proper oral hygiene practices (high priority), cost free. |
| 3 | National Strategic Plan for the Prevention and Control of Major Non-Communicable Diseases 2021-2025 | N, N | Disease burden | - **[page 13]:** Identifies the public health concern of oral diseases in Ethiopia. - **[page 24]:** Recognizes that the risk factors for oral diseases in Ethiopia include unhealthy diet, tobacco use, harmful use of alcohol, physical inactivity, khat, raised blood glucose, and infections. |

**Reference**

1. Ethiopia. Ministry of Health. Health Sector Transformation Plan 2015-2020. Available from <https://extranet.who.int/countryplanningcycles/sites/default/files/planning_cycle_repository/ethiopia/hstp_ethiopia.pdf>. Access date: May 2023.

2. Ethiopia. Ministry of Health. Essential Health Services Package 2019. Available from <https://www.uib.no/sites/w3.uib.no/files/attachments/essential_health_service_package_ethiopia_2019_0.pdf>. Access date: May 2023.

3. Ethiopia. Ministry of Health. National Strategic Plan for the Prevention and Control of Major Non-Communicable Diseases 2021-2025. Available from <https://extranet.who.int/ncdccs/Data/ETH_B3_s21_National_Strategic_Plan_for_Prevention_and_Control_of_NCDs2021.pdf>. Access date: May 2023.

**9. Gambia**

| **No.** | **File title (sorted by the start year of each file’s targeted timeframe)** | **Inclusion of health service list (Y/N), inclusion of oral health service in the list (Y/N)** | **Related theme** | **Key findings** |
| --- | --- | --- | --- | --- |
| 1 | National Ageing Policy 2010 | N, N | Service provision | - **[page 49]:** Recognizes that affordable dental services should be provided to help prevent and treat disorders that can impede eating by older persons. |
| 2 | National Health Strategic Plan 2014-2020 | Y, Y | Service provision | - **[page 26]:** States that the country’s Minimum Health Care Package includes specialized dental care services at regional hospitals. |
| 3 | National Multi-Sectoral Strategy and Costed Action Plan for Non-Communicable Disease Prevention and Control in The Gambia 2022-2027 | N, N | Policy inclusion | - **[page 16]:** Recognized the limited interventions for oral health as a weakness of the NCDs prevention and control in the country. |

**Reference**

1. Gambia. Ministry of Employment and Social Welfare. National Ageing Policy Ageing with Security and Dignity 2010. Available from <https://extranet.who.int/countryplanningcycles/sites/default/files/planning_cycle_repository/gambia/national_ageing_policy_2010.pdf>. Access date: May 2023.

2. Gambia. Ministry of Health and Social Welfare. National Health Strategic Plan 2014-2020. Available from <https://extranet.who.int/countryplanningcycles/sites/default/files/planning_cycle_repository/gambia/gnhsp_-_final_draft_13oct2014.pdf>. Access date: May 2023.

3. Gambia. Ministry of Health. National Multi-Sectoral Strategy and Costed Action Plan for Non-Communicable Disease Prevention and Control in The Gambia 2022-2027. Available from <https://www.afro.who.int/sites/default/files/2022-07/National%20Multi-sectoral%20Strategy%20and%20Costed%20Action%20Plan%20for%20NCD%20prevention%20and%20control%20-Gambia%202022-27.pdf>. May 2023.

**10. Guinea**

| **No.** | **File title (sorted by the start year of each file’s targeted timeframe)** | **Inclusion of health service list (Y/N), inclusion of oral health service in the list (Y/N)** | **Related**  **theme** | **Key findings** |
| --- | --- | --- | --- | --- |
| 1 | National Health Development Plan 2015-2024^(1)^ | N, N | Policy inclusion | - **[page 54]:** Identifies the treatment of tooth decay as a priority of the plan; by 2024, the country aims to cover 100% of the treatment of tooth decay. - **[page 120]:** By 2024, the country aims to achieve 80% of coverage rate for dental cleaning and preventive care, and 50% for oral cancer treatment. |

Note (File titles in original language):

(1) Plan national de développement sanitaire 2015-2024

**Reference**

1. Guinee. Ministere De La Sante. Plan national de développement sanitaire 2015-2024. [Guinea. Ministry of Health. National Health Development Plan 2015-2024] Available from <https://extranet.who.int/countryplanningcycles/sites/default/files/public_file_rep/GIN_Guinea_Plan-national-de-developmment-sanitaire_2015-2024.pdf>. Access date: May 2023.

**11. Guinea-Bissau**

| **No.** | **File title (sorted by the start year of each file’s targeted timeframe)** | **Inclusion of health service list (Y/N), inclusion of oral health service in the list (Y/N)** | **Related theme** | **Key findings** |
| --- | --- | --- | --- | --- |
| 1 | National Program to Fight against Noma Action Plan 2016^(1)^ | N, N | Disease burden | - **[page 5]:** Identifies an increasing trend of noma cases in the country from 2007 to 2013. |
|  |  |  | Vision and objective | - **[page 6]:** Plans to improve the population health by intensifying the fight against oral and dental diseases and noma. - **[page 6]:** Identifies five objectives to achieve the plan, including (1) reinforce and develop the capacities of socio-sanitary personnel; (2) strengthen and develop capacities at community level; (3) raise awareness and mobilize society; (4) monitor and evaluate the implementation of strategies to fight oral and dental diseases and noma; (5) reinforce coordination and leadership. |

Note (File titles in original language):

(1) Programa Nacional de Luta contra Noma Plano de Ação das Atividades 2016

**Reference**

1. Guiné Bissau. Ministério Da Saúdepública. Programa Nacional de Luta contra Noma Plano de Ação das Atividades 2016. [Guinea-Bissau. Ministry of Public Health. National Program to Fight against Noma Action Plan 2016] Available from <https://extranet.who.int/ncdccs/Data/GNB_B8_Plan%20op%c3%a9rationnel%20NOMA2016_GNB.pdf>. Access date: May 2023.

**12. Liberia**

| **No.** | **File title (sorted by the start year of each file’s targeted timeframe)** | **Inclusion of health service list (Y/N), inclusion of oral health service in the list (Y/N)** | **Related theme** | **Key findings** |
| --- | --- | --- | --- | --- |
| 1 | Essential Package of Health Services for Universal Health Coverage 2022 | Y, Y | Service provision | - **[page 32, 34, 36, 38, 40]:** Includes dental extraction at health centers as a high priority intervention, drainage of dental abscess at health centers as a medium priority intervention, repair of cleft lip and cleft palate at tertiary hospitals as a medium priority intervention, education of school children on oral health through the community platform as a low priority intervention, treatment of caries at health centers as a low priority intervention. |
|  |  |  | Service cost | - **[page 50]:** Dental extraction is included in the package, and it costs US$ 0.19 per capita to be delivered at health centers. |

**Reference**

1. Liberia. Ministry of Health. Essential Package of Health Services for Universal Health Coverage 2022. Available from <https://www.dcp-3.org/sites/default/files/resources/MOH%20EPHS%20for%20UHC_Final%20Version%2020221121.pdf>. Access date: May 2023.

**13. Madagascar**

| **No.** | **File title (sorted by the start year of each file’s targeted timeframe)** | **Inclusion of health service list (Y/N), inclusion of oral health service in the list (Y/N)** | **Related theme** | **Key findings** |
| --- | --- | --- | --- | --- |
| 1 | National Oral Health Policy 2010-2020^(1)^ | N, N | Vision, objectives, strategies | - **[page 16]:** Aims to establish an integrated, efficient oral health system, accessible to all and contributing effectively to the well-being of the population. - **[page 16]:** Identifies seven objectives, including: (1) reduce the prevalence and incidence of oral diseases; (2) integrate oral health into other health programs; (3) increase the level of knowledge of the population in terms of oral health for a favorable behavior change; (4) improve the quality of oral care services; (5) ensure continuous capacity building of providers in oral health; (6) increase oral health coverage; (7) increase the availability, accessibility and use of dental practices. - **[page 17]:** Identifies eight strategic directions, including: (1) integration of oral diseases with all other diseases having common risk factors; (2) reinforcement of Behavior Change Communication (BCC) of populations in favor of oral health; (3) strengthening the skills of staff at all levels of health structures, including the professional career plan; (4) extension of geographical coverage and improvement of the quality of services offered; (5) strengthening partnership at all levels and in all areas; (6) fairness of service offer; (7) monitoring and evaluation of programs (including salt fluoridation, orofacial cancers and noma); (8) development of operational research. |
| 2 | Health Sector Development Plan 2015-2019^(2)^ | Y, Y | Service provision  and objective | - **[page 21]:** Thematic strategies, such as those relating to oral health, have been developed, but have not been systematically translated into operational plans. - **[page 116]:** Targets to increase the utilization rate of dental center from 10% in 2013 to 20% in 2019. - **[page 157, 166, 175]:** Plans coverage rate of oral health service in 2019 under three scenarios: - Scenario 1 (meet all the health needs of the country and under the overall budget constraints): oral cancer treatment (35%), prevention of dental caries (5%), dental caries care (65%), dental extraction (50%). - Scenario 2 (prioritize maternal and child health): oral cancer treatment (23%), prevention of dental caries (3%), dental caries care (43%), dental extraction (33%). - Scenario 3 (reduce all coverage and interventions by one third): oral cancer treatment (23%), prevention of dental caries (3.3%), dental caries care (43%), dental extraction (33%). |
|  |  |  | School health | - **[page 49]:** Confirms that the promotion of oral health carried out by health facilities and teachers has been implemented to improve child health in public primary schools. |
| 3 | National Policy for Integrated Control of Non-Communicable Diseases and Prevention of Disability 2017^(3)^ | N, N | Risk factor | - **[page 15]:** Recognizes that the high prevalence of NCDs risk factors, such as smoking, excessive alcohol consumption, insufficient consumption of fruits and vegetables, exposed the population to periodontal disease, premature loosening of teeth, oral cancer, and cavities. |
|  |  |  | Dental care facilities | - **[page 15]:** Efforts to increase the number of dental practices have been undertaken over the past ten years, resulting in the creation of dental centers in all the chief towns; however, this infrastructure development suffers from the lack of outgoing or recruited dentists. The utilization rate of dentistry centers is only at 11%. |
| 4 | National Strategic Plan for Health of the Elderly 2018^(4)^ | N, N | Disease burden | - **[page 25]:** Stresses a high prevalence of caries and periodontal diseases (80%) among all age population, and a high prevalence of total loss of teeth (10%) among the elderly. |
|  |  |  | Service cost | - **[page 30]:** Recognizes that the dental prostheses are not always accessible to the elderly because of the high cost, making the devices practically beyond the reach of most budgets. |
|  |  |  | School and elderly health | - **[page 42, 53]:** Reinforces the promotion of health and well-being throughout life as a priority intervention to improve active aging; aims to achieve 80% of targeted district primary schools to have at least one awareness session on oral hygiene in 2023. - **[page 57]:** Targets to have 50% of dental prostheses delivered at affordable cost to the elderly in 2023. |

Note (File titles in original language):

(1) Politique Nationale de Sante Bucco-Dentaire 2010-2020

(2) Plan de Développement du Secteur Santé 2015-2019

(3) Politique Nationale de Lutte Intégrée contre les Maladies Non Transmissibles et de Prévention du Handicap 2017

(4) Plan Stratégique National de Santé des Personnes Âgées 2018

**Reference**

1. Madagascar. Ministere De La Sante Publique. Politique Nationale de Sante Bucco-Dentaire 2010-2020. [Madagascar. Minister of Public Health. National Oral Health Policy 2010-2020]. Available from <https://extranet.who.int/ncdccs/Data/MDG_B8_PNSBD%20final_photos%2005_%20juin_10(1).doc>. Access date: May 2023.

2. Madagascar. Ministre de la Sante Publique. Plan de Développement du Secteur Santé 2015-2019. [Madagascar. Minister of Public Health. Health Sector Development Plan 2015-2019] Available from <https://extranet.who.int/countryplanningcycles/sites/default/files/planning_cycle_repository/madagascar/pdss_2015.pdf>. Access date: May 2023.

3. Madagascar. Ministre de la Santé Publique. Politique Nationale de Lutte Intégrée contre les Maladies Non Transmissibles et de Prévention du Handicap 2017. [Madagascar. Minister of Public Health. National Policy for Integrated Control of Non-Communicable Diseases and Prevention of Disability 2017] Available from <https://extranet.who.int/ncdccs/Data/MDG_B3_2%20DOC%20PolitiqueMNT_PH_020718_.pdf>. Access date: May 2023.

4. Madagascar. Ministre de la Santé Publique. Plan Stratégique National de Santé des Personnes Âgées 2018. [Madagascar. Minister of Public Health. National Strategic Plan for Health of the Elderly 2018] Available from <http://www.sante.gov.mg/ministere-sante-publique/wp-content/uploads/2021/07/Plan-Strat%C3%A9gique-National-de-la-Sant%C3%A9-des-Personnes-Ag%C3%A9es-Madagascar.pdf>. Access date: May 2023.

**14. Malawi**

| **No.** | **File title (sorted by the start year of each file’s targeted timeframe)** | **Inclusion of health service list (Y/N), inclusion of oral health service in the list (Y/N)** | **Related**  **theme** | **Key findings** |
| --- | --- | --- | --- | --- |
| 1 | Health Sector Strategic Plan II 2017-2022 | Y, Y | Disease  burden | - **[page 25]:** Stresses that, in 2014, 50% of school children (6-9 years) had tooth decay and the prevalence of tooth decay among 12-17 years old was about 78%. |
|  |  |  | Policy  inclusion | - **[page 25]:** A national Oral Health Week that focuses on oral health preventive and control measures such as dental health education, screening and treatment and tooth fluoridation has been instituted as an annual event; over 3 million school pupils were educated, screened, and fluoridated over the last five years. |
|  |  |  | Service  provision | - **[page 47]:** Confirmed that the Basic Health Care Package provides the management of severe and mild tooth pain, tooth extraction, and tooth filling at both the primary and secondary health care level. |

**Reference**

1. Malawi. Ministry of Health. Health Sector Strategic Plan II 2017-2022. Available from <https://extranet.who.int/countryplanningcycles/sites/default/files/planning_cycle_repository/malawi/health_sector_strategic_plan_ii_030417_smt_dps.pdf>. Access date: May 2023.

**15. Mali**

| **No.** | **File title (sorted by the start year of each file’s targeted timeframe)** | **Inclusion of health service list (Y/N), inclusion of oral health service in the list (Y/N)** | **Related theme** | **Key findings** |
| --- | --- | --- | --- | --- |
| 1 | Ten-year Health and Social Development Plan 2014-2023^(1)^ | N, N | School  health | - **[page 40, 58]:** Targets to reduce the prevalence rate of dental caries for school-aged children (<10 years) from 82.4% in 2011 to <50% in 2023. |
| 2 | Oral Health Strategic Plan 2018-2022^(2)^ | N, N | Vision, objective, strategy | - **[page 38]:** Aims to improve the oral health of the population and the survival of children. - **[page 38]:** Includes six objectives, including (1) reduce morbidity and mortality related to oral diseases in Mali by the end of 2022; (2) ensure the promotion and prevention of oral diseases; (3) strengthen health systems for the management of oral diseases; (4) ensure the development of basic operational research in oral health; (5) strengthen the partnership in the fight against oral diseases; (6) establish a monitoring-evaluation mechanism. - **[page 38]:** Identifies six strategic axes, including (1) strengthening the capacities of health personnel in health structures for the early detection and effective management of oral diseases; (2) extension of geographical and health coverage in terms of oral health; (3) communication for the change of behavior of populations in favor of oral health; (4) reinforcement of the partnership in the prevention and the fight against oral diseases; (5) integration of the epidemiological surveillance of oral diseases into the existing epidemiological surveillance system; (6) development of research in the field of oral health. |
|  |  |  | School  health | - **[page 43]:** Includes the implementation of oral disease research at the school level as a strategic axis. |

Note (File titles in original language):

(1) Plan Décennal de Développement Sanitaire et Social (PDDSS) 2014-2023

(2) Plan Stratégique de Santé Bucco-Dentaire 2018-2022

**Reference**

1. Mali. Ministère de la Santé et de l’Hygiène Publique. Ministère du Travail et des Affaires Sociales et Humanitaires. Ministère de la Promotion de la Femme, de la Famille et de l’Enfant. Plan Décennal de Développement Sanitaire et Social (PDDSS) 2014-2023. [Mali. Ministry of Health and Public Hygiene. Ministry of Labor and Social and Humanitarian Affairs. Ministry for the Promotion of Women, Families and Children. Ten-year Health and Social Development Plan 2014-2023]. Available from <https://www.childrenandaids.org/sites/default/files/2018-05/Mali_Nat%20Health%20Plan_2014-2023%20fr.pdf>. Access date: May 2023.

2. Mali. Plan Stratégique de Santé Bucco-Dentaire 2018-2022. [Mali. Oral Health Strategic Plan 2018-2022]. Available from <https://extranet.who.int/ncdccs/Data/MLI_B8_Plan%20d'Action%20Sant%c3%a9%20Bucco-Dentaire%202018-2022.pdf>. Access date: May 2023.

**16. Mozambique**

| **No.** | **File title (sorted by the start year of each file’s targeted timeframe)** | **Inclusion of health service list (Y/N), inclusion of oral health service in the list (Y/N)** | **Related**  **theme** | **Key findings** |
| --- | --- | --- | --- | --- |
| 1 | Health Sector Strategic Plan 2014-2019 | Y, Y | Policy  inclusion | - **[page 78]:** Includes an oral health program to improve the oral health status of the Mozambican population. - strategies and interventions for access, including the design of oral health education strategies, prevention of oral disease at the community level, mass expansion of the use of fluoride at the community level through the use of toothpastes containing fluoride, addition of fluoride to water, the use of fluoride-based mouthwash in children aged 6-12, improvement of oral health knowledge among school-age children, promotion of healthy behavior, and increase in the number of qualified oral care professionals. - strategies and interventions for quality and humanization, including defining a basic dental care package for each level of medical care, increasing the quality of infrastructure, and enhancing the capabilities of specialized health workers in the maintenance of dental equipment. - strategies and interventions for effectiveness and efficiency, including improving the management capabilities of the oral health program, conducting studies to assess the prevalence of major oral diseases, and designing an epidemiological framework for oral disease. - strategies and interventions for better partnership, including multisectoral collaboration (e.g., private public partnerships) around oral health determinants with the aim of promoting oral health and distributing preventive materials. |
|  |  |  | Service  cost | - **[page 138]:** The cost of implementing the oral health program is estimated to be 31 million US dollars for 2014-2019. |
| 2 | National Oral Health Strategy 2019-2024^(1)^ | N, N | Utilization | - **[page 16]:** In 2018, only 17% of 1,600 existing health units provided oral health services, covering almost all district headquarters. - **[page 16]:** In 2018, only 2% of the population sought oral health services. Among the 836,121 individuals who sought oral health services, only 13% received dental treatment, while 52% required extractions. - **[page 16]:** In 2018, 2,933 dental prostheses were provided. However, out of the total 433,231 individuals who had teeth extracted, only 1% had access to dental prosthetics. |
|  |  |  | Human  resources | - **[page 17]:** Currently, the country has a total of 531 oral health professionals, with a dentist-to-population ratio of 1/52,280. |
|  |  |  | Financial  resources | - **[page 17]:** The Gross Domestic Product per capita in 2016 was 599USD, of which 10.34% is allocated to the health sector, of which 1.8% is allocated to the oral health program. |
|  |  |  | Vision and objective | - **[page 20]:** Stresses that the entire Mozambican population, especially children and adolescents, should enjoy good oral health free from oral diseases through Universal Health Coverage. - **[page 23]:** Identified four objectives, including (1) strengthen national advocacy, leadership, and partnerships to address oral diseases as part of NCDs through a multisectoral approach; (2) reduce common risk factors by promoting oral health and ensuring access to adequate fluorides; (3) strengthen the capacity of the health system for the prevention and integrated control of oral diseases.; (4) improve integrated oral disease surveillance, monitoring, evaluation programs, and oral health research. |
|  |  |  | School health | - **[page 26]:** Includes the development and implementation of daily interventions, such as tooth brushing, at schools as an objective. |
| 3 | Strategic Plan Multisectoral of Prevention and Control of Non-Communicable Diseases 2020-2029^(2)^ | N, N | Disease burden | - **[page 8]:** While primarily focuses on cancer, cardiovascular diseases, diabetes, and chronic respiratory diseases, the plan recognizes oral diseases as a component of the broader definition of non-communicable diseases that needs consideration. |

Note (File titles in original language):

(1) Direção Nacional de Assistência Médica - Estratégia Nacional de Saúde Oral 2019-2024

(2) Plano Estratégico Multissectorial de Prevenção e Controlo de Doenças Não Transmissíveis 2020-2029

**Reference**

1. Mozambique. Ministry of Health. Health Sector Strategic Plan 2014-2019. Available from <https://extranet.who.int/countryplanningcycles/sites/default/files/planning_cycle_repository/mozambique/mozambique_-_health_sector_strategic_plan_-_2014-2019.pdf>. Access date: May 2023.

2. República De Moçambique. Ministério Da Saúde. Direcção Nacional De Assistência Médica Estratégia Nacional de Saúde Oral 2019-2024. [Mozambique. Ministry of Health. National Directorate of Medical Assistance National Oral Health Strategy 2019-2024]. Available from <https://extranet.who.int/ncdccs/Data/MOZ_B8_ESTRATEGIA%20NACIONAL%20DE%20SAUDE%20ORAL_%202019%20-%202024%20-final%20Moz.pdf>. Access date: May 2023.

3. República De Moçambique. Ministério Da Saúde. Plano Estratégico Multissectorial de Prevenção e Controlo de Doenças Não Transmissíveis 2020-2029. [Mozambique. Ministry of Health. Strategic Plan Multisectoral of Prevention and Control of Non-Communicable Diseases 2020-2029] Available from <https://extranet.who.int/ncdccs/Data/MOZ_B3_s21_Plano%20Estrat%c3%a9gico%20Multissetorial%20de%20Prevencao%20e%20Controlo%20das%20DNTs%202020-2029%20FINALISSIMA.pdf>. Access date: May 2023.

**17. Niger**

| **No.** | **File title (sorted by the start year of each file’s targeted timeframe)** | **Inclusion of health service list (Y/N), inclusion of oral health service in the list (Y/N)** | **Related theme** | **Key findings** |
| --- | --- | --- | --- | --- |
| 1 | National Strategic Plan Multisectoral Fight Against Non-Communicable Diseases 2019-2021^(1)^ | N, N | Health facilities | - **[page 19]:** The country’s health system is organized into the central level, intermediate level, and the peripheral level. The intermediate level is responsible for monitoring the implementation of health policy and technical support and consists of 1 dental center. |
|  |  |  | Policy inclusion | - **[page 55]:** Stresses that the plan needs to collaborate with the National Program for the Fight Against Oral Diseases and Noma to ensure its implementation. |
|  |  |  | Harmful practice | - **[page 37]:** Stresses that the traditional practice of extracting milk teeth/alveoli and piercing of the gums and lips is harmful. |
| 2 | Integrated National Strategic Plan for the Fight Against Oral Diseases and Noma 2021-2025^(2)^ | N, N | Vision, objectives, strategies | - **[page 9]:** Aims to serve as a reference framework for all interventions within the framework of the promotion of oral health and the fight against oral diseases or conditions. It will also aim to strengthen the partnership between all actors in order to improve the mobilization and optimal use of resources. - **[page 32]:** Identifies four specific objectives to be achieved by the end of 2025, including (1) ensure synergy in the fight against oral diseases and noma through better coordination and collaboration; (2) offer quality preventive curative, promotional and palliative care; (3) establish a health information management mechanism related to oral diseases and noma; (4) increase financial resources for the fight against oral diseases and noma. - **[page 32]:** The plan establishes strategic axes and interventions for each objective, including (1) reinforcement of the internal coordination of the interventions of the National Program to Fight Against Oral Diseases and Noma (for objective 1); (2) increase in the supply of quality care in the fight against oral diseases and noma (for objective 2); (3) production of quality data on oral diseases and noma and their risk factors (for objective 3); (4) mobilization of financial resources for the fight against oral diseases and noma (for objective 4). |
|  |  |  | School health | - **[page 47]:** Plans to train 800 primary school teachers on the prevention and detection of oral diseases and noma. |

Note (File titles in original language):

(1) Plan Stratégique National Multisectoriel de Lutte contre les Maladies Non Transmissibles 2019-2021

(2) Plan Stratégique National Intégré de Lutte contre les Maladies Bucco-Dentaires et le Noma (PSNILMBD/N) 2021-2025

**Reference**

1. République Du Niger. Ministere De La Sante Publique. Plan Stratégique National Multisectoriel de Lutte contre les Maladies Non Transmissibles 2019-2021. [Niger. Ministry of Health. National Strategic Plan Multisectoral Fight Against Non-Communicable Diseases 2019-2021]. Available from <https://extranet.who.int/ncdccs/Data/NER_B3_s21_EXE%20-%20Doucument%20complet%20Valid%c3%a9%20PNLCMNT.pdf>. Access date: May 2023.

2. République Du Niger. Ministere De La Sante Publique. Plan Stratégique National Intégré de Lutte contre les Maladies Bucco-Dentaires et le Noma (PSNILMBD/N) 2021-2025. [Niger. Ministry of Health. Integrated National Strategic Plan for the Fight Against Oral Diseases and Noma 2021-2025]. Available from <https://extranet.who.int/ncdccs/Data/NER_B8_s21_PLAN%20Strat%c3%a9gique%20MBD-N.docx>. Access date: May 2023.

**18. North Korea**

| **No.** | **File title (sorted by the start year of each file’s targeted timeframe)** | **Inclusion of health service list (Y/N), inclusion of oral health service in the list (Y/N)** | **Related theme** | **Key findings** |
| --- | --- | --- | --- | --- |
| 1 | Medium Term Strategic Plan for the Development of the Health Sector DPR Korea 2016-2020 | N, N | Human resource | - **[page 42]:** Identifies that there are approximately 200 health care training institutions that provide training in various fields, including dental prosthesis. However, the training in dental prosthesis is outdated due to a lack of exposure to international standards and practices. |

**Reference**

1. Democratic People's Republic of Korea. Ministry of Public Health. Medium Term Strategic Plan for the Development of the Health Sector DPR Korea 2016-2020. Available from <https://extranet.who.int/countryplanningcycles/sites/default/files/planning_cycle_repository/democratic_peoples_republic_of_korea/dpr_korea_medium_term_strategic_plan_2016-20.pdf>. Access date: May 2023.

**19. Rwanda**

| **No.** | **File title (sorted by the start year of each file’s targeted timeframe)** | **Inclusion of health service list (Y/N), inclusion of oral health service in the list (Y/N)** | **Related theme** | **Key findings** |
| --- | --- | --- | --- | --- |
| 1 | National Community Health Strategic Plan 2013-2018 | N, N | Community participation | - **[page 28]:** Aims to provide guidance for the provision of holistic and sustainable quality and quantity health care services with the full participation of communities. |
|  |  |  | Human resource | - **[page 69]:** Targets to improve the capacity of community health workers to strengthen NCDs service delivery. The percentage of patients referred to health centers by community health workers for gum diseases, dental caries, oral tumors, and other oral anomalies should increase from 0% as the baseline in 2012 to 30% in 2015 and 50% in 2018. |
|  |  |  | Policy inclusion | - **[page 25]:** Plans to register people born with cleft lips and palates in all districts. |
| 2 | Rwanda Non-Communicable Diseases Policy 2015 | N, N | Disease burden | - **[page 6]:** Identifies oral disease as an emerging health problem in daily clinical consultations in Rwanda. |
| 3 | Health Service Packages for Public Health Facilities 2017 | Y, Y | Service provision | - **[page 7, 46, 154]:** Aims to describe each level of the public health system in Rwanda in terms of the types of services offered, qualifications of staff, and equipment required. Oral surgical interventions offered by facility level: - Health Center/Health Post: oral disease prevention, minor oral surgeries (simple extraction, minor soft injury repair, frenectomy). - District Hospitals: minor oral surgeries (simple extraction, minor soft injury repair, frenectomy), major oral surgeries (disimpaction, sequestrectomy, maxilla and mandibular fractures), prosthetic and orthodontics services, dental & intraoral x-ray. - National Referral Hospitals/University Teaching Hospitals/Provincial Referral Hospitals: maxillofacial trauma, endo-buccal extraction of salivary calculus, cleft lip/palate repair, alveolar periosteal or other flaps for arch closure, drainage of intra-oral abscess, minor oral surgeries (simple extraction, minor soft injury repair, frenectomy), major oral surgeries (disimpaction, sequestrectomy, maxilla and mandibular fractures), prosthetic and orthodontics services, dental & intraoral x-ray, floor of the mouth reconstruction with distant pedicled flap. |
| 4 | Fourth Health Sector Strategic Plan 2018-2024 | Y, Y | Disease burden | - **[page 48]:** Teeth and gum diseases morbidity rate at health facility level was 4% in 2016, while the target rate is 2.07% in 2020 and 1.84% in 2024. |
|  |  |  | Service provision | - **[page 49, 87-92]:** Aims to conduct massive diagnostics outreach for community NCDs, including oral and eye diseases screening. Targets of dental care provision by level of service delivery include: - Health Centers: there is need to build on the current package for Maternal and Child Care to expand the range of dental services. - District Hospitals: improve the health package of District Hospitals to provide better secondary health care, including the provision of dental services. - Provincial Hospitals: health packages for this level should include dental services in all Provincial Hospitals by 2024. - Referral Hospitals: health packages for this level should include all advanced dentistry and oral surgery. - Teaching Hospitals: health package for this level should include highly specialized and advanced dentistry and oral surgery, such as maxillofacial surgery. |
| 5 | Service Packages for Upgraded Health Centers Rwanda Health care System 2019 | Y, Y | Service provision | - **[page 3]:** Aims to define the additional or extended health care services for Medicalized Health Centers established by some isolated Districts Hospitals to reach remote areas. - **[page 5]:** The service package at the Medicalized Health Center should include dental health promotion, dental filling, dental scaling, orthodontics for dental conditions, and the provision of orthotic and prosthetic appliances. |
| 6 | National Oral Health Strategic Plan 2019-2024 | N, N | Vision, objective, strategy | - **[page 7]:** The plan aims to provide a strategic approach to prevent, treat, control and then reduce the burden of oral diseases as the most common NCDs and one of the top ten causes of morbidity in Rwanda. - **[page 20]:** Identifies six objectives, including (1) strengthen the governance and coordination of oral health within the national health system; (2) establish and strengthen preventive measures to reduce the burden of oral diseases and to raise awareness and advocacy in relation to oral diseases; (3) train the needed qualified workforce and establish retention measures for the provision of comprehensive oral health services at all levels of health care; (4) provide appropriate infrastructure, avail and equitably distribute quality equipment for oral health in Rwanda; (5) improve the quality of oral health service delivery at primary, secondary, and tertiary levels; (6) improve the monitoring and evaluation of oral health programs and interventions. Promote research in the field of oral health. - **[page 23]:** Identified several strategies and interventions for each objective, including (1) establishment and implementation of oral health coordination mechanisms at central and decentralized levels (for objective 1); (2) design and implement strategies to inform the population and decision makers about the issues of oral health and their consequences on health and wellbeing (for objective 2); (3) strengthen the capacity of the College of Medicine and Health Sciences School of Dentistry (University of Rwanda) for the production of more dentists (for objective 3); (4) design, construct, renovate, update and equip wings for oral health units in health facilities (for objective 4); (5) define and implement service packages for oral health services at all levels of the health care system (for objective 5) ; (6) define and update oral health indicators that allow to track real data on oral diseases (for objective 6). |
|  |  |  | School health | - **[page 35]:** Plans to scale-up and strengthen a comprehensive and quality oral health delivery at primary, secondary, and tertiary levels. |
| 7 | 10-year Government Program: National Strategy for Health Professionals Development 2020-2030 | N, N | Human resource | - **[page 8]:** Aims to increase the availability and coverage of high-quality services by improving the availability of a qualified, competent and equitably distributed public sector health workforce. Besides the public sector, an expanding private sector and additional potential investments may help bridge the gap toward achieving the national workforce target. - **[page 138]:** As of January 2020, Rwanda had 113 dental therapists working in the public sector. Over the next 10 years, the Ministry of Health aims to have 1,244 dental therapists in the public sector. The dental therapist training program aims to contribute to achieving 21% of this public sector workforce target (261 health workers out of 1,244). - **[page 138]:** As of January 2020, Rwanda had 5 dental surgeons working in the public sector. Over the next 10 years, the Ministry of Health aims to have 124 dental surgeons in the public sector. The dental surgery training program aims to contribute to achieving 115% of this public sector workforce target (143 health workers). |

**Reference**

1. Rwanda. Ministry of Health. National Community Health Strategic Plan 2013-2018. Available from <https://extranet.who.int/mindbank/item/7440>. Access date: May 2023.

2. Rwanda. Ministry of Health. Rwanda Non-Communicable Diseases Policy 2015. Available from <https://extranet.who.int/ncdccs/Data/RWA_B3_NCDs_Policy.2015.pdf>. Access date: May 2023.

3. Rwanda. Ministry of Health. Health Service Packages for Public Health Facilities 2017. Available from <https://www.moh.gov.rw/fileadmin/user_upload/Moh/Publications/Legal_Framework/Public_health_Facilities_service_packages_in_Rwanda-1.pdf>. Access date: May 2023.

4. Rwanda. Ministry of Health. Fourth Health Sector Strategic Plan 2018-2024. Available from <https://extranet.who.int/mindbank/item/7442>. Access date: May 2023.

5. Rwanda. Ministry of Health. Service Packages for Upgraded Health Centers Rwanda Health care System 2019. Available from <https://www.moh.gov.rw/index.php?eID=dumpFile&t=f&f=11803&token=c8fe376a7aa067259c7fe35ce5d7c6e078c74f5b>. Access date: May 2023.

6. Rwanda. Ministry of Health. National Oral Health Strategic Plan 2019-2024. Available from <https://moh.prod.risa.rw/fileadmin/user_upload/Moh/Publications/Strategic_Plan/National_Oral_Health_Strategic_Plan_2019-2024.pdf>. Access date: May 2023.

7. Rwanda. 10-year Government Program: National Strategy for Health Professionals Development 2020-2030. Available from <https://www.rbc.gov.rw/fileadmin/user_upload/strategy/RWANDA%20National%20Strategy%20for%20Health%20Professions%20Development%20%28NSHPD%202020-2030%29.pdf>. Access date: May 2023.

**20. Sierra Leone**

| **No.** | **File title (sorted by the start year of each file’s targeted timeframe)** | **Inclusion of health service list (Y/N), inclusion of oral health service in the list (Y/N)** | **Related theme** | **Key findings** |
| --- | --- | --- | --- | --- |
| 1 | Basic Package of Essential Health Services 2015-2020 | Y, Y | Service provision | - **[page 8]:** Confirms that the full package includes services on oral health. - **[page 41]:** Oral health services provided as primary care in different level of service delivery, including - Community level: education and sensitization on oral care prophylaxis (including tooth brushing). - Maternal and Child Health Post level (MCHP): same as the Community level, but plus extraction, filling and atraumatic restorative treatment (ART), ameloblastoma, minor surgery, dentures, crowns and bridges, Burkett lymphoma, and treatment of dental injuries. - Community Health Post level (CHP): same as the MCHP level. - Community Health Center (CHC) level: same as MCHP and CHP level, but plus minor surgery, ameloblastoma, and dental injuries. - **[page 49]:** Oral health services provided as secondary care in different level of service delivery, including - District Hospitals and Regional Hospitals level: extraction, filling and ART, ameloblastoma, Mir surgery, dentures, crowns and bridges, Burkett lymphoma, treatment of dental injuries, and mobile clinic. |
|  |  |  | School health | - **[page 24]:** The essential school health package consists of child health services in pre-primary, primary, and secondary schools, including oral screening and care twice per year. |
| 2 | Non-Communicable Disease (NCDs) Strategic Plan 2020-2024 | N, N | Disease burden | - **[page 68]:** Identifies that 68% of the 2017 population has oral disorders. |

**Reference**

1. Sierra Leone. Ministry of Health and Sanitation. Basic Package of Essential Health Services 2015-2020. Available from <https://mohs2017.files.wordpress.com/2017/06/gosl_2015_basic-package-of-essential-health-services-2015-2020.pdf>. Access date: May 2023.

2. Sierra Leone. Ministry of Health and Sanitation. Non-Communicable Disease (NCDs) Strategic Plan 2020-2024. Available from <https://extranet.who.int/ncdccs/Data/SLE_B3_s21_NCD%20strategic%20plan%202020-2024%2023Feb2020%20FINAL%20signed%20CF%20(1).docx>. Access date: May 2023.

**21. Somalia**

| **No.** | **File title (sorted by the start year of each file’s targeted timeframe)** | **Inclusion of health service list (Y/N), inclusion of oral health service in the list (Y/N)** | **Related theme** | **Key findings** |
| --- | --- | --- | --- | --- |
| 1 | Essential Package of Health Services 2020 | Y, Y | Service provision | - **[page 75]:** Recommends a standardized set of oral health interventions in different level of service delivery, including - Community level: oral health promotion. - Primary Health Unit level: referral of dental problems. - Health Center level: oral health promotion and treatment, dental extraction, drainage of dental abscess, referral of dental problems. - District Hospital level: dental extraction, drainage of dental abscess, manage dental emergencies. - No oral health service mentioned at regional/national hospitals. |

**Reference**

1. Somalia. Ministry of Health and Human Services. Essential Package of Health Services 2020. Available from <https://reliefweb.int/attachments/981c3ca3-6914-3d40-824a-bac5889d906c/somalia_ephs_web.pdf>. Access date: May 2023.

**22. South Sudan**

| **No.** | **File title (sorted by the start year of each file’s targeted timeframe)** | **Inclusion of health service list (Y/N), inclusion of oral health service in the list (Y/N)** | **Related theme** | **Key findings** |
| --- | --- | --- | --- | --- |
| 1 | National Health Policy 2016-2026 | N, N | Policy inclusion | - **[page 22]:** The government commits to developing and integrating oral health into all levels of packages of care (primary, secondary, and tertiary) to ensure reduction of mortality and morbidity due to NCDs. |

**Reference**

1. South Sudan. Ministry of Health. National Health Policy 2016-2026. Available from <https://extranet.who.int/countryplanningcycles/sites/default/files/planning_cycle_repository/south_sudan/south_sudan_national_health_policy_2016_to_2025_2.pdf>. Access date: May 2023.

**23. Sudan**

| **No.** | **File title (sorted by the start year of each file’s targeted timeframe)** | **Inclusion of health service list (Y/N), inclusion of oral health service in the list (Y/N)** | **Related theme** | **Key findings** |
| --- | --- | --- | --- | --- |
| 1 | National Health Sector Strategic Plan Ⅱ 2012-2016 | Y, Y | Service provision | - **[page 47]:** Recommends a minimum primary health care package to prioritize expansion and equity in the utilization of primary health care services. The package will be adapted according to local health conditions and capacity. The minimum package should provide basic dental care service at the Family Health Center level. |

**Reference**

1. Sudan. National Health Sector Strategic Plan Ⅱ 2012-2016. Available from <https://extranet.who.int/countryplanningcycles/sites/default/files/planning_cycle_repository/sudan/sudan_national_health_sector_strategic_plan_nhssp_2012-2016.pdf>. Access date: May 2023.

**24. Syrian Arab Republic**

No national health policy documents with oral health care information were found.

**25. Togo**

| **No.** | **File title (sorted by the start year of each file’s targeted timeframe)** | **Inclusion of health service list (Y/N), inclusion of oral health service in the list (Y/N)** | **Related theme** | **Key findings** |
| --- | --- | --- | --- | --- |
| 1 | National Health Development Plan 2017-2022^(1)^ | N, N | Policy inclusion | - **[page 12]:** Includes an objective on combating NCDs, including oral health, to achieve the vision that the highest possible level of health is provided to the population through the development of a high-performing health system. |
|  |  |  | Human resource | - **[page 61]:** The needs for human resources for health were estimated based on the available workforce as of December 31, 2015. Projections of the required workforce between 2017 and 2022 were made. There were 18 dental surgeons in 2015, while the needs for additional dental surgeons were 4 for 2017, 3 for 2018, 4 for 2019, 4 for 2020, 3 for 2021, and 4 for 2022. |
| 2 | Policy and Multisectoral Strategic Plan for the Prevention and Control of Non-Communicable Diseases 2018-2022^(2)^ | N, N | Policy inclusion | - **[page 50, 128]:** Includes the promotion of the fight against oral diseases including noma as an intervention area to achieve the strategic objective aiming to strengthen capacities for the management of NCDs within the framework of primary health care. |
|  |  |  | Human resource | - **[page 19]:** Estimates the situation of public and private sector health personnel by as of December 31, 2017: there were 56 dental surgeons (48 in public sector and 8 in private sector). |

Note (File titles in original language):

(1) Plan National de Developpment Sanitaire 2017-2022

(2) Politique et Plan Stratégique Multisectoriel de Lutte contre les Maladies Non Transmissibles 2018-2022

**Reference**

1. République Togolaise. Ministere De La Sante Et De La Protection Sociale. Plan National de Developpment Sanitaire 2017-2022. [Togo. Ministry of Health and Social Protection. National Health Development Plan 2017-2022] Available from <https://extranet.who.int/countryplanningcycles/sites/default/files/planning_cycle_repository/togo/togo_pnds_2017-2022_version_definitive_210217_en_edition.pdf>. Access date: May 2023.

2. République Togolaise. Ministere De La Sante Et De L’Hygiene Publique. Politique et Plan Stratégique Multisectoriel de Lutte contre les Maladies Non Transmissibles 2018-2022 [Togo. Ministry of Health and Public Hygiene. Policy and Multisectoral Strategic Plan for the Prevention and Control of Non-Communicable Diseases 2018-2022]. Available from <https://extranet.who.int/countryplanningcycles/sites/default/files/planning_cycle_repository/togo/togo_pnds_2017-2022_version_definitive_210217_en_edition.pdf>. Access date: May 2023.

**26. Uganda**

| **No.** | **File title (sorted by the start year of each file’s targeted timeframe)** | **Inclusion of health service list (Y/N), inclusion of oral health service in the list (Y/N)** | **Related theme** | **Key findings** |
| --- | --- | --- | --- | --- |
| 1 | Health Sector Development Plan 2015-2020 | N, N | Policy inclusion | - **[page 66]:** Plans to strengthen service delivery systems through the improvement of specialized services delivery, including oral surgery and care. |
| 2 | National Oral Health Policy 2007 | N, N | Policy inclusion | - **[page 9]:** The goal of the policy is to improve the oral health of Ugandans in order to promote a healthy and productive life. - **[page 9]:** Objectives: (1) To provide guidelines for oral health managers and service providers that define national oral health programmes; (2) to provide guidelines for oral health managers and service providers that facilitate population wide initiatives to promote oral health; (3) to provide guidelines that assist oral health managers and service providers customize locally effective oral health strategies; (4) to provide a framework for monitoring and evaluating the effectiveness of strategies taken to improve oral health and sustain an ongoing process of policy review and development. |

**Reference**

1. Uganda. Ministry of Health. Health Sector Development Plan 2015-2020. Available from <https://extranet.who.int/countryplanningcycles/sites/default/files/planning_cycle_repository/uganda/health_sector_development_plan_2015-16_2019-20_0.pdf>. Access date: May 2023.

2. Uganda. Ministry of Health. National Oral Health Policy. Available from <https://extranet.who.int/ncdccs/Data/UGA_B8_National%20Oral%20Health%20Policy.pdf>. Access date: May 2023.

**27. Yemen**

| **No.** | **File title (sorted by the start year of each file’s targeted timeframe)** | **Inclusion of health service list (Y/N), inclusion of oral health service in the list (Y/N)** | **Related theme** | **Key findings** |
| --- | --- | --- | --- | --- |
| 1 | National Health Strategy 2010-2025 | N, N | Policy inclusion | - **[page 58]:** Identifies dentistry as a primary health care service that should be provided to the public. |

**Reference**

1. Yemen. Ministry of Public Health & Population. National Health Strategy 2010-2025. Available from <https://extranet.who.int/countryplanningcycles/sites/default/files/planning_cycle_repository/yemen/nat_health_strategy_-_yemen_eng.pdf>. Access date: May 2023.

**Web Appendix 8. Oral health care keyword counts in National Health Policies, Strategies, and Plans (NHPSPs)**

| **Country** | **Total mentions** | **Dental^1^** | **Oral** | **Dentist** | **Fluoride** | **Cavity^2^** | **School** | **Extract** | **Cancer** | **Gum** | **Dentures^3^** | **Lip** | **Maxilla** | **Mouth** | **Toothpaste** |
| --- | --- | --- | --- | --- | --- | --- | --- | --- | --- | --- | --- | --- | --- | --- | --- |
| Madagascar | 32 | 18 | 6 | 1 | 0 | 3 | 1 | 3 | 0 | 0 | 0 | 0 | 0 | 0 | 0 |
| Mozambique | 25 | 3 | 14 | 0 | 4 | 0 | 2 | 0 | 0 | 0 | 0 | 0 | 0 | 1 | 1 |
| Rwanda | 14 | 6 | 3 | 2 | 0 | 0 | 0 | 0 | 0 | 2 | 0 | 0 | 1 | 0 | 0 |
| Eritrea | 10 | 10 | 0 | 0 | 0 | 0 | 0 | 0 | 0 | 0 | 0 | 0 | 0 | 0 | 0 |
| Malawi | 10 | 0 | 4 | 0 | 2 | 1 | 2 | 0 | 0 | 0 | 0 | 1 | 0 | 0 | 0 |
| Gambia | 9 | 9 | 0 | 0 | 0 | 0 | 0 | 0 | 0 | 0 | 0 | 0 | 0 | 0 | 0 |
| Guinea | 7 | 2 | 2 | 0 | 0 | 1 | 0 | 0 | 2 | 0 | 0 | 0 | 0 | 0 | 0 |
| CAF* | 5 | 4 | 0 | 1 | 0 | 0 | 0 | 0 | 0 | 0 | 0 | 0 | 0 | 0 | 0 |
| Burundi | 4 | 4 | 0 | 0 | 0 | 0 | 0 | 0 | 0 | 0 | 0 | 0 | 0 | 0 | 0 |
| Togo | 4 | 3 | 0 | 1 | 0 | 0 | 0 | 0 | 0 | 0 | 0 | 0 | 0 | 0 | 0 |
| Yemen | 4 | 2 | 0 | 2 | 0 | 0 | 0 | 0 | 0 | 0 | 0 | 0 | 0 | 0 | 0 |
| North Korea | 3 | 1 | 0 | 1 | 0 | 0 | 0 | 0 | 0 | 0 | 1 | 0 | 0 | 0 | 0 |
| Mali | 2 | 2 | 0 | 0 | 0 | 0 | 0 | 0 | 0 | 0 | 0 | 0 | 0 | 0 | 0 |
| Ethiopia | 1 | 0 | 1 | 0 | 0 | 0 | 0 | 0 | 0 | 0 | 0 | 0 | 0 | 0 | 0 |
| South Sudan | 1 | 0 | 1 | 0 | 0 | 0 | 0 | 0 | 0 | 0 | 0 | 0 | 0 | 0 | 0 |
| Sudan | 1 | 1 | 0 | 0 | 0 | 0 | 0 | 0 | 0 | 0 | 0 | 0 | 0 | 0 | 0 |
| Uganda | 1 | 0 | 1 | 0 | 0 | 0 | 0 | 0 | 0 | 0 | 0 | 0 | 0 | 0 | 0 |
| **Sum** | **133** | **65** | **32** | **8** | **6** | **5** | **5** | **3** | **2** | **2** | **1** | **1** | **1** | **1** | **1** |

Note:

1. Dental/tooth

2. Cavity/caries/decay

3. Dentures/dental prosthesis

4. Oral health care keywords listed in Web Appendix 5 but not included in this table were those that did not appear in any accessible NHPSPs.

5. Except for the Syrian Arab Republic, each of the rest 26 low-income countries was successfully identified with an accessible NHPSP. Nine countries, including Afghanistan, Burkina Faso, Chad, Congo DR, Guinea-Bissau, Liberia, Niger, Sierra Leone, and Somalia, did not include any oral health keywords in their NHPSPs. Meanwhile, seventeen countries (listed in the table) aggregately mentioned keywords 133 times in their NHPSPs. Except for the most frequently mentioned keywords such as “oral” and “dental”, six countries, including the Central African Republic, Madagascar, North Korea, Rwanda, Togo, and Yemen, slightly mentioned the current stock of oral health professionals or their outdated models of workforce training in their NHPSPs. In addition, only three countries, Madagascar, Malawi, and Mozambique, mentioned oral health promotion and oral disease prevention in a public health approach, represented by an expanded usage of fluoride among their general population and school children.

*. Central African Republic

|  | Frequency of mentions equal to or larger than 5 |
| --- | --- |
|  | Frequency of mentions less than 5 but not equal to zero |

**Web Appendix 9. Oral health care keyword counts in National Non-communicable Disease Strategic Plans**

| **Country** | **Total mentions** | **Dental^1^** | **Noma** | **Oral** | **Dentist** | **Cavity^2^** | **Maxilla** | **School** | **Mouth** | **Periodontal^3^** | **Extract** | **Lip** | **Pain** | **Stomatology^4^** | **Trauma** |
| --- | --- | --- | --- | --- | --- | --- | --- | --- | --- | --- | --- | --- | --- | --- | --- |
| Burkina Faso | 58 | 31 | 4 | 1 | 6 | 2 | 6 | 3 | 1 | 1 | 0 | 0 | 1 | 1 | 1 |
| Chad | 33 | 14 | 18 | 0 | 0 | 0 | 0 | 0 | 0 | 1 | 0 | 0 | 0 | 0 | 0 |
| Madagascar | 20 | 12 | 0 | 1 | 3 | 4 | 0 | 0 | 0 | 0 | 0 | 0 | 0 | 0 | 0 |
| Togo | 20 | 12 | 4 | 1 | 2 | 1 | 0 | 0 | 0 | 0 | 0 | 0 | 0 | 0 | 0 |
| Niger | 12 | 6 | 2 | 1 | 0 | 1 | 0 | 0 | 0 | 0 | 1 | 1 | 0 | 0 | 0 |
| CAF* | 5 | 5 | 0 | 0 | 0 | 0 | 0 | 0 | 0 | 0 | 0 | 0 | 0 | 0 | 0 |
| Ethiopia | 4 | 2 | 0 | 2 | 0 | 0 | 0 | 0 | 0 | 0 | 0 | 0 | 0 | 0 | 0 |
| Sierra Leone | 4 | 1 | 0 | 2 | 0 | 0 | 0 | 0 | 1 | 0 | 0 | 0 | 0 | 0 | 0 |
| Gambia | 3 | 0 | 0 | 3 | 0 | 0 | 0 | 0 | 0 | 0 | 0 | 0 | 0 | 0 | 0 |
| Rwanda | 3 | 0 | 0 | 3 | 0 | 0 | 0 | 0 | 0 | 0 | 0 | 0 | 0 | 0 | 0 |
| Burundi | 2 | 2 | 0 | 0 | 0 | 0 | 0 | 0 | 0 | 0 | 0 | 0 | 0 | 0 | 0 |
| Mozambique | 1 | 0 | 0 | 1 | 0 | 0 | 0 | 0 | 0 | 0 | 0 | 0 | 0 | 0 | 0 |
| **Sum** | **165** | **85** | **28** | **15** | **11** | **8** | **6** | **3** | **2** | **2** | **1** | **1** | **1** | **1** | **1** |

Note:

1. Dental/tooth

2. Cavity/caries/decay

3. Periodontal/gingivitis

4. Stomatology/odontology

5. Oral health care keywords listed in Web Appendix 5 but not included in this table were those that did not appear in any accessible Non-communicable Disease Strategic Plans.

6. We identified National Non-communicable Disease Strategic Plans for 22 countries, ten of which did not include any oral health keywords, including Afghanistan, Congo DR, Eritrea, Guinea, Malawi, Mali, North Korea, South Sudan, Sudan, and Uganda, while the other twelve countries mentioned oral health keywords with an aggregate of 165 times. Only six countries mentioned oral health keywords at least five times in their National Non-communicable Disease Strategic Plans. They are Burkina Faso, Chad, Madagascar, Togo, Niger, and the Central African Republic. We did not find any National Non-communicable Disease Strategic Plans for Guinea-Bissau, Liberia, Somalia, the Syrian Arab Republic, and Yemen. Of particular concern, the highly fatal gangrenous oral disease “noma” was only mentioned by four countries – significantly by Chad and slightly by Burkina Faso, Togo, and Niger. Only Burkina Faso established a public health approach that aims to increase the proportion of primary and secondary schools where students have been sensitized about oral health.

*. Central African Republic

|  | Frequency of mentions equal to or larger than 5 |
| --- | --- |
|  | Frequency of mentions less than 5 but not equal to zero |

**Web Appendix 10. Annual amount of per capita expenditure on dental outpatient care by financing sectors (2020 US dollar)**


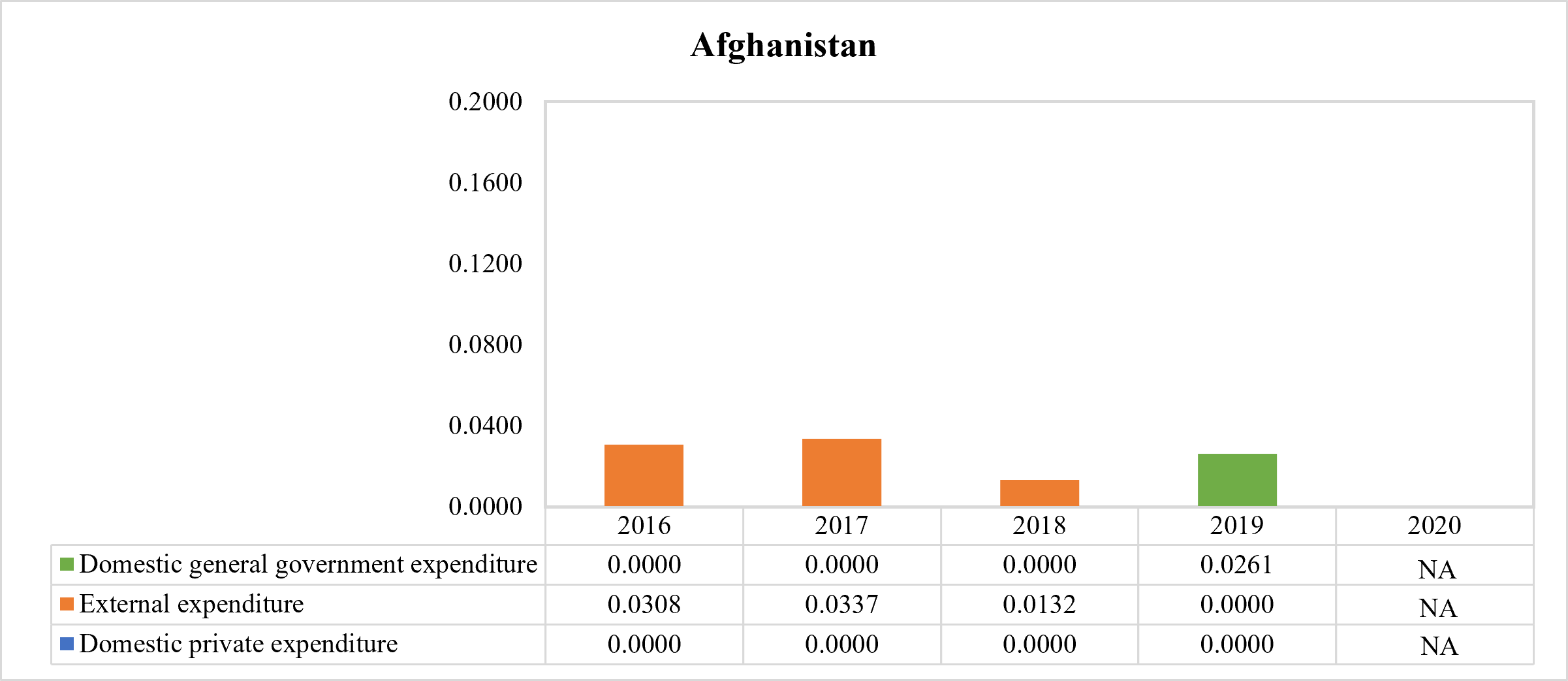


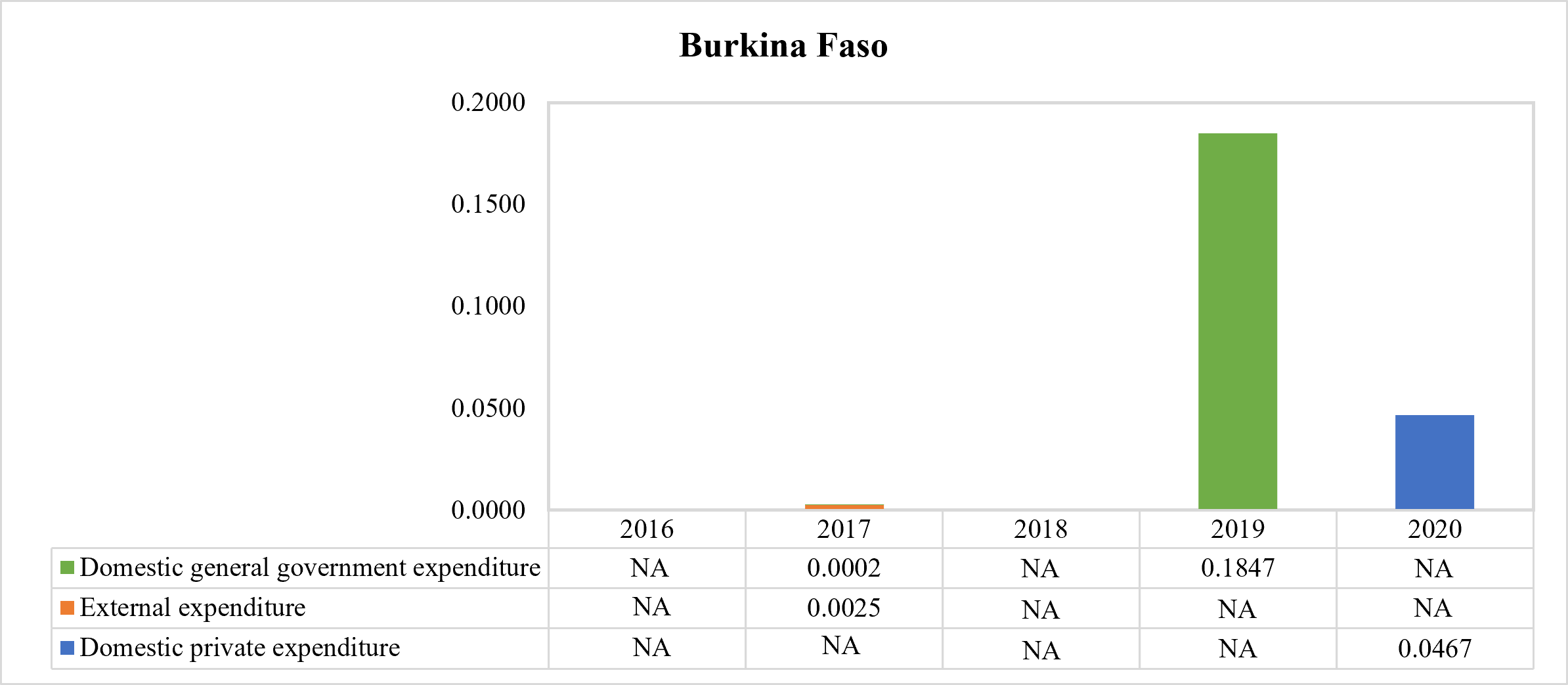


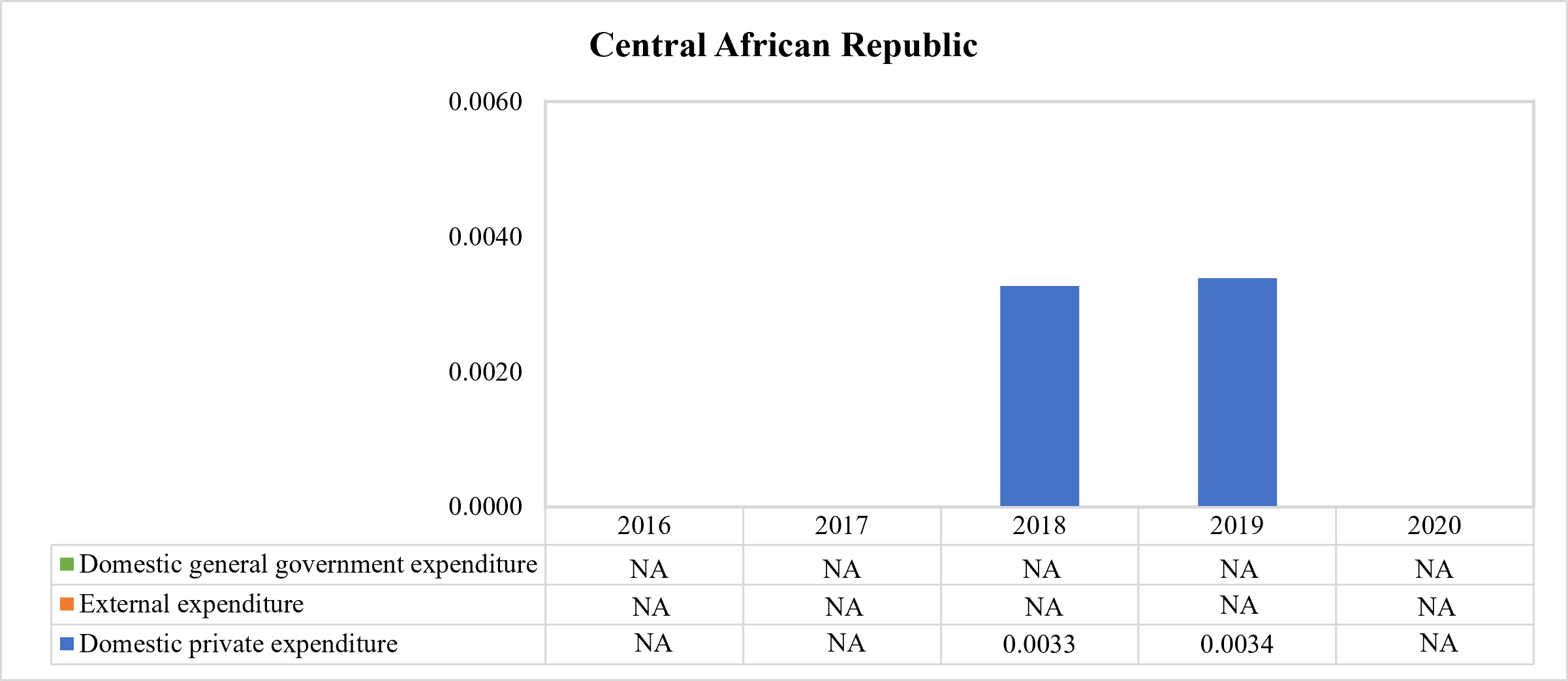


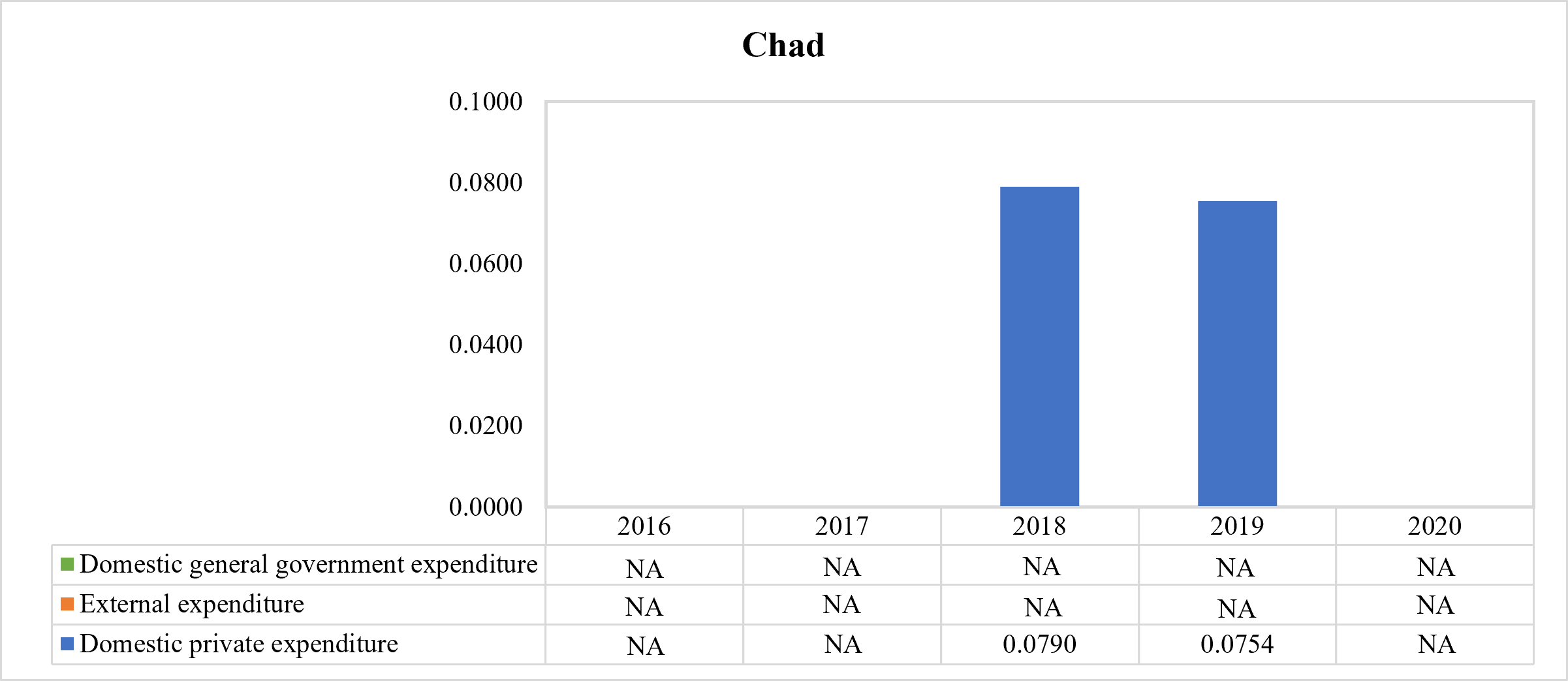


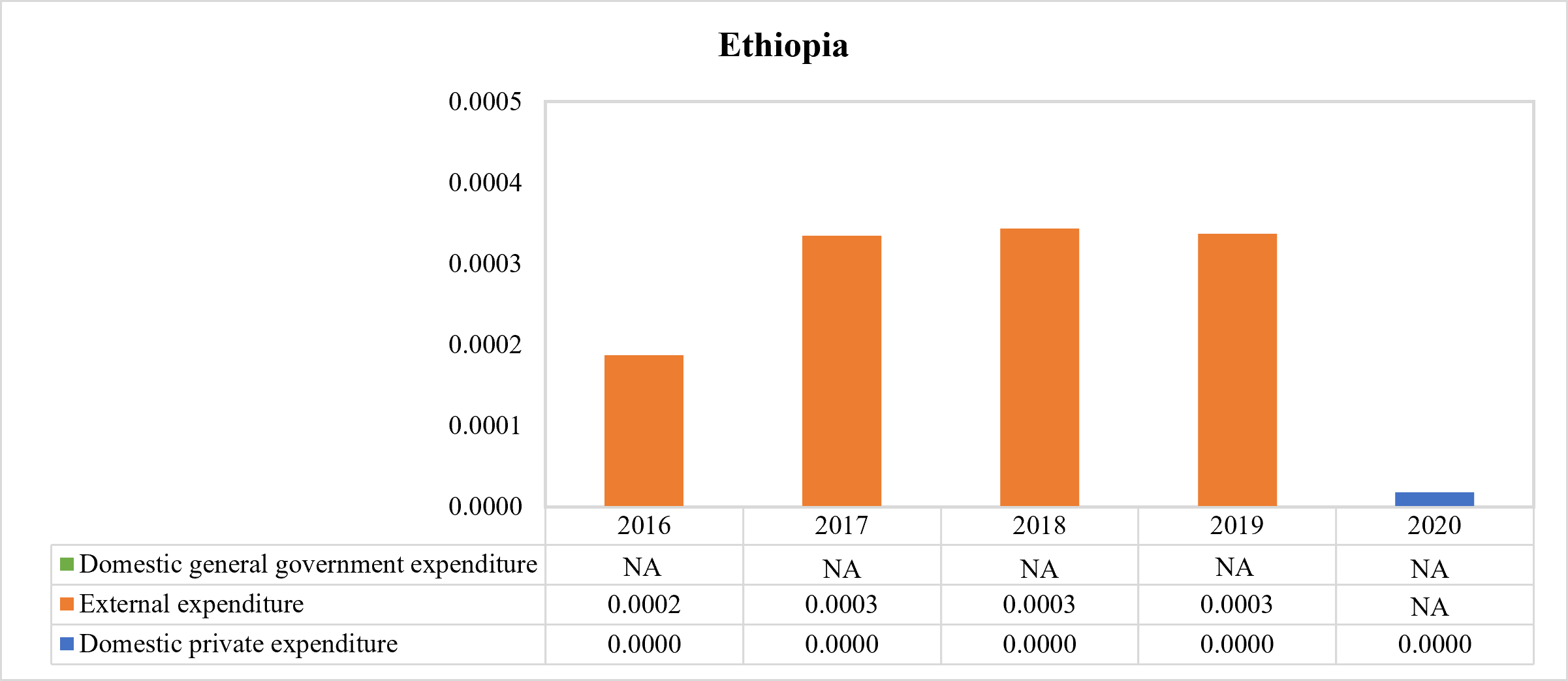


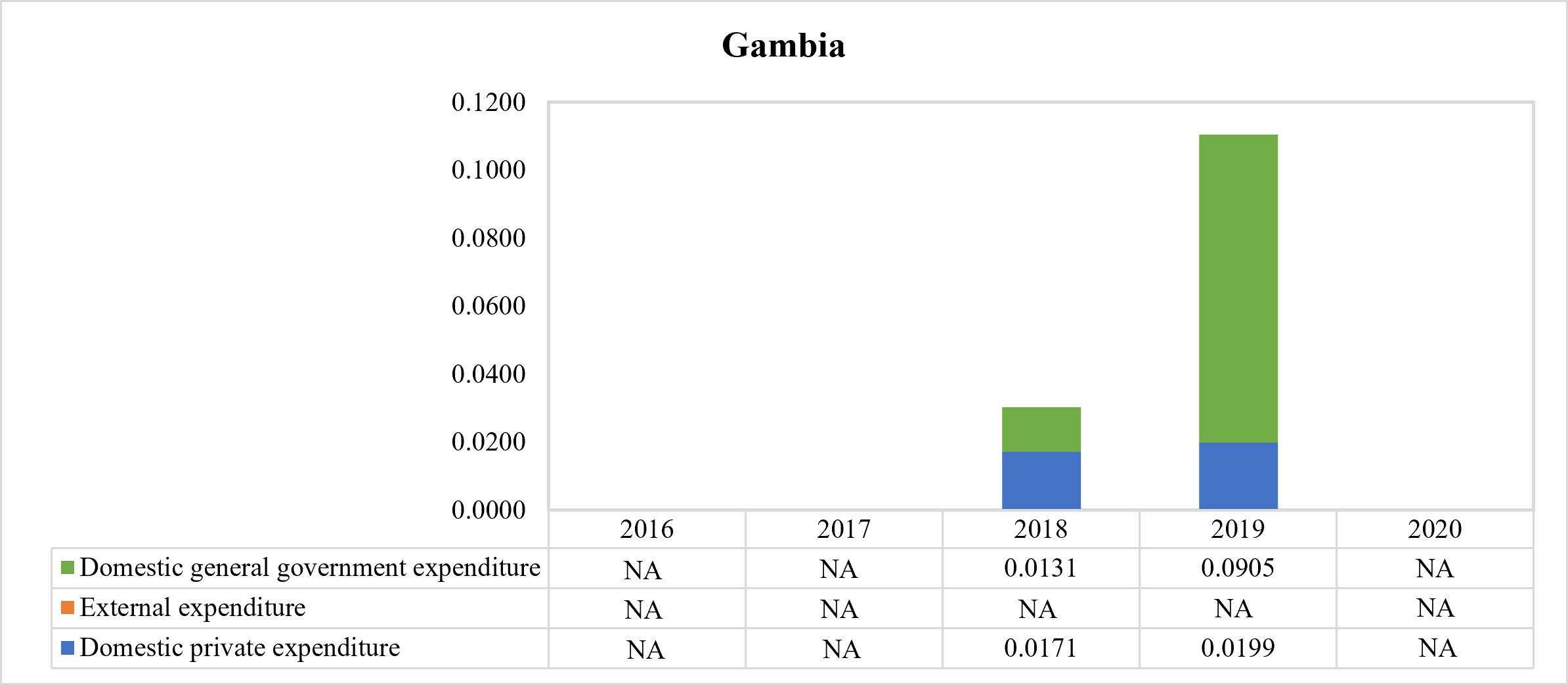


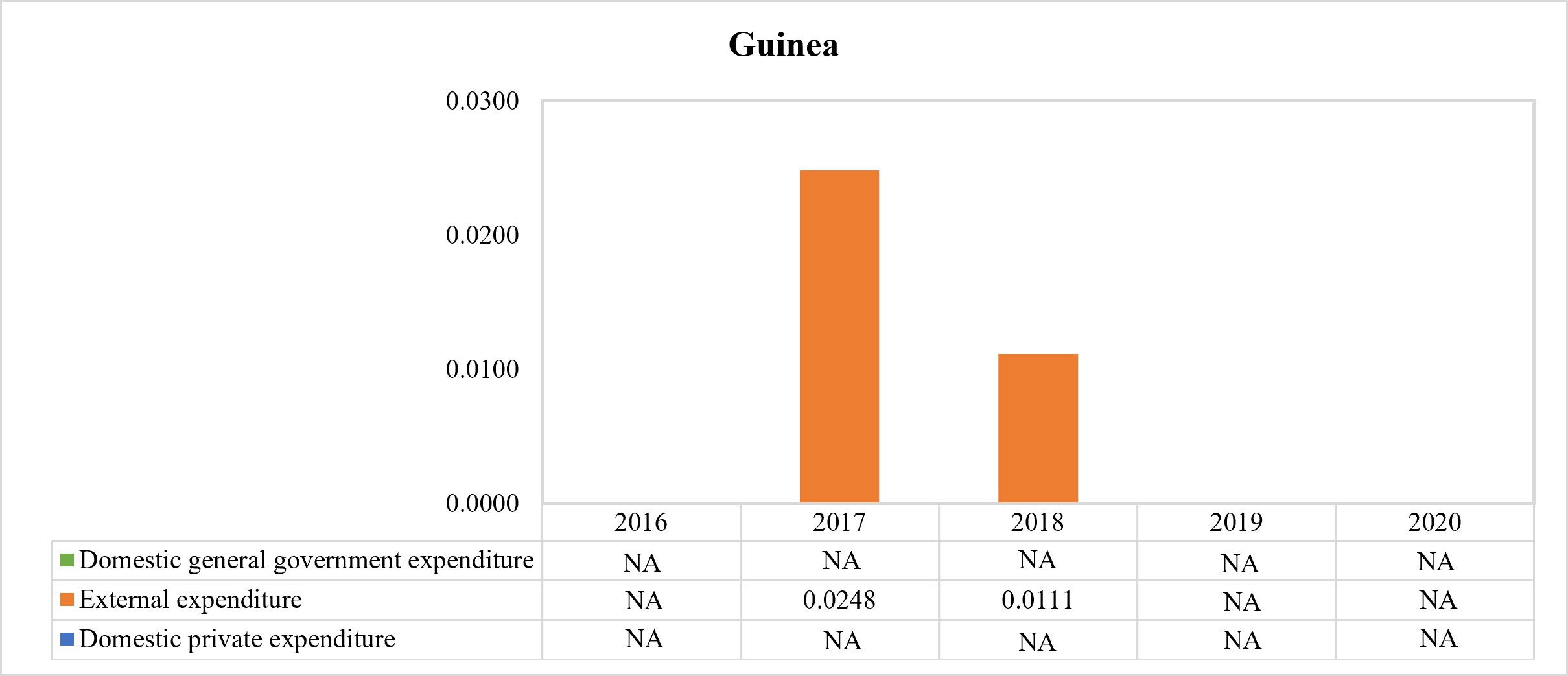


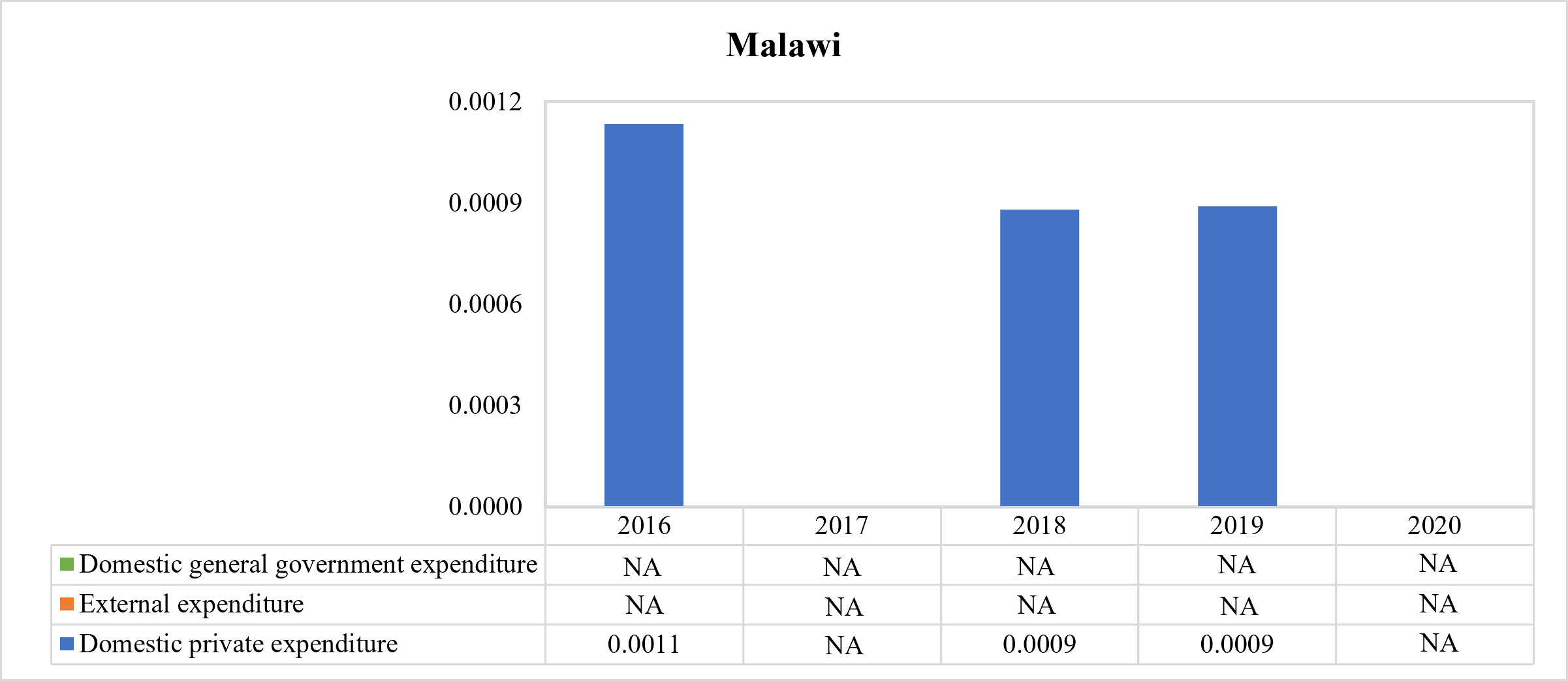


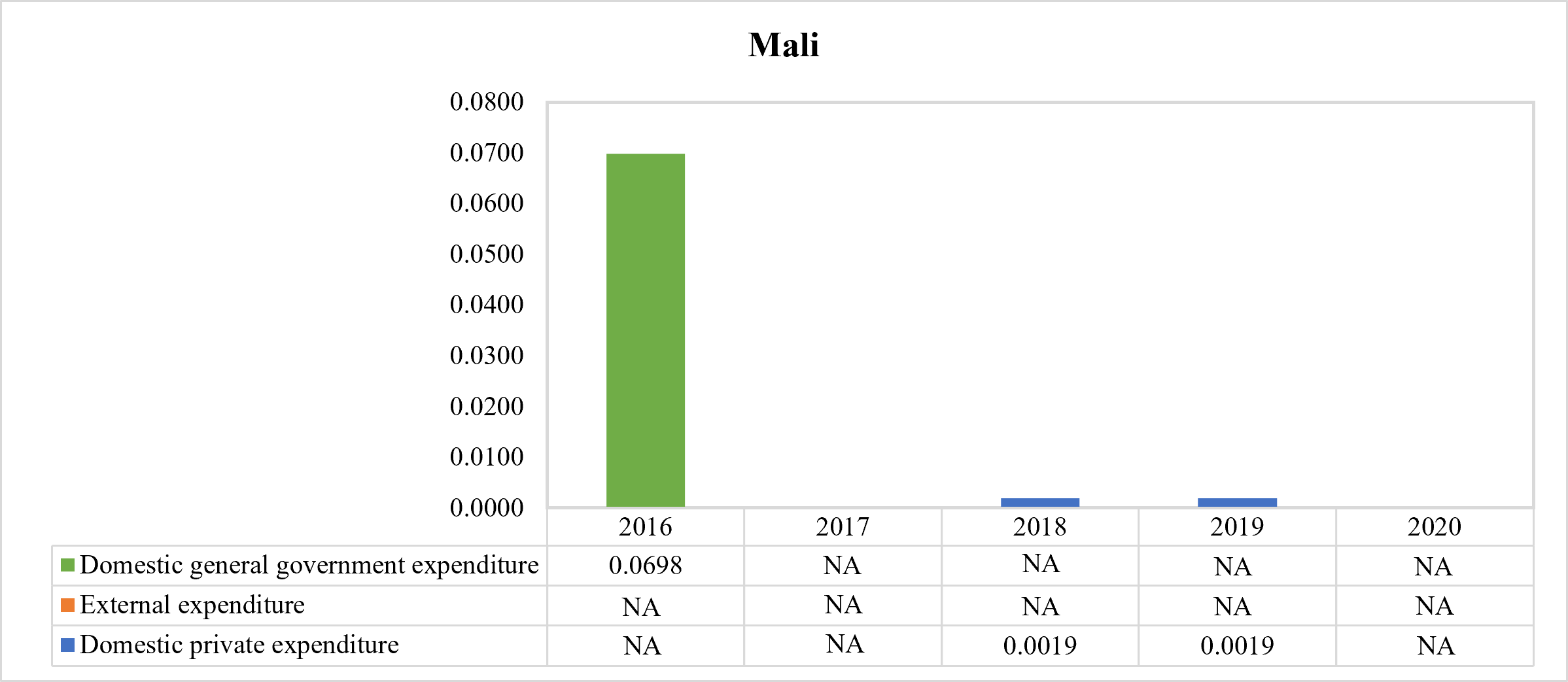


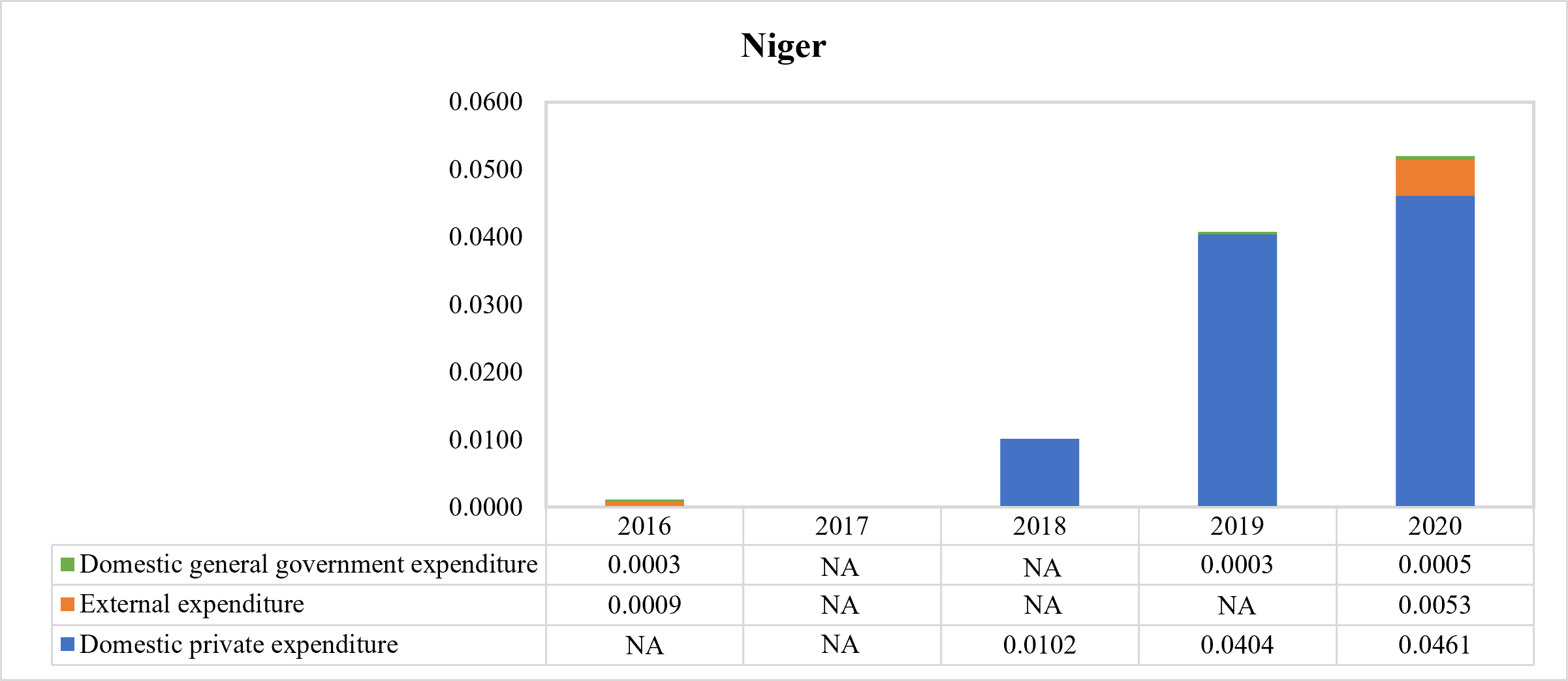


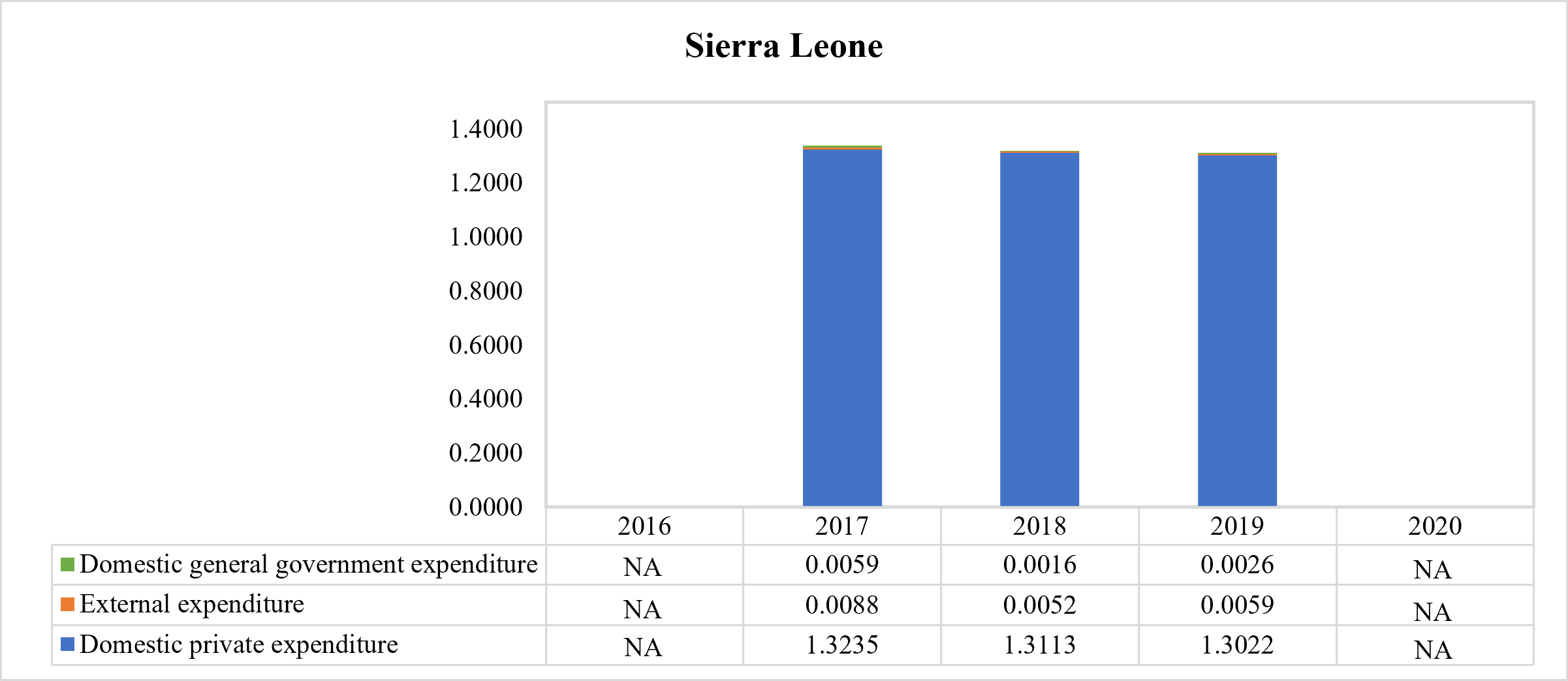


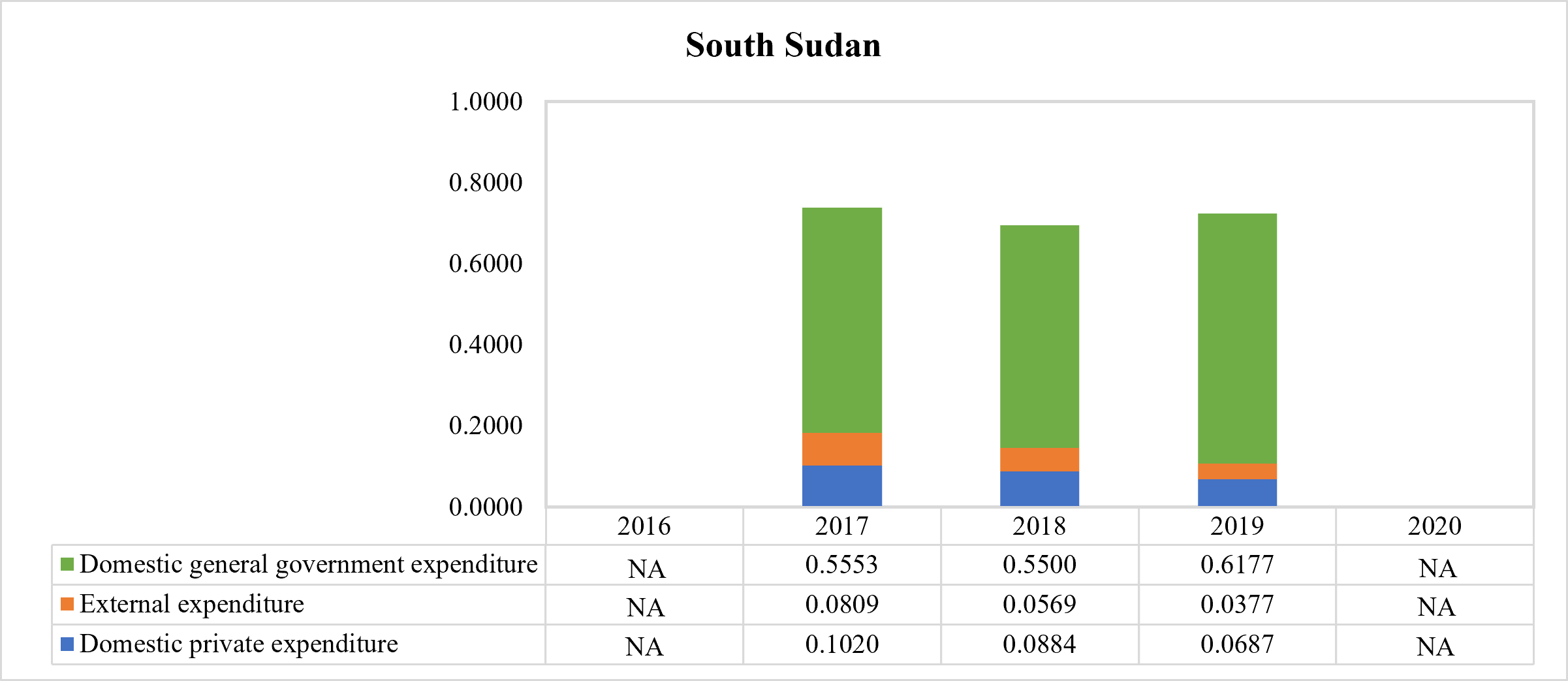


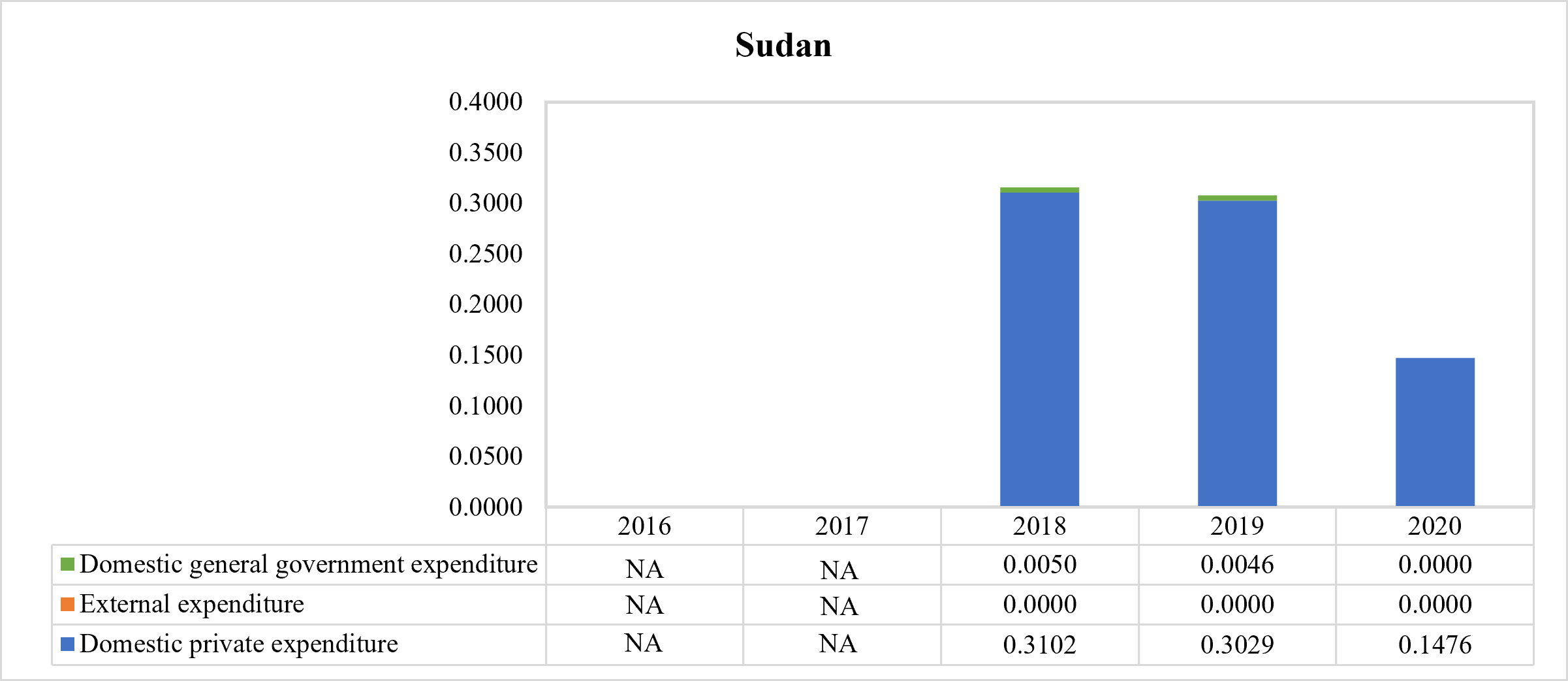


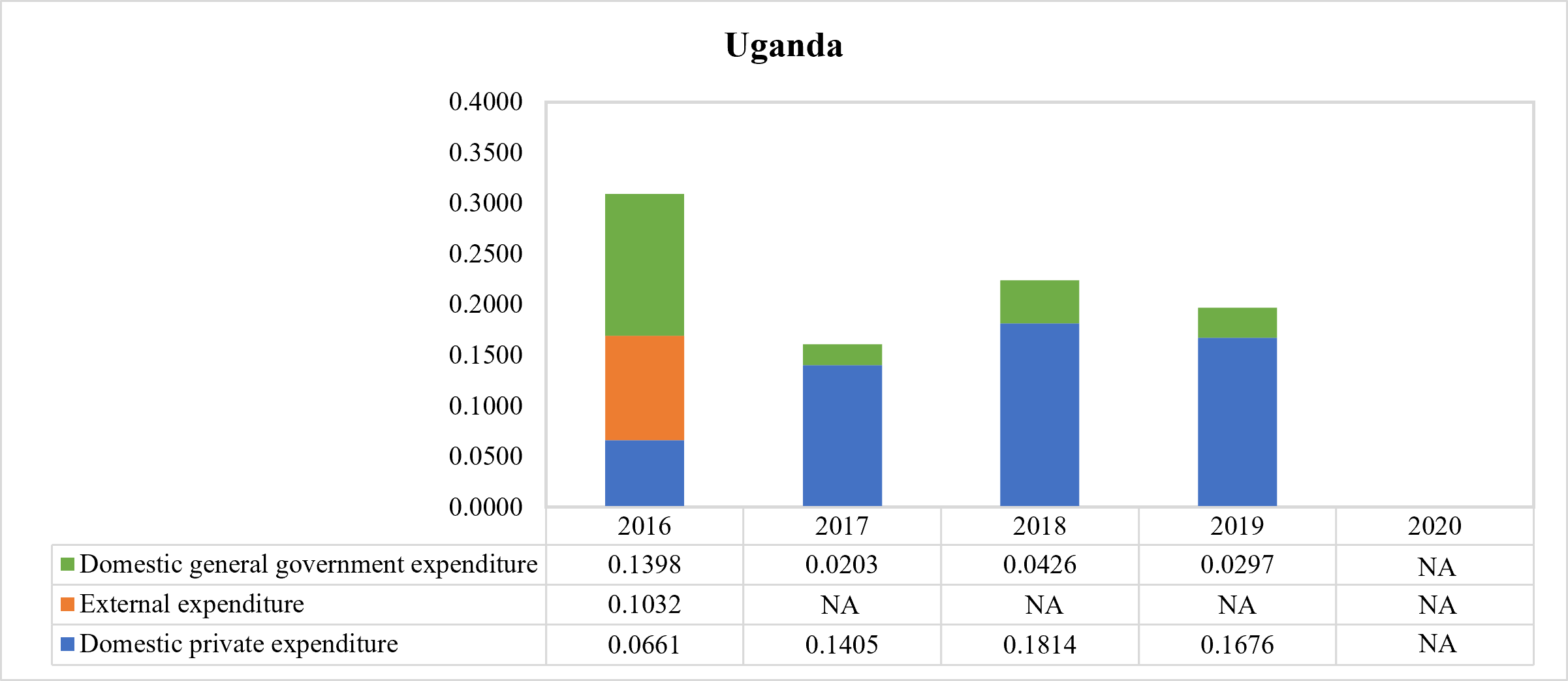


(Data source: WHO Global Health Expenditure Database)

**Web Appendix 11. Comparison of oral health care expenditure for countries with available data in 2019 from two data sources**

*CAR: Central African Republic

(Data source: WHO Global Health Expenditure Database, WHO Global Oral Health Status Report Country Profile)

**Web Appendix 12. Summary of findings of the five study aspects**

| **Country** | **Policy integration** | **Covered oral health services** | **Utilization rate** | **Per capita expenditure on oral outpatient care (US$)** | | **Most recent density of oral health care professionals per 10,000 population (Data source: National Health Workforce Account^68^)** |
| --- | --- | --- | --- | --- | --- | --- |
|  |  |  |  | **Most recent expenditure by financing sector (Data source: Global Health Expenditure Database^66^)** | **Expenditure in 2019 (Data source: Global Oral Health Status Report^67^)** |  |
| Afghanistan | Partial integration^1-5^ | Maxillofacial trauma treatment, gum disease and treatment, salivary disease treatment^1^ | No data | - Domestic general government expenditure: US$ 0.026 - Domestic private expenditure: US$ 0.000 - External expenditure: 0.000 | US$ 0.5 | - Dentists: 0.71 - Dental prosthetic technicians: 0.14 - Dental assistants & therapists: 0.17 |
| Burkina Faso | Full integration^6-8^ | No data | Only 2.1% (95% CI 1.7-2.6) of the nationally surveyed participants visited a dentist in the past 6 months^57^ | - Domestic general government expenditure: no data - Domestic private expenditure: US$ 0.047 - External expenditure: no data | US$ 0.2 | - Dentists: 0.02 - Dental prosthetic technicians: <0.01 - Dental assistants & therapists: 0.07 |
| Burundi | Partial integration^9-11^ | No data | No data | No data | US$ 0.1 | - Dentists: 0.01 - Dental prosthetic technicians: no data - Dental assistants & therapists: no data |
| Central African Republic | Partial integration^12,13^ | No data | No data | - Domestic general government expenditure: no data - Domestic private expenditure: US$ 0.003 - External expenditure: no data | US$ 0.2 | - Dentists: 0.02 - Dental prosthetic technicians: <0.01 - Dental assistants & therapists: no data |
| Chad | Partial integration^14^ | No data | No data | - Domestic general government expenditure: no data - Domestic private expenditure: US$ 0.075 - External expenditure: no data | US$ 0.3 | - Dentists: 0.01 - Dental prosthetic technicians: <0.01 - Dental assistants & therapists: 0.01 |
| Congo DR | Full integration^15^ | No data | No data | No data | US$ 0.2 | - Dentists: 0.05 - Dental prosthetic technicians: no data - Dental assistants & therapists: no data |
| Eritrea | Partial integration^16,17^ | School oral health service, oral health service for the elderly*^17^ | No data | No data | US$ 0.4 | - Dentists: 0.54 - Dental prosthetic technicians: no data - Dental assistants & therapists: no data |
| Ethiopia | Partial integration^18-20^ | Tooth extraction, dental abscess drainage, dental caries treatment, cleft lip and palate repair, maxillofacial trauma treatment, oral health knowledge awareness^†^, oral tumor treatment, dental trauma treatment, gum disease and treatment, oral emergency service, orofacial infection treatment^19^ | - 92.6% of randomly surveyed school-age children (6-12 years old) in Areka Town, Ethiopia, have never visited a dental clinic during the past year^58^ - The overall dental service utilization among the surveyed school-age children (6-15 years old) in Mekelle city, Ethiopia, was 10.6% (95% CI 7.5-13.6) in the past year, and 89.4% of the children had never seen a dentist^59^ | - Domestic general government expenditure: no data - Domestic private expenditure: US$ 0.000 - External expenditure: no data | US$ 0.0 | - Dentists: 0.02 - Dental prosthetic technicians: no data - Dental assistants & therapists: 0.01 |
| Gambia | Partial integration^21-23^ | Specialized dental care services*^22^ | No data | - Domestic general government expenditure: US$ 0.091 - Domestic private expenditure: US$ 0.020 - External expenditure: no data | US$ 0.01 | - Dentists: 0.03 - Dental prosthetic technicians: 0.01 - Dental assistants & therapists: 0.08 |
| Guinea | Partial integration^24^ | No data | No data | - Domestic general government expenditure: no data - Domestic private expenditure: no data - External expenditure: 0.011 | US$ 0.2 | - Dentists: 0.06 - Dental prosthetic technicians: no data - Dental assistants & therapists: no data |
| Guinea-Bissau | Full integration^25^ | No data | No data | No data | US$ 0.3 | - Dentists: 0.01 - Dental prosthetic technicians: 0.01 - Dental assistants & therapists: 0.1 |
| Liberia | Partial integration^26^ | Tooth extraction, dental abscess drainage, dental caries treatment, cleft lip and palate repair, school oral health service^26^ | No data | No data | US$ 0.2 | - Dentists: 0.01 - Dental prosthetic technicians: no data - Dental assistants & therapists: 0.03 |
| Madagascar | Full integration^27-30^ | Tooth extraction, dental caries treatment, dentures and prosthesis, oral tumor treatment^28^ | No data | No data | US$ 0.2 | - Dentists: 0.21 - Dental prosthetic technicians: <0.01 - Dental assistants & therapists: 0.01 |
| Malawi | Partial integration^31^ | Tooth extraction, dental filling, specialized dental care services*^31^ | No data | - Domestic general government expenditure: no data - Domestic private expenditure: US$ 0.001 - External expenditure: no data | US$ 0.1 | - Dentists: 0.08 - Dental prosthetic technicians: no data - Dental assistants & therapists: 0.11 |
| Mali | Full integration^32,33^ | No data | No data | - Domestic general government expenditure: no data - Domestic private expenditure: US$ 0.002 - External expenditure: no data | US$ 0.1 | - Dentists: 0.02 - Dental prosthetic technicians: 0.01 - Dental assistants & therapists: 0.03 |
| Mozambique | Full integration^34-36^ | Oral health knowledge awareness^†^, school oral health service, oral disease preventive service^†^*, fluoride use expansion^†34^ | No data | No data | US$ 0.2 | - Dentists: 0.11 - Dental prosthetic technicians: 0.01 - Dental assistants & therapists: 0.11 |
| Niger | Full integration^37,38^ | No data | No data | - Domestic general government expenditure: US$ 0.001 - Domestic private expenditure: US$ 0.046 - External expenditure: US$ 0.005 | US$ 0.0 | - Dentists: 0.01 - Dental prosthetic technicians: no data - Dental assistants & therapists: no data |
| North Korea | No integration^39^ | No data | No data | No data | US$ 3.5 | - Dentists: 2.19 - Dental prosthetic technicians: no data - Dental assistants & therapists: no data |
| Rwanda | Full integration^40-46^ | Tooth extraction, dental abscess drainage, cleft lip and palate repair, dental filling, dentures and prosthesis, maxillofacial trauma treatment, oral disease preventive service^†^*, oral surgery, salivary disease treatment, dental scaling^†^, oral disease screening^†^, orthodontics for dental condition^42-44^ | 70.6% of the nationally surveyed participants never visited an oral health provider for treatment^60^ | No data | US$ 0.3 | - Dentists: 0.18 - Dental prosthetic technicians: no data - Dental assistants & therapists: no data |
| Sierra Leone | Partial integration^47,48^ | Tooth extraction, dental filling, dentures and prosthesis, oral health knowledge awareness^†^, oral tumor treatment, dental trauma treatment, oral surgery, atraumatic restorative treatment^47^ | - Most nationally surveyed 12- (66%) and 15-year-olds (73%) reported ‘never having been to a dentist’, with only 8% having attended a check-up in both age-groups^61^ - Attendance amongst nationally surveyed 6-year-olds was lower, with only 3% of parents reporting their child had ever visited a dentist, and visited only when they had a problem^61^ | - Domestic general government expenditure: US$ 0.003 - Domestic private expenditure: US$ 1.302 - External expenditure: US$ 0.006 | US$ 0.2 | - Dentists: 0.02 - Dental prosthetic technicians: 0.64 - Dental assistants & therapists: 0.08 |
| Somalia | Partial integration^49^ | Tooth extraction, dental abscess drainage, oral emergency service^49^ | No data | No data | US$ 0.2 | - Dentists: no data - Dental prosthetic technicians: no data - Dental assistants & therapists: no data |
| South Sudan | No integration^50^ | No data | No data | - Domestic general government expenditure: US$ 0.618 - Domestic private expenditure: US$ 0.069 - External expenditure: US$ 0.038 | US$ 0.1 | - Dentists: 0.03 - Dental prosthetic technicians: <0.01 - Dental assistants & therapists: 0.02 |
| Sudan | No integration^51^ | Basic dental care*^51^ | - 64.6% of the nationally surveyed participants never used dental care; 22.0% had more than 12-month use, and 13.4% had past 12- month use^62^ - 7.6% participants who were ≥ 60 years old and surveyed in healthcare institutions in Khartoum State, Sudan, reported never received dental treatment, 37% had a dental visit within one year or less, and 55.4% had a dental visit more than one-year before^63^ - Over sixty percent of participants who were ≥ 16 years old and surveyed in healthcare institutions in seven provinces of the Khartoum State, Sudan, reported that they went to the dentist less frequently than every 2 years, 16.7% went more frequently than every 2 years, and 22.7% never went, indicating poor attendance^64^ | - Domestic general government expenditure: US$ 0.000 - Domestic private expenditure: US$ 0.148 - External expenditure: 0.000 | US$ 1.1 | - Dentists: 2.13 - Dental prosthetic technicians: no data - Dental assistants & therapists: no data |
| Syrian Arab Republic | No integration | No data | No data | No data | US$ 1.7 | - Dentists: 6.61 - Dental prosthetic technicians: no data - Dental assistants & therapists: no data |
| Togo | Partial integration^52,53^ | No data | No data | No data | US$ 0.2 | - Dentists: 0.02 - Dental prosthetic technicians: no data - Dental assistants & therapists: 0.03 |
| Uganda | Full integration^54,55^ | No data | Among the randomly sampled participants who were ≥ 18 years old in Nebbi district, Uganda, of 51.5% of them who had experienced a toothache or discomfort 12 months prior to the study, only about half (52%) had sought healthcare from a dental clinic or facility^65^ | - Domestic general government expenditure: US$ 0.030 - Domestic private expenditure: US$ 0.168 - External expenditure: no data | US$ 0.3 | - Dentists: 0.06 - Dental prosthetic technicians: 0.01 - Dental assistants & therapists: 0.21 |
| Yemen | No integration^56^ | No data | No data | No data | US$ 0.3 | - Dentists: 0.20 - Dental prosthetic technicians: no data - Dental assistants & therapists: 0.13 |

*. No further information on specific intervention provided.

†. Preventive oral health care service.

**Reference:**

1 Islamic Republic of Afghanistan. Ministry of Public Health. The Essential Package of Hospital Services for Afghanistan 2005/1384. Available from <https://platform.who.int/docs/default-source/mca-documents/policy-documents/guideline/afg-cc-46-01-guideline-2005-eng-essential-hospital-services.pdf>. Access date: May 2023.

2 World Health Organization. WHO MiNDbank. Available from <https://extranet.who.int/mindbank/item/5613>. Access date: May 2023.

3 Islamic Republic of Afghanistan 2011. Ministry of Public Health. Afghanistan National Health Workforce Plan 2012-2016. Available from <https://extranet.who.int/countryplanningcycles/sites/default/files/planning_cycle_repository/afghanistan/afghanistan_hrhplan_2012-2016_draft.pdf>. Access date: May 2023.

4 Islamic Republic of Afghanistan. Ministry of Public Health. National Reproductive, Maternal, Newborn, Child, and Adolescent Health (RMNCAH) Strategy 2017-2021. Available from <https://rmncah-moph.gov.af/wp-content/uploads/2017/11/National-RMNCAH-Strategy-2017-2021-English-Final.pdf>. Access date: May 2023.

5 Islamic Republic of Afghanistan. Ministry of Public Health. Normative Costing of Basic Package of Health Services (BPHS) 2020. Available from <https://moph.gov.af/sites/default/files/2020-11/BPHS%20Costing%20final%20Report%207-Nov-%202020_.pdf>. Access date: May 2023.

6 Burkina Faso. Ministere De La Sante. Programme National Des Maladies Non Transmissibles Unite De Sante Orale. Plan D’action Triennal De Lutte Contre Les Maladies Bucco-Dentaires Et Le Noma 2015 – 2017 [Burkina Faso. Ministry of Health. National Non-Communicable Diseases Program Oral Health Unit. Three-Year Action Plan for the Fight Against Oral Diseases and Noma 2015-2017]. Available from <https://extranet.who.int/ncdccs/Data/BFA_B8_Plan%20%20d'Action%20National%20%20Triennal%20Noma%20BURKINA%20FASO%20VF.pdf>. Access date: May 2023.

7 Burkina Faso. Ministere De La Sante. Plan stratégique de santé des personnes âgées 2016 – 2020 [Burkina Faso. Ministry of Health. Strategic Plan for the Health of the Elderly 2016-2020]. Available from <https://extranet.who.int/countryplanningcycles/sites/default/files/planning_cycle_repository/burkina_faso/plan_strategique_de_sante_des_personnes_agees_2016_-_2020.pdf>. Access date: May 2023.

8 Burkina Faso. Ministere De La Sante. Plan Strategique Integre De Lutte Contre Les Maladies Non Transmissibles 2016-2020 [Burkina Faso. Ministry of Health. Integrated Strategic Plan for the Fight Against Non-Communicable Diseases 2016-2020]. Available from <https://extranet.who.int/ncdccs/Data/BFA_B3_Plan%20SIMNT_FINAL_27-09-2016_F.pdf>. Access date: May 2023.

9 Republique Du Burundi. Ministere De La Sante Publique Et De La Lutte Contre Le Sida. Profil De Ressources Humaines En Sante Du Burundi 2011. [Republic of Burundi. Ministry of Public Health and the Fight Against AIDS. Profile of Human Resources in Health of Burundi 2011]. Available from <https://extranet.who.int/countryplanningcycles/sites/default/files/planning_cycle_repository/burundi/profil_rh_vf_22_janvier_20121.pdf>. Access date: May 2023.

10 Republique Du Burundi. Politique Nationale de Sante 2016-2025. [Republic of Burundi. National Health Policy 2016-2025] Available from <https://extranet.who.int/countryplanningcycles/sites/default/files/planning_cycle_repository/burundi/pns_2016_2025_burundi.pdf>. Access date: May 2023.

11 Republique Du Burundi. Ministere De La Sante Publique Et De La Lutte Contre Le Sida. Plan D’Action Multisectoriel De Prevention Et De Controle Des Maladies Non Transmissibles 2019-2023. [Republic of Burundi. Ministry of Public Health and the Fight Against AIDS. Multisectoral Action Plan for the Prevention and Control of Non-Communicable Diseases 2019-2023]. Available from <https://extranet.who.int/ncdccs/Data/BDI_B3_PAM%20FINAL%2023%2007%202019.pdf>. Access date: May 2023.

12 République Centr Africaine. Ministere De La Sante Publique. Document De Politique Nationale De Prevention Et De Lutte Contre Les Maladies Non Transmissibles 2014. [Central African Republic. Minister of Public Health. National Policy Document for the Prevention and Fight Against Non-Communicable Diseases 2014]. Available from <https://extranet.who.int/ncdccs/Data/CAF_B3_POLITIQUE%20MNT%20REPUBLIQUE%20CENTRAFRICAINE.pdf>. Access date: May 2023.

13 République Centr Africaine. Ministere De La Sante Publique. Plan de Transition du Secteur Santé en République Centrafricaine 2015-2017. [Central African Republic. Minister of Public Health. Health Sector Transition Plan 2015-2017]. Available from <https://extranet.who.int/countryplanningcycles/sites/default/files/planning_cycle_repository/central_african_republic/rca_-ptss_revise_final_26092016.pdf>. Access date: May 2023.

14 République du Tchad. Ministère de la Santé Publique. Plan Multisectoriel de Lutte et de Contrôle des Maladies Non Transmissibles 2017-2021. [Republic of Chad. Minister of Public Health. Multisectoral Plan for the Fight and Control of Non-Communicable Diseases 2017-2021] Available from <https://extranet.who.int/ncdccs/Data/TCD_B3_PLAN%20MULTISEC%20MNT.pdf>. Access date: May 2023.

15 République Démocratique du Congo. Ministere De La Sante. Plan Stratégique de la Santé Bucco-Dentaire 2021-2022. [Democratic Republic of Congo. Health Ministry. Oral Health Strategic Plan 2021-2022]. Available from <https://extranet.who.int/ncdccs/Data/COD_B8_s21_DRC_Plan%20Strategique%20SBD%202021%202022.docx>. Access date: May 2023.

16 Eritrea. Ministry of Health. The Second Health Sector Strategic Development Plan II 2017-2021. Available from <https://extranet.who.int/countryplanningcycles/sites/default/files/planning_cycle_repository/eritrea/eritrea_hssdp_ii_21022017.pdf>. Access date: May 2023.

17 Eritrea. Ministry of Health. Strategic Plan for the Implementation of Reproductive, Maternal, Newborn, Child and Adolescent Health and Healthy Ageing Programmes in Eritrea 2022-2026. Available from <https://www.afro.who.int/sites/default/files/2022-09/2022_RMNCAH_Eritrea.pdf>. Access date: May 2023.

18 Ethiopia. Ministry of Health. Health Sector Transformation Plan 2015-2020. Available from <https://extranet.who.int/countryplanningcycles/sites/default/files/planning_cycle_repository/ethiopia/hstp_ethiopia.pdf>. Access date: May 2023.

19 Ethiopia. Ministry of Health. Essential Health Services Package 2019. Available from <https://www.uib.no/sites/w3.uib.no/files/attachments/essential_health_service_package_ethiopia_2019_0.pdf>. Access date: May 2023.

20 Ethiopia. Ministry of Health. National Strategic Plan for the Prevention and Control of Major Non-Communicable Diseases 2021-2025. Available from <https://extranet.who.int/ncdccs/Data/ETH_B3_s21_National_Strategic_Plan_for_Prevention_and_Control_of_NCDs2021.pdf>. Access date: May 2023.

21 Gambia. Ministry of Employment and Social Welfare. National Ageing Policy Ageing with Security and Dignity 2010. Available from <https://extranet.who.int/countryplanningcycles/sites/default/files/planning_cycle_repository/gambia/national_ageing_policy_2010.pdf>. Access date: May 2023.

22 Gambia. Ministry of Health and Social Welfare. National Health Strategic Plan 2014-2020. Available from <https://extranet.who.int/countryplanningcycles/sites/default/files/planning_cycle_repository/gambia/gnhsp_-_final_draft_13oct2014.pdf>. Access date: May 2023.

23 Gambia. Ministry of Health. National Multi-Sectoral Strategy and Costed Action Plan for Non-Communicable Disease Prevention and Control in The Gambia 2022-2027. Available from <https://www.afro.who.int/sites/default/files/2022-07/National%20Multi-sectoral%20Strategy%20and%20Costed%20Action%20Plan%20for%20NCD%20prevention%20and%20control%20-Gambia%202022-27.pdf>. May 2023.

24 Guinee. Ministere De La Sante. Plan national de développement sanitaire 2015-2024. [Guinea. Ministry of Health. National Health Development Plan 2015-2024] Available from <https://extranet.who.int/countryplanningcycles/sites/default/files/public_file_rep/GIN_Guinea_Plan-national-de-developmment-sanitaire_2015-2024.pdf>. Access date: May 2023.

25 Guiné Bissau. Ministério Da Saúdepública. Programa Nacional de Luta contra Noma Plano de Ação das Atividades 2016. [Guinea-Bissau. Ministry of Public Health. National Program to Fight against Noma Action Plan 2016] Available from <https://extranet.who.int/ncdccs/Data/GNB_B8_Plan%20op%c3%a9rationnel%20NOMA2016_GNB.pdf>. Access date: May 2023.

26 Liberia. Ministry of Health. Essential Package of Health Services for Universal Health Coverage 2022. Available from <https://www.dcp-3.org/sites/default/files/resources/MOH%20EPHS%20for%20UHC_Final%20Version%2020221121.pdf>. Access date: May 2023.

27 Madagascar. Ministere De La Sante Publique. Politique Nationale de Sante Bucco-Dentaire 2010-2020. [Madagascar. Minister of Public Health. National Oral Health Policy 2010-2020]. Available from <https://extranet.who.int/ncdccs/Data/MDG_B8_PNSBD%20final_photos%2005_%20juin_10(1).doc>. Access date: May 2023.

28 Madagascar. Ministre de la Sante Publique. Plan de Développement du Secteur Santé 2015-2019. [Madagascar. Minister of Public Health. Health Sector Development Plan 2015-2019] Available from <https://extranet.who.int/countryplanningcycles/sites/default/files/planning_cycle_repository/madagascar/pdss_2015.pdf>. Access date: May 2023.

29 Madagascar. Ministre de la Santé Publique. Politique Nationale de Lutte Intégrée contre les Maladies Non Transmissibles et de Prévention du Handicap 2017. [Madagascar. Minister of Public Health. National Policy for Integrated Control of Non-Communicable Diseases and Prevention of Disability 2017] Available from <https://extranet.who.int/ncdccs/Data/MDG_B3_2%20DOC%20PolitiqueMNT_PH_020718_.pdf>. Access date: May 2023.

30 Madagascar. Ministre de la Santé Publique. Plan Stratégique National de Santé des Personnes Âgées 2018. [Madagascar. Minister of Public Health. National Strategic Plan for Health of the Elderly 2018] Available from <http://www.sante.gov.mg/ministere-sante-publique/wp-content/uploads/2021/07/Plan-Strat%C3%A9gique-National-de-la-Sant%C3%A9-des-Personnes-Ag%C3%A9es-Madagascar.pdf>. Access date: May 2023.

31 Malawi. Ministry of Health. Health Sector Strategic Plan II 2017-2022. Available from <https://extranet.who.int/countryplanningcycles/sites/default/files/planning_cycle_repository/malawi/health_sector_strategic_plan_ii_030417_smt_dps.pdf>. Access date: May 2023.

32 Mali. Ministère de la Santé et de l’Hygiène Publique. Ministère du Travail et des Affaires Sociales et Humanitaires. Ministère de la Promotion de la Femme, de la Famille et de l’Enfant. Plan Décennal de Développement Sanitaire et Social (PDDSS) 2014-2023. [Mali. Ministry of Health and Public Hygiene. Ministry of Labor and Social and Humanitarian Affairs. Ministry for the Promotion of Women, Families and Children. Ten-year Health and Social Development Plan 2014-2023]. Available from <https://www.childrenandaids.org/sites/default/files/2018-05/Mali_Nat%20Health%20Plan_2014-2023%20fr.pdf>. Access date: May 2023.

33 Mali. Plan Stratégique de Santé Bucco-Dentaire 2018-2022. [Mali. Oral Health Strategic Plan 2018-2022]. Available from <https://extranet.who.int/ncdccs/Data/MLI_B8_Plan%20d'Action%20Sant%c3%a9%20Bucco-Dentaire%202018-2022.pdf>. Access date: May 2023.

34 Mozambique. Ministry of Health. Health Sector Strategic Plan 2014-2019. Available from <https://extranet.who.int/countryplanningcycles/sites/default/files/planning_cycle_repository/mozambique/mozambique_-_health_sector_strategic_plan_-_2014-2019.pdf>. Access date: May 2023.

35 República De Moçambique. Ministério Da Saúde. Direcção Nacional De Assistência Médica Estratégia Nacional de Saúde Oral 2019-2024. [Mozambique. Ministry of Health. National Directorate of Medical Assistance National Oral Health Strategy 2019-2024]. Available from <https://extranet.who.int/ncdccs/Data/MOZ_B8_ESTRATEGIA%20NACIONAL%20DE%20SAUDE%20ORAL_%202019%20-%202024%20-final%20Moz.pdf>. Access date: May 2023.

36 República De Moçambique. Ministério Da Saúde. Plano Estratégico Multissectorial de Prevenção e Controlo de Doenças Não Transmissíveis 2020-2029. [Mozambique. Ministry of Health. Strategic Plan Multisectoral of Prevention and Control of Non-Communicable Diseases 2020-2029] Available from <https://extranet.who.int/ncdccs/Data/MOZ_B3_s21_Plano%20Estrat%c3%a9gico%20Multissetorial%20de%20Prevencao%20e%20Controlo%20das%20DNTs%202020-2029%20FINALISSIMA.pdf>. Access date: May 2023.

37 République Du Niger. Ministere De La Sante Publique. Plan Stratégique National Multisectoriel de Lutte contre les Maladies Non Transmissibles 2019-2021. [Niger. Ministry of Health. National Strategic Plan Multisectoral Fight Against Non-Communicable Diseases 2019-2021]. Available from <https://extranet.who.int/ncdccs/Data/NER_B3_s21_EXE%20-%20Doucument%20complet%20Valid%c3%a9%20PNLCMNT.pdf>. Access date: May 2023.

38 République Du Niger. Ministere De La Sante Publique. Plan Stratégique National Intégré de Lutte contre les Maladies Bucco-Dentaires et le Noma (PSNILMBD/N) 2021-2025. [Niger. Ministry of Health. Integrated National Strategic Plan for the Fight Against Oral Diseases and Noma 2021-2025]. Available from <https://extranet.who.int/ncdccs/Data/NER_B8_s21_PLAN%20Strat%c3%a9gique%20MBD-N.docx>. Access date: May 2023.

39 Democratic People's Republic of Korea. Ministry of Public Health. Medium Term Strategic Plan for the Development of the Health Sector DPR Korea 2016-2020. Available from <https://extranet.who.int/countryplanningcycles/sites/default/files/planning_cycle_repository/democratic_peoples_republic_of_korea/dpr_korea_medium_term_strategic_plan_2016-20.pdf>. Access date: May 2023.

40 Rwanda. Ministry of Health. National Community Health Strategic Plan 2013-2018. Available from <https://extranet.who.int/mindbank/item/7440>. Access date: May 2023.

41 Rwanda. Ministry of Health. Rwanda Non-Communicable Diseases Policy 2015. Available from <https://extranet.who.int/ncdccs/Data/RWA_B3_NCDs_Policy.2015.pdf>. Access date: May 2023.

42 Rwanda. Ministry of Health. Health Service Packages for Public Health Facilities 2017. Available from <https://www.moh.gov.rw/fileadmin/user_upload/Moh/Publications/Legal_Framework/Public_health_Facilities_service_packages_in_Rwanda-1.pdf>. Access date: May 2023.

43 Rwanda. Ministry of Health. Fourth Health Sector Strategic Plan 2018-2024. Available from <https://extranet.who.int/mindbank/item/7442>. Access date: May 2023.

44 Rwanda. Ministry of Health. Service Packages for Upgraded Health Centers Rwanda Health Care System 2019. Available from <https://www.moh.gov.rw/index.php?eID=dumpFile&t=f&f=11803&token=c8fe376a7aa067259c7fe35ce5d7c6e078c74f5b>. Access date: May 2023.

45 Rwanda. Ministry of Health. National Oral Health Strategic Plan 2019-2024. Available from <https://moh.prod.risa.rw/fileadmin/user_upload/Moh/Publications/Strategic_Plan/National_Oral_Health_Strategic_Plan_2019-2024.pdf>. Access date: May 2023.

46 Rwanda. 10-year Government Program: National Strategy for Health Professionals Development 2020-2030. Available from <https://www.rbc.gov.rw/fileadmin/user_upload/strategy/RWANDA%20National%20Strategy%20for%20Health%20Professions%20Development%20%28NSHPD%202020-2030%29.pdf>. Access date: May 2023.

47 Sierra Leone. Ministry of Health and Sanitation. Basic Package of Essential Health Services 2015-2020. Available from <https://mohs2017.files.wordpress.com/2017/06/gosl_2015_basic-package-of-essential-health-services-2015-2020.pdf>. Access date: May 2023.

48 Sierra Leone. Ministry of Health and Sanitation. Non-Communicable Disease (NCDs) Strategic Plan 2020-2024. Available from <https://extranet.who.int/ncdccs/Data/SLE_B3_s21_NCD%20strategic%20plan%202020-2024%2023Feb2020%20FINAL%20signed%20CF%20(1).docx>. Access date: May 2023.

49 Somalia. Ministry of Health and Human Services. Essential Package of Health Services 2020. Available from <https://reliefweb.int/attachments/981c3ca3-6914-3d40-824a-bac5889d906c/somalia_ephs_web.pdf>. Access date: May 2023.

50 South Sudan. Ministry of Health. National Health Policy 2016-2026. Available from <https://extranet.who.int/countryplanningcycles/sites/default/files/planning_cycle_repository/south_sudan/south_sudan_national_health_policy_2016_to_2025_2.pdf>. Access date: May 2023.

51 Sudan. National Health Sector Strategic Plan Ⅱ 2012-2016. Available from <https://extranet.who.int/countryplanningcycles/sites/default/files/planning_cycle_repository/sudan/sudan_national_health_sector_strategic_plan_nhssp_2012-2016.pdf>. Access date: May 2023.

52 République Togolaise. Ministere De La Sante Et De La Protection Sociale. Plan National de Developpment Sanitaire 2017-2022. [Togo. Ministry of Health and Social Protection. National Health Development Plan 2017-2022] Available from <https://extranet.who.int/countryplanningcycles/sites/default/files/planning_cycle_repository/togo/togo_pnds_2017-2022_version_definitive_210217_en_edition.pdf>. Access date: May 2023.

53 République Togolaise. Ministere De La Sante Et De L’Hygiene Publique. Politique et Plan Stratégique Multisectoriel de Lutte contre les Maladies Non Transmissibles 2018-2022 [Togo. Ministry of Health and Public Hygiene. Policy and Multisectoral Strategic Plan for the Prevention and Control of Non-Communicable Diseases 2018-2022]. Available from <https://extranet.who.int/countryplanningcycles/sites/default/files/planning_cycle_repository/togo/togo_pnds_2017-2022_version_definitive_210217_en_edition.pdf>. Access date: May 2023.

54 Uganda. Ministry of Health. Health Sector Development Plan 2015-2020. Available from <https://extranet.who.int/countryplanningcycles/sites/default/files/planning_cycle_repository/uganda/health_sector_development_plan_2015-16_2019-20_0.pdf>. Access date: May 2023.

55 Uganda. Ministry of Health. National Oral Health Policy. Available from <https://extranet.who.int/ncdccs/Data/UGA_B8_National%20Oral%20Health%20Policy.pdf>. Access date: May 2023.

56 Yemen. Ministry of Public Health & Population. National Health Strategy 2010-2025. Available from <https://extranet.who.int/countryplanningcycles/sites/default/files/planning_cycle_repository/yemen/nat_health_strategy_-_yemen_eng.pdf>. Access date: May 2023.

57 Diendéré J, Ouattara S, Kaboré J, Traoré I, Zeba AN, Kouanda S. Oral hygiene practices and their sociodemographic correlates among adults in Burkina Faso: results from the First National Survey. BMC Oral Health. 2022 Dec;22(1):1-6.

58 Bassa S, Workie SB, Kassa Y, Tegbaru DW. Prevalence of dental caries and relation with nutritional status among school-age children in resource limited setting of southern Ethiopia. BMC Oral Health. 2023 Feb 10;23(1):84.

59 Mohammed HM, Mehari MA, Asgedom AA. Predictors of low dental service utilization among school children in Mekelle, Northern Ethiopia: a cross-sectional study. BMC Oral Health. 2023 Jan 25;23(1):41.

60 Morgan JP, Isyagi M, Ntaganira J, Gatarayiha A, Pagni SE, Roomian TC, Finkelman M, Steffensen JE, Barrow JR, Mumena CH, Hackley DM. Building oral health research infrastructure: the first national oral health survey of Rwanda. Global Health Action. 2018 Jan 1;11(1):1477249.

61 Ghotane SG, Challacombe SJ, Don-Davis P, Kamara D, Gallagher JE. Unmet need in Sierra Leone: a national oral health survey of schoolchildren. BDJ open. 2022 Jun 14;8(1):16.

62 Pengpid S, Peltzer K. Prevalence and correlates of dental service utilisation among a national general adult population sample in Sudan. BMC oral health. 2021 Dec;21(1):1-8.

63 Salih MA, Ali RW, Nasir EF. Oral health status and associated factors among Sudanese older adults: A cross‐sectional study. Gerodontology. 2022 Dec;39(4):408-17.

64 Khalifa N, Allen PF, Abu-bakr NH, Abdel-Rahman ME, Abdelghafar KO. A survey of oral health in a Sudanese population. BMC Oral Health. 2012 Dec;12:1-9.

65 Ocwia J, Olum R, Atim P, Laker F, Okot J, Sereke SG, Baluku JB, Kiguli S, Bongomin F. Oral health seeking behaviors of adults in Nebbi District, Uganda: a community-based survey. BMC oral health. 2021 Dec;21:1-7.

66 World Health Organization. Global Health Expenditure Database. Available from <https://apps.who.int/nha/database/Select/Indicators/en>. Access date: May 2023.

67 World Health Organization. Global oral health status report. Country profiles. Available from <https://www.who.int/team/noncommunicable-diseases/global-status-report-on-oral-health-2022>. Access date: May 2023.

68 World Health Organization. National Health Workforce Account. Available from <https://apps.who.int/nhwaportal/Home>. Access date: May 2023.
